# Decoding human-macaque interspecies differences in Fc-effector functions: The structural basis for CD16-dependent effector function in Rhesus macaques

**DOI:** 10.3389/fimmu.2022.960411

**Published:** 2022-09-05

**Authors:** William D. Tolbert, Neelakshi Gohain, Paul G. Kremer, Andrew P. Hederman, Dung N. Nguyen, Verna Van, Rebekah Sherburn, George K. Lewis, Andrés Finzi, Justin Pollara, Margaret E. Ackerman, Adam W. Barb, Marzena Pazgier

**Affiliations:** ^1^ Infectious Disease Division, Department of Medicine, Uniformed Services University of the Health Sciences, Bethesda, MD, United States; ^2^ Institute of Human Virology, University of Maryland School of Medicine, Baltimore, MD, United States; ^3^ Department of Biochemistry and Molecular Biology, University of Georgia, Athens, GA, United States; ^4^ Thayer School of Engineering, Dartmouth College, Hanover, NH, United States; ^5^ Centre de recherche du CHUM, Montreal, QC, Canada; ^6^ Département de Microbiologie, Infectiologie et Immunologie, Université de Montréal, Montreal, QC, Canada; ^7^ Department of Surgery, Duke University School of Medicine, Durham, NC, United States; ^8^ Human Vaccine Institute, Duke University School of Medicine, Durham, NC, United States; ^9^ Center for Human Systems Immunology, Duke University School of Medicine, Durham, NC, United States; ^10^ Complex Carbohydrate Research Center, University of Georgia, Athens, GA, United States; ^11^ Department of Chemistry, University of Georgia, Athens, GA, United States

**Keywords:** Rhesus macaques *Macaca mulatta*, FcγRIII Val/Ile^158^, CD16, Fc-effector function, IgG1(Fc)- FcγRIII complex structure - function of RM FcγRIII Ile/Val 158

## Abstract

Fc mediated effector functions of antibodies play important roles in immunotherapies and vaccine efficacy but assessing those functions in animal models can be challenging due to species differences. Rhesus macaques, *Macaca mulatta* (Mm) share approximately 93% sequence identity with humans but display important differences in their adaptive immune system that complicates their use in validating therapeutics and vaccines that rely on Fc effector functions. In contrast to humans, macaques only have one low affinity FcγRIII receptor, CD16, which shares a polymorphism at position 158 with human FcγRIIIa with Ile^158^ and Val^158^ variants. Here we describe structure-function relationships of the Ile/Val^158^ polymorphism in Mm FcγRIII. Our data indicate that the affinity of the allelic variants of Mm FcγRIII for the macaque IgG subclasses vary greatly with changes in glycan composition both on the Fc and the receptor. However, unlike the human Phe/Val^158^ polymorphism in FcγRIIIa, the higher affinity variant corresponds to the larger, more hydrophobic side chain, Ile, even though it is not directly involved in the binding interface. Instead, this side chain appears to modulate glycan-glycan interactions at the Fc/FcγRIII interface. Furthermore, changes in glycan composition on the receptor have a greater effect for the Val^158^ variant such that with oligomannose type glycans and with glycans only on Asn^45^ and Asn^162^, Val^158^ becomes the variant with higher affinity to Fc. These results have implications not only for the better interpretation of nonhuman primate studies but also for studies performed with human effector cells carrying different FcγRIIIa alleles.

## Introduction

Fc-mediated effector mechanisms utilize host effector cells (natural killer (NK) cells, macrophages, monocytes or eosinophils) to inactivate foreign cells (cancer cells, viruses, bacteria or infected cells) that are opsonized by antibody. In these mechanisms, the effectiveness of inactivation depends upon Fc gamma receptors (FcγRs) expressed at the surface of effector cells and the interaction of the extracellular domain of the FcγR with the Fc portion of the antibody bound to its cognate epitope. As a major contributor to the humoral immune response, Fc-effector functions are recognized as protective mechanisms against infectious pathogens. Importantly, Fc-effector functions, primarily antibody dependent cellular cytotoxicity (ADCC), have been implicated in the protective effect against HIV-1 infection in the RV144 vaccine trial, the only vaccine trial in humans to show any protective efficacy (31.2%) (1–4). Fc-effector functions have also been shown to correlate with protection elicited by diverse vaccine regimens tested in non-human primates (NHP) against simian immunodeficiency virus (SIV) or simian/human immunodeficiency virus (SHIV) infection (5–8). Although the existing data support a role for FcγR-dependent effector functions in protection in both human and NHP studies, direct comparisons among study outcomes are not straightforward due to species differences in immune function. Despite evolutionary proximity, there are significant differences between humans and NHPs in both the genetic and functional characteristics of their immune system components, e.g. FcγRs expressed on effector cells and IgG subclasses, which make the interpretation of NHP studies difficult to the point that they may not translate to humans (8–11). The fact that testing new vaccine concepts in humans is often not practical or ethical makes understanding the immune system differences between humans and NHP, such as the functional interplay between FcγRs and Fcs, critical for the development of more effective vaccines or therapeutics against HIV-1 and other infectious diseases.

In humans, antibody-mediated effector functions depend on activation of the lower affinity receptors, FcγRIIa and c (or CD32a and c) and FcγRIIIa and b (or CD16a and b). A gene duplication event in primates gave rise to FcγRIIa from FcγRIIb, the only inhibitory Fcγ receptor. Subsequent disruption of the intracellular immunoreceptor tyrosine-based inhibitory motif (ITIM) in FcγRIIa by a transposable element changed the ITIM an intracellular immunoreceptor tyrosine-based activation motif (ITAM) which consequently made FcγRIIa an activating receptor (12). FcγRIIa therefore has a non-canonical ITAM. FcγRIIa is the most widely expressed FcγR and is present on macrophages and neutrophils that are largely involved in the phagocytosis of antibody-opsonized antigens (12, 13). A second gene duplication event in higher primates disrupted the ITAM of FcγRIIa giving rise to both FcγRIIc and FcγRIIIb (12). FcγRIIc retained the non-canonical ITAM of FcγRIIa and is present on Natural Killer (NK) cells but is only expressed in a small fraction of individuals due to a premature stop codon in the coding sequence of the most common allele (14, 15). FcγRIIIb (or CD16b) lost its ability to interact with ITAM-containing partners in the gene duplication event and is a glycophosphatidylinositol (GPI) linked receptor mainly present on neutrophils and basophils (16). Because FcγRIIIb lacks a transmembrane domain and can no longer recruit ITAM-containing partners like FcγRIIIa, its mechanism of activation remains a matter of debate. FcγRIIIb activation is thought to be mediated in conjunction with FcγRIIa which is also expressed on neutrophils (17), but lipid raft-mediated activation of SHP-2 (Src homology region 2-containing protein tyrosine phosphatase 2) by FcγRIIIb has also been shown independent of FcγRIIa with changes in cytokine expression and reduced apoptosis (18). FcγRIIa activation on neutrophils leads to cleavage of FcγRIIIb from the cell surface by ADAM17 of the ADAM (a disintegrin and metalloprotease) family of membrane-associated proteases (19) making FcγRIIIb the major soluble FcγRIII in human serum. FcγRIIIa (CD16a) on the other hand, analogous to FcγRIII in macaques, lacks an ITAM and associates with the FcγR γ-chain or with the TCRζ chain that provides an ITAM activation motif. FcγRIIIa is present on NK cells, monocytes, macrophages, and dendritic cells.

Because FcγRIIIa (CD16a) is expressed on the surface of NK cells, it is a major component of the innate cellular immune response. The extracellular domain of human FcγRIIIa consists of two immunoglobulin-like folds, domains 1 and 2 (d1 & d2), spanning residues 1-174 which directly interact with the Fc of the antigen bound antibody. The extracellular domains are glycosylated with a total of five N-linked glycosyl groups: Asn^38^, Asn^45^, Asn^74^, Asn^162^, and Asn^169^. There are two polymorphic alleles in the extracellular domain of human FcγRIIIa, a higher affinity Val^158^ allele and a lower affinity Phe^158^ allele (13). The Val/Phe^158^ difference is within the extracellular region that directly contributes to the binding interface with IgG Fc. In contrast, there are four alleles of FcγRIIIb, the NA2 allele (also known as HNA-1b) which differs from FcγRIIIa at four positions in the extracellular domain (Arg^18^ to Ser, Asp^64^ to Asn, Gly^129^ to Asp, and Tyr^140^ to His [residue numbering relative to FcγRIIIa with no signal peptide sequence]), the SH allele (HNA-1c) which contains one additional difference (Ala^60^ to Asp), the HNA-1d allele which contains one fewer change relative to FcγRIIIa with an Asp^64^, and the NA1 allele (HNA-1a) which differs from NA2 by two additional changes (Ser^47^ to Asn and Ile^88^ to Val) but like HNA-1d and FcγRIIIa has an Asp^64^ (20). All FcγRIIIb alleles have Val^158^ but exhibit allelic differences in glycosylation. FcγRIIIb has an approximately 10-fold lower affinity towards IgG1 than FcγRIIIa, which can largely be attributed to the Gly^129^ to Asp difference in sequence (21).

Importantly, the gene duplication event that gave rise to FcγRIIc and FcγRIIIb in humans occurred after the split between macaques and other higher primates (12). As a consequence, many NHPs, including Rhesus macaques, *Macaca mulatta* (Mm), a major primate species used in animal studies, lack a FcγRIIIb gene. The only FcγRIII variant expressed in Mm, hereafter referred to as Mm FcγRIII (10), has the greatest amino acid sequence identity to human FcγRIIIa, with 11 residue changes in the extracellular domain, three of which correspond to Fc contact residues, but lacks the glycan at position 74 due to a change from Asn to Ser. However, similar to the FcγRIIIb NA2 and SH alleles, it also has an added a glycosylation site at position 64, giving it the same total number of glycosylation sites as human FcγRIIIa, five: Asn^38^, Asn^45^, Asn^64^, Asn^162^, and Asn^169^. In this regard, Mm FcγRIII shares some sequence features with both human FcγRIIIa and b. Mm FcγRIII, like human FcγRIIIa, has two variants with amino acid differences at position 158 in the extracellular domain; there are additional polymorphisms in the intracellular domain. The most common Mm FcγRIII variants have Ile at position 158 which is replaced by Val in a minority of macaques (approximately 94% Ile^158^ versus 4.4% Val^158^ in one cohort of 155 macaques (22)). There is limited information about the functional consequence of the Val/Ile^158^ polymorphism in Mm, in particular its contribution to antibody mediated effector function and ADCC. Recent comprehensive biophysical characterization of IgG affinity towards Mm FcγRs indicates that the Ile^158^ variant exhibits slightly higher affinity to both macaque and human IgG (10, 23). In contrast, functional analyses show a different pattern with Ile/Val at position 158 having little effect on Fc-effector activity (22). This is different to humans, where the Val^158^ variant has higher affinity for the human IgG1 than the Phe^158^ variant (24) which translates to significantly decreased activity for the Phe^158^ variant with regard to Fc-effector functions.

Here, we fill a major gap in understanding of the molecular details of FcγIII functions in Mm. These results are significant because the FcγRIII variant is expressed on Mm effector cells and plays a fundamental role in antibody mediated effector function in this species. We performed comprehensive biochemical, functional, and structural studies of the polymorphic Ile^158^ and Val^158^ variants of the receptor to describe their differences in affinity for the Mm IgG subclasses, the differences in their ability to mediate ADCC, and the molecular details of the receptor-Fc IgG1 interaction. Complex crystal structures formed between the Mm FcγRIII Ile^158^ and Val^158^ variants with the Fc of Mm IgG1 and the crystal structure of the apo form of the glycosylated Ile^158^ variant, coupled with MS and NMR-based studies indicate differences in the intermolecular glycan-glycan interactions formed between receptor and Fc for both variants. Altogether, our studies indicate important immune system differences between humans and macaques that become critical when the latter are used to evaluate new vaccines and antibody therapeutics that involve or depend upon Fc-effector functions for their biological effects.

## Material and methods

### Fc and FcγRIII expression and purification

The macaque FcγRIII(Ile^158^) extracellular domain residues 1-174 (1-191 including the leader sequence) with two mutations to remove two of the five predicted glycosylation sites, Asn^38^ to Gln and Asn^169^ to Gln, and the macaque immunoglobulin IgG1 Fc (residues 237-447) were synthesized with the macaque FcγRIII leader sequence by Blue Heron Biotech, LLC and cloned into the pCMV6-A-Puro expression vector. The FcγRIII(Val^158^) allelic variant was generated by site directed mutagenesis of the FcγRIII(Ile^158^) plasmid using the QuikChange site-directed mutagenesis kit (Stratagene). Macaque FcγRIII receptor was prepared by transfection of GnT1^-^ HEK 293F Freestyle cells (Life Technologies) with 0.5 mg of plasmid/liter of culture and expressed in Freestyle 293 medium (Life Technologies) supplemented with 2.5% Ultra Low IgG Fetal Bovine Serum (FBS) (Gibco) for 6 days. Macaque IgG1 Fc was prepared by transfection of HEK 293F Freestyle cells with 0.5 mg of plasmid/liter of culture and expressed in Freestyle 293 medium for 6 days. The FcγRIII receptor was purified from medium passed over IgG sepharose 6 fast flow affinity resin (GE Healthcare) equilibrated with phosphate buffered saline (PBS) pH 7.2. The Fc was purified from medium passed over a HiTrap protein A column (GE Healthcare) equilibrated with PBS.

The purification protocol for both columns was similar. After the medium was passed over the column the column was washed with PBS and the protein was eluted with 0.1 M glycine pH 3.0. Eluted fractions were immediately diluted 10:1 with 1 M Tris-HCl pH 8.5. Eluted protein was concentrated, and the buffer exchanged to PBS pH 7.2. Receptor and Fc were further purified by size exclusion chromatography over a Superdex 200 gel filtration column (GE Healthcare) equilibrated with 25 mM Tris-HCl pH 8.5 and 150 mM sodium chloride. Elution fractions of the receptor were collected and concentrated to approximately 10 mg/ml for use in crystallization trials or in complex formation with the Fc. Prior to complex formation with macaque IgG1 Fc glycans were removed from the Ile^158^ receptor variant by deglycosylaion with EndoH_f_ (New England Biolabs) but complex formation with the Va^l158^ receptor variant proceeded with the glycans intact. EndoH_f_ was removed after the deglycosylation reaction by passage over an amylose column. Receptor and Fc were mixed at roughly 1 to 1 stoichiometry and the receptor complex purified by Superdex 200 gel filtration. Elution fractions corresponding to the complex molecular weight were combined and concentrated to 10 mg/ml for use in crystallization trials.

### Generation of NK-92 Mm FcγRIII cell lines

NK-92 cells (ATCC CRL-2407) are a human NK-cell line derived from an individual with malignant non-Hodgkin lymphoma. The cells have natural cytotoxicity activity but lack expression of FcγRIIIa (CD16). NK-92 cells that stably express Mm FcγRIII were produced using the Amphotropic Platinum Retrovirus Expression System (Cell Biolabs, San Diego, CA). Briefly, the extracellular domain of the rhesus macaque *FCGR3A* gene (GenBank JQ038005.1, Ile^158^), and the Val^158^ variant, were synthesized as a chimera with the transmembrane and intracellular signaling domain of human FcεRI, similar to that previously described (25). This construct was subcloned into plasmid pMXs-Puro retroviral expression vector using BamHI and NotI sites and transfected into the Plat-A retrovirus packaging cell line using FuGENE (Promega, Madision, WI). Supernatant containing retrovirus was used to transduce NK-92 cells, and flow cytometry cell sorting with anti-CD16 antibody (clone 3G8, BD Biosciences, San Jose, CA) was used to select cells that expressed receptor on the cell surface. Clonal populations were isolated by expansion after single-cell dilution, the sequence of the inserted gene was confirmed by targeted PCR amplification and Sanger sequencing (Genewiz, Morrisville, NC), and stability of cell-surface expression of CD16 was confirmed by regular monitoring by flow cytometry.

### ADCC against HIV envelope-coated target cells

The ADCC-GranToxiLux (GTL) assay was performed similar to previously described (26, 27). Target cells were a clonal isolate of the CEM.NKR_CCR5_ CD4^+^ T cell line (NIH AIDS Reagent Program, Division of AIDS, NIAID, NIH: from Dr. Alexandra Trkola (28)) coated with HIV-BaL gp120 envelope protein (Immune Technologies Corp., New York). NK-92 Mm FcγRIII cells were used as effector cells at an effector cell to target cell ratio of 10:1. Monoclonal antibodies were tested after four-fold serial dilutions starting at 10 μg/ml. Data were reported as the maximum observed proportion of cells positive for proteolytically active granzyme B (GzB) out of the total viable target cell population (maximum %GzB activity) after subtracting the background activity observed in wells containing effector and target cells in the absence of antibodies.

### Receptor affinity determinations

Binding affinities were measured using biolayer interferometry (BLI) using the Forte Bio Octet system. Following buffer exchange into PBS as needed, Mm IgG subclasses (NHP Reagent Resource) and Mm IgG Fc domains expressed and purified as described above, were biotinylated with LC-LC no weigh biotin (Thermo Fisher, A39257) at a 10M excess concentration. After 30 min of reaction time, excess biotin was removed with Zeba desalting columns (Thermo Fisher, 89882). To determine binding kinetics, biotinylated IgGs and Fcs were captured on streptavidin Sax-2.0 tips (Forte Bio, 18-5136). Briefly, biosensors were first equilibrated in PBST (0.05% Tween-PBS) for 180s and activated by dipping into 10 mM glycine (pH 1.7) for 20s and PBST 20s for three cycles. Biosensors were then loaded with biotinylated IgG or Fc domains at 1mg/ml in PBST for 300s, and dipped into PBST for 300s to reach baseline, prior to a 300s association phase in which they were dipped into FcγR, and a 300s dissociation phase in which they were dipped into PBST. Assessments were performed across 3-fold serial dilutions of FcγR ranging from 10 mM to 0.013 mM. Tips were regenerated for 20s for each condition in 10mM glycine pH 1.7. Data was aligned and corrected between steps as needed, and signal observed in reference sample wells, comprised of tips loaded with IgG antibody but not dipped into receptor, was subtracted. For kinetic analysis, a 1:1 association and dissociation model was selected, and for equilibrium fits, a 5s window from the end of the association phase was used to define the equilibrium level of signal for each condition in Forte Bio data analysis 7.0 software in order to determine K_D,_ association constant (K_on_) and dissociation constant (k_off_) (**Figure S1** and **Table S1**). Equilibrium and kinetic fits showed good agreement, and raw data was plotted in GraphPad Prism v9.0.

### Crystallization and data collection

Crystals were initially grown from commercial crystallization screens and later optimized to produce crystals suitable for data collection. Receptor crystals were grown from 25% PEG 3350 and 0.1 M HEPES pH 7.0. Ile^158^ variant complex crystals were grown from 15% PEG 4000, 0.1 M HEPES pH 7.0 and 0.1 M magnesium chloride and Val^158^ variant complex crystals grown from 20% PEG 4000, 0.1 M MES pH 6.0, and 0.2 M lithium sulfate. Prior to data collection crystals were briefly soaked in crystallization buffer supplemented with 20% MPD and then flash frozen in liquid nitrogen.

Diffraction data were collected at the Stanford Synchrotron Radiation Light Source (SSRL) BL12-2 (apo Ile^158^ variant receptor and Ile^158^ variant complex) and BL9-2 (Val^158^ variant complex) beamlines on Dectris Pilatus 6M area detectors. All data were processed and reduced with HKL2000 (29). Structures were solved by molecular replacement with PHASER from the CCP4 suite (30) based on the coordinates of the human FcγRIIIa and human IgG1 Fc (PDB ID 3SGJ for FcγRIII and 3AVE for MmIgG1 Fc). Refinement was carried out with Refmac (30) and/or Phenix (31) and model building was done with COOT (30). Data collection and refinement statistics are shown in **Table 2**. Ramachandran statistics were calculated with MolProbity, and illustrations were prepared with Pymol Molecular graphics (http://pymol.org).

### Mass spectrometry

Following purification, each protein (10 µg) was suspended in 50 µL of 50 mM ammonium carbonate and 10% methanol (v/v), pH 8.0. Samples were heated at 95°C for 5 min and then cooled on ice. Fresh dithiothreitol (DTT) was added to a final concentration of 5 mM. The samples were heated at 37°C for 1 h. Next, freshly resuspended iodoacetamide was added to a final concentration of 14 mM and the samples were incubated at room temperature in the dark for 30 min. Additional DTT was added (1 µL of 0.5 M) to quench excess iodoacetamide followed by a final incubation at RT for 15 min in the dark. Chymotrypsin and Glu-C was added to each sample at a final concentration of 1 mg/mL and incubated overnight at 37°C. The following day, an additional aliquot of each enzyme was added and further incubated for 2 h. Glycopeptides from each sample were enriched using TopTip PolyHydroxyethyl A (HILIC) 10 µL tips (Glygen) following manufacturer guidelines. Samples were eluted from the resin three times into the same receptacle with 10 µL elutions of 15 mM ammonium acetate, 10% acetonitrile (v/v) pH 3.5. The resulting elutions were lyophilized and then desalted with C18 ZipTips (Millipore) according to the manufacturer’s protocol. The samples were resuspended in 20 µL of deionized water. Each sample (0.5 µL) was loaded into an EASY nLC-1200 LC system with PepMap nanocolumn (ThermoFisher) and a Nanospray FlexIon source (ThermoFisher) and analyzed with a Q Exactive Plus Hybrid Quadrupole-Orbitrap Mass Spectrometer (ThermoFisher). Liquid chromatography and mass spectrometry were performed as described (32). The generated RAW files were initially analyzed using Byonic (ProteinMetrics). Detected modifications to asparagine residues were analyzed using the N-glycan database within Byonic to determine glycan composition. Glycopeptides with a log probability < 1 or with less than two spectra counts were excluded from the analysis. The ten most abundant glycans at each N-glycosylation site were determined from spectral counts. The top ten most abundant N-glycans from each site were manually evaluated by comparing retention time and analyzing the MS2 spectra with XCaliber Qual Browser (ThermoFisher). MS data were deposited in the MASSIVE database (http://massive.ucsd.edu) under accession MSV000087378.

### NMR spectroscopy

Mm FcγRIII Val^158^ or Ile^158^ samples were exchanged into a buffer containing 20 mM sodium phosphate, 100 mM potassium chloride, 50 µM sodium trimethylsilylpropanesulfonate, pH 7.2 with 5% ^2^H_2_O using a 10 kDa-cutoff Amicon centrifugal filtration unit (EMD Millipore) for experiments observing ^1^H-^15^N correlations. Samples for observing ^1^H-^13^C correlations were exchanged into the same buffer that was first lyophilized, then reconstituted with 100% ^2^H_2_O. ^1^H-^13^C NMR experiments were conducted on a 14.1T Oxford magnet equipped with a Bruker Avance III console and a 5 mm TCI cryoprobe. ^1^H-^15^N NMR experiments were conducted on a 21.1T Oxford magnet equipped with a Bruker Neo console plus a 5 mm TXI cryoprobe. Both spectrometers are operated by TopSpin version 3.5. Experiments were collected with a sample temperature of 25°C using either the HSQCETFPF3GPSI pulse sequence for observing ^1^H-^15^N correlations or the HSQCGP pulse sequence for observing 1H-13C correlations. Data were processed using NMRPipe (33) and plotted using NMRViewJ (http://NMRFX.org).

## Results

### Mm FcγRIII(Ile^158^) shows higher binding affinity to Mm IgGs than Mm FcγRIII(Val^158^)

In humans, polymorphism at position 158 of FcγRIIIa leads to two allelic variants, a lower affinity Phe^158^ and a higher affinity Val^158^ with an approximately 2-4 fold increase in affinity for human IgG1 (24). The affinity differences observed for the FcγRIIIa alleles in humans directly translate to their functionality, with the Phe^158^ variant having significantly lower cell mediated activity elicited by IgG1 or IgG3 Fc (13). The molecular details of the interaction of human FcγRIIIa and b with IgG1 Fc are relatively well documented through structural studies of complexes formed by the extracellular d1d2 domains of the receptor with the Fc C_H_2-C_H_3 domains. These include multiple crystal structures of FcγRIIIb or the high affinity FcγRIIIa Val^158^ variant with the Fc dimer of human IgG1 (21, 34–39). These studies define the asymmetric interaction sites within the Fc dimer that include the Fc C_H_2 domain BC, DE, and FG loops of monomer A and the Fc C_H_2 domain FG loop of monomer B with the receptor. In these structures the main chain of Val^158^ specifically interacts with the N-terminus of the C_H_2 domain of monomer B. The differences in affinity between Val^158^ and Phe^158^ are thought to be the result of the larger and bulkier aliphatic Phe side chain (7 versus 3 carbons) although the Val side chain (and presumably also the Phe side chain) does not directly interact with the Fc but instead loosely packs against Trp^90^ in the receptor. Unfortunately, there is no structure of the human lower affinity FcγRIIIa Phe^158^ variant in complex with Fc to confirm these predictions.

As in humans, Mm FcγRIII has a polymorphism at position 158, with a more abundant Ile^158^ and less abundant Val^158^ variant. However, in contrast to human, the Mm variant with Ile, a residue with an aliphatic side-chain that only differs by the addition of one methyl group as compared to Val, shows higher binding affinity to human and macaque IgGs (with K_D_s of 2-10 μM and 10-50 μM for the Ile^158^ and Val^158^ variants, respectively, in binding to polyclonal IgG purified from naive rhesus macaques (10, 23)). Both Val and Ile are classified as non-polar, aliphatic and branched amino acids, but Ile is asymmetric and has a higher hydrophobic index (40). To confirm that these affinity differences are the result of the mode of interaction between FcγRIII and the Fc portion of IgG, we re-examined the binding affinities of the Mm Ile^158^ and Val^158^ variants to Mm IgG1-4 and the Fc C_H_2-C_H_3 domains of Mm IgG1-4 (**Table 1**, top rows, **Figure S1**, **Table S1** **)**. In these initial binding studies, we used fully glycosylated receptor variants with five glycosylation sites. Proteins were produced in HEK293 cells and are expected to display complex-type sugars (e.g. addition of at least two branch N-acetylglucosamine residues). Consistent with previous published results, the Mm FcγRIII(Ile^158^) variant had generally higher affinity to Mm IgG than the Val^158^ variant; one clear exception was to macaque IgG4 where the Mm FcγRIII(Val^158^) variant had higher affinity. Also consistent with previous results, both variants had higher affinity to macaque IgG1 than to macaque IgGs 2, 3, and 4, although the differences between IgG1 and IgGs 2, 3, and 4 are fewer in macaques than in humans (9); macaque IgG subclasses lack many of the distinguishing characteristics present in human IgG subclasses such as the long hinge region in IgG3 or weak interaction with FcγRs seen for IgGs 2 and 4. This pattern was largely preserved for the binding of the receptor variants to Mm IgG Fcs. The lone exception was the Fc of IgG4, which displayed a lower affinity than whole IgG4 for the Ile^158^ variant and an almost identical affinity for the Val^158^ variant (**Tables 1** and **S1**). Interestingly, we detected the highest affinity of both receptor variants to the Fcs of Mm IgG2 with affinities to Fc following IgG2>IgG1>IgG3>IgG4. The highest affinity among whole IgGs was to IgG1 with an order of IgG1>IgG4>IgG2>IgG3. These results contrast slightly with published data obtained for the binding affinity of the Mm FcγRIII variants that had a pattern of IgG1>IgG2>IgG4>IgG3 (10, 23). Altogether these data point toward the possibility that macaque FcγRIII(Ile^158^) plays the role of the high affinity variant (equivalent to FcγRIIIa(Val^158^)) in humans, while FcγRIII(Val^158^) is more analogous to the low affinity FcγRIIIa(Phe^158^) variant. The differences in the affinity patterns observed for isolated Fcs of the Mm IgG subclasses versus intact Mm IgGs suggest that although the primary binding site of receptor is contained within the Fc portion of the antibody there are also contributions from other parts of the IgG such as the hinge region, although differences in glycosylation from the different protein preparations could also be playing a role.

**Table 1:** Binding of the allelic Mm FcγRIII Ile^158^ and Val^158^ variants to the Mm IgGs and Fcs of the Mm IgG subclasses.

	Mm IgG1 (μM)		Mm IgG2 (μM)		Mm IgG3 (μM)		Mm IgG4 (μM)	
**Mm FcγRIII variant**	Fc	IgG	Fc	IgG	Fc	IgG	Fc	IgG
**Ile^158^** fully glycosylated, complex glycans	0.25	0.26	0.23	1.8	0.40	1.9	1.8	0.98
**Val^158^** fully glycosylated, complex glycans	0.36	0.41	0.09	2.1	0.46	3.2	0.63	0.57
**Ile^158^** fully glycosylated, Man5 glycans	0.21	0.22	0.15	1.5	0.29	1.9	1.1	0.49
**Val^158^** fully glycosylated, Man5 glycans	0.30	0.28	0.08	1.8	0.24	2.3	0.53	0.30
**Ile^158^** partial* complex glycans	0.12	0.34	1.6	2.3	0.23	2.8	2.2	2.3
**Val^158^** partial* complex glycans	0.03	0.46	1.2	2.6	0.25	4.2	1.0	3.0
**Ile^158^** partial* Man5 glycans	0.06	0.28	1.5	2.8	0.16	3.0	1.9	1.7
**Val^158^** partial* Man5 glycans	0.01	0.29	0.61	2.2	0.28	4.8	0.85	2.2

*variants with Asn^38^ and Asn^169^ glycans removed

Equilibrium binding constants (K_D_) were measured by SPR. Data are average of two independent experiments. Color scheme used: green (higher affinity) to red (lower affinity) within each column.

### Signaling through either the Mm FcγRIII(Ile^158^) or Mm FcγRIII(Val^158^) allotype can impact the level of antibody dependent cellular cytotoxicity

**Figure 1:**
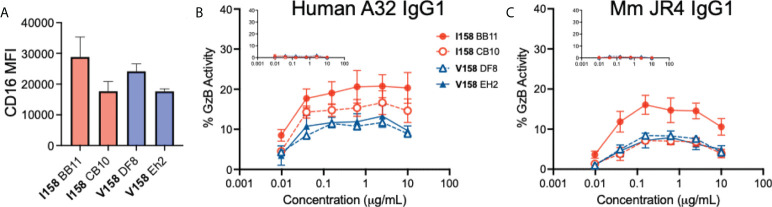
Antibody-dependent cell-mediated cytotoxicity activities (ADCC) of NK-92 clones transduced to express *Macaca mulatta* (Mm) *FCGR3A* with SNPs at position 158. **(A)** Median fluorescent intensity (MFI) of cell surface CD16 (FcγRIII) on NK-92 clones with Ile^158^ (n=2, red) and Val^158^ (n=2, blue) SNPs. ADCC activity of **(B)** human and **(C)** Mm IgG1 specific for the HIV-1 Env cluster A region (monoclonal antibodies A32 and JR4, respectively) in assays performed with NK-92 clones expressing the Ile^158^ (red lines) and Val^158^ (blue lines) variants of FcγRIII. Inset graphs represents activity observed with an influenza specific negative control antibody, CH65, produced as human and Mm IgG1. Data represents mean and standard error from four independent experiments.

In order to see if the difference in affinity between the Mm FcγRIII(Ile^158^) and FcγRIII(Val^158^) variants for IgGs translated to a difference in functional activity, we developed an antibody-dependent cell-mediated cytotoxicity (ADCC) assay in which NK-92 clones were transduced to express Mm *FCGR3A* with Ile^158^ or Val^158^ encoding SNPs at position 158 could be used as effector cells. ADCC was measured by detection of active Granzyme B in target cells, as previously described (26). To begin, we compared levels of FcγRIII expression on the surface of our NK-92 cells using an anti-CD16 antibody by flow cytometry. As show in **Figure 1A**, we identified a high and low expressing NK-92 clone for both FcγRIII(Ile^158^) and FcγRIII(Val^158^). Next, we used these clonal lines as effector cells in ADCC experiments performed with two mAbs (JR4 and A32) that recognize Cluster A epitopes (41). JR4 was originally isolated from a SHIV infected Mm expressed as Mm IgG1 and A32 is a human antibody isolated from an HIV-1 infected individual recombinantly expressed as human IgG1. The CEM.NKR_CCR5_ cell line (28) was used as target cells after being coated with recombinant HIV subtype B BaL isolate gp120. We first evaluated ADCC using the human A32 IgG1 antibody. As shown in **Figure 1B**, we observed slightly higher ADCC (higher frequency of target cells with granzyme B activity) when NK-92 cells expressing Mm FcγRIII(Ile^158^) were used as effector cells. When assays were performed with the JR4 Mm IgG1 mAb (**Figure 1C**), there were no differences between ADCC activity among the** **low-expressing FcγRIII(Ile^158^) and FcγRIII(Val^158^) NK-92 clones, yet greater ADCC was observed when the high-expressing NK-92 Ile^158^ clone BB11 was used as effector cells. The lack of observed difference between the low expressing clones may be due to the limited resolution of the assay in this low response range, or may suggest that other factors, including the abundance of receptor on the cell surface, can also impact the functional activity of Mm FcγR. As expected, no ADCC activity was observed for assays performed with an influenza-specific negative control antibody, recombinantly produced as human IgG1 (**Figure 1B** inset) or as Mm IgG1 (**Figure 1C** inset). Overall, these data suggest that the Ile^158^ variant may have the ability to mediate higher levels of ADCC. The Mm FcγRIII–Fc(IgG1) complex closely resembles both the human FcγRIIIa-Fc(IgG1) and FcγRIIIb-Fc(IgG1) complexes.

**Figure 2:**
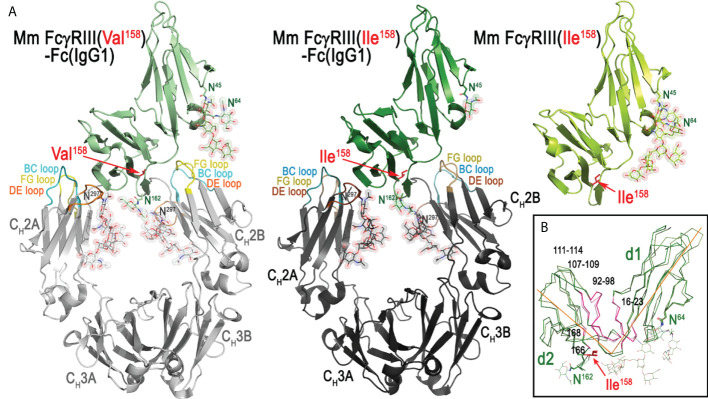
The structures of Rhesus macaque, *Macaca mulatta* (Mm), FcγRIII(Val^158^)-Fc(IgG1), FcγRIII(Ile^158^)-Fc(IgG1), and FcγRIII(Ile^158^) (apo). **(A)** Structures are shown in a ribbon diagram with the three macaque FcγRIII receptors in light green (FcγRIII-Val^158^, top left structure), dark green (FcγRIII-Ile^158^, middle structure), and lime (apo Mm FcγRIII-Ile^158^, top right structure), and the two heavy chains (C_H_2-C_H_3 domains) in light gray (Mm FcγRIII(Val^158^)-Fc) and dark gray (Mm FcγRIII(Ile^158^)-Fc). Sugars attached to Asn^297^, Asn^162^, Asn^45^ and Asn^64^ are shown as spheres colored by atom type (backbone color for carbon, red for oxygen and blue for nitrogen). The BC, DE, and FG loops of C_H_2 domain in FcγRIII(Val^158^)-Fc(IgG1) and FcγRIII(Ile^158^)-Fc(IgG1) structures are colored in lighter and darker shades of cyan, yellow, and orange, respectively. The two heavy chain monomers are labeled as A and B chains. **(B)** Superimposition of Mm FcγRIII(Ile^158^) receptor from FcγRIII(Ile^158^)-Fc(IgG1) complex structure onto the apo Mm FcγRIII(Ile^158^) to show conformational differences. Only main chain atoms are shown as ribbons with colors for the receptor as in panel **(A)** Ile^158^ and glycosylation site residues (Asn^45^, Asn^64^, and Asn^162^) are shown as sticks.

In order to understand molecular details of the interaction of Mm FcγRIII allotypes with Mm IgGs and to dissect differences in the overall architecture of the FcγRIII-Fc complexes that could explain the differences in binding affinities, we solved the crystal structures of the Ile^158^ and Val^158^ variants in complex with the Fc of Mm IgG1. We also determined the high-resolution structure of the Mm FcγRIII(Ile^158^) variant alone (apo), not complexed with Fc. In structural studies, we used receptor variants in which glycosylation sites at Asn^38^ and Asn^169^ (two of the five total) were removed by mutagenesis to aid in crystallization. The glycans on Asn^38^ and Asn^169^ match those present on human FcγRIIIa that are not involved in binding Fc(IgG1) (42). Two receptor complexes with the Fc of IgG1 were solved. The first utilized the Ile^158^ variant expressed in a GnT1- cell line after deglycosylation with EndoH_f_ to trim glycans back to a single Asn-linked N-acetylglucosamine (GlcNAc) residue at glycosylation sites: Asn^45^, Asn^64^, and Asn^162^. The second complex was formed using the Val^158^ variant expressed in a GnT1- cell line without deglycosylation which leaves the Man5 N-glycans that consist of seven carbohydrate residues from the GnT1- expression at glycosylation sites. The apo macaque FcγRIII(Ile^158^) receptor, expressed as Val^158^ variant, was crystallized in orthorhombic space group P2_1_2_1_2_1_ with cell dimensions a = 49.1 Å, b = 67.0 Å, and c = 69.7 Å. Crystals diffracted to 1.9 Å resolution with one receptor in the asymmetric unit. The model was refined to an R/R_free_ of 0.182/0.218 (**Figure 2**). Both the Mm FcγRIII Ile^158^ and Val^158^ receptor variants in complex with Mm IgG1 Fc crystallized in monoclinic space group C2 with similar cell dimensions. Both complex crystals contained two FcγRIII-Fc complexes in the asymmetric unit, diffracted to 3.8 Å and 3.15 Å, and were refined to an R/R_free_ of 0.276/0.324 and 0.269/0.303, respectively (**Figure 2** and **Figure S2**). Complete data collection and refinement statistics are found in **Table 2**.

**Table 2:** Data collection and refinement statistics.

	Mm FcγRIII (Ile^158^)/Fc (IgG1) complex	Mm FcγRIII (Val^158^)/Fc (IgG1) complex	Mm FcγRIII (Ile^158^)
**Data collection**		
Wavelength, Å	0.979	0.979	0.979
Space group	C2	C2	P2_1_2_1_2_1_
Cell parameters			
a, b, c, Å	213.1, 71.3, 135.5	215.7, 70.1, 135.4	49.1, 64.0, 70.0
α, β, γ, °	90,119.7, 90	90,119.8, 90	90, 90, 90
Complexes/a.u.	2	2	1
Resolution, (Å)	50-3.8 (4.0-3.8)	50-3.15 (3.32-3.15)	50-1.9 (1.93-1.9)
# of reflections			
Total	33,764	93,482	105,044
Unique	14,678	29,529	17,804
R_merge_ ^a^, %	18.4 (63.8)	15.0 (91.0)	15.3 (70.4)
R_pim_ ^b^, %	14.5 (49.6)	10.1 (60.5)	6.7 (43.5)
*CC_1/2_* ^c^	0.96 (0.75)	0.99 (0.70)	0.99 (0.77)
I/σ	3.4 (1.0)	3.0 (0.8)	27.4 (1.4)
Completeness, %	83.9 (86.9)	96.4 (96.4)	99.2 (96.0)
Redundancy	2.3 (2.3)	3.2 (3.1)	5.9 (4.7)

**Refinement Statistics**			
Resolution, Å	50.0 – 3.8	50.0 – 3.15	50.0 – 1.9
R^d^ %	27.6	26.9	18.2
R_free_ ^e^, %	32.4	30.3	21.8
# of atoms			
Protein	9,418	9,468	1,398
Water	–	8	87
Ligand/Ion	438	551	112
Overall B value (Å)^2^			
Protein	96	77	44
Water	–	49	46
Ligand/Ion	116	80	55
RMSD^f^			
Bond lengths, Å	0.006	0.010	0.015
Bond angles, °	1.1	1.5	1.6
Ramachandran^g^			
favored, %	75.3	75.3	97.6
allowed, %	16.6	16.8	2.4
outliers, %	8.1	7.9	0.0
PDB ID	6MJ3	7KCZ	6MJO

Values in parentheses are for highest-resolution shell.

a R_merge_ = ∑│I - <I>│/∑I, where I is the observed intensity and <I> is the average intensity obtained from multiple observations of symmetry-related reflections after rejections

b R_pim_ = as defined in (43).

c CC_1/2_ = as defined by Karplus and Diederichs (44).

d R = ∑║F_o_│- │ F_c_║/∑│F_o_ │, where F_o_ and F_c_ are the observed and calculated structure factors, respectively.

e R_free_ = as defined by Brünger (45).

f RMSD = Root mean square deviation.

g Calculated with MolProbity.

As shown in **Figure 2**, similar to human low affinity FcγR receptors, the macaque FcγRIII receptor consists of two immunoglobulin-like domains packed against one another at a roughly 80-90 degree angle in a comma- or apostrophe-like shape. The average angle between domains for the macaque FcγRIII is 82.7 degrees (for five FcγRIII receptors in the three crystal structures, i.e. two copies each from two FcγRIII complex structures and one apo structure) compared to 86.4 degrees for the human FcγRIIIa (PDB IDs 3AY4, 3SGJ, 3SGK, 5VU0, and 5XJE) (calculated between the average α-carbon positions of the shared residues between structures in domains 1 and 2 with the α-carbon position of Gly^89^ as the origin). This angle is maintained by stabilizing contacts between residues 16-23, and 87 on domain 1 and 92-98, 107-109, 111-114, and 168 on domain 2. In addition, there is a hydrogen bond between the Asn^45^ linked glycan mannose on domain 1 and Glu^166^ on domain 2 which is only visible in the apo receptor structure due to its higher resolution and better resolved glycan density. This added hydrogen bond may provide an explanation for the detrimental effect of removing this glycan on expression (37) (**Figure 2**). From the glycosylation sites left unchanged on Mm variants (Asn^45^, Asn^64^, Asn^162^), two (Asn^45^ and Asn^162^) are found on human FcγRIIIa. The glycosyl group attached to Asn^45^ was shown to be important for expression of human FcγRIIIa and Asn^162^ plays a role in FcγRIIIa’s higher affinity to afucosylated Fcs (37, 39). While one well-ordered glycan can be seen attached to Asn^45^ in the apo macaque receptor structure, no glycan is visible linked to Asn^162^ due to disorder. The two macaque receptor-Fc(IgG1) complexes have a visible glycan attached to Asn^162^, which may be a result of the stabilizing effect of the Fc or differences in crystal packing of the receptor in the two space groups; the GlcNAc attached to Asn^162^ is also stabilized by Arg^155^ in the macaque receptor similar to what is seen in the human complexes, but this stabilizing effect is only evident in complexes with the Fc of IgG1. There is also density for a GlcNAc linked to Asn^64^. Human FcγRIIIa has an aspartic acid at this position and is not glycosylated, while the human FcγRIIIb NA2 and SH2 alleles have an Asn and are also potentially glycosylated, but the degree of glycosylation may be dependent upon glycosyl enzyme access in the Endoplasmic reticulum (ER) and Golgi (20, 46, 47).

The amino acid sequence of the extracellular domains of Mm FcγRIII differ by 11 and 13 residues to human FcγRIIIa and FcγRIIIb, respectively. Although Mm FcγRIII and human FcγRIIIa are closest in sequence identity, Mm FcγRIII shares a potential glycosylation site at position 64 with human FcγRIIIb (with five total glycosylation sites in Mm FcγRIII: Asn^38^, Asn^45^, Asn^64^, Asn^162^, and Asn^169^, as compared to a possible six in human FcγRIIIb depending upon the allotype: Asn^38^, Asn^45^, Asn^64^, Asn^74^, Asn^162^, and Asn^169^). In order to determine if these differences in receptor sequence translate into changes to their Fc(IgG1) complex structures, we compared both Mm FcγRIII-Fc(IgG1) complexes to the available structures of human FcγRIIIa and FcγRIIIb in complex with Fc(IgG1) in the Protein Data Bank (PDB) (**Table 3**). We limited our comparison to complexes with fucosylated Fcs (similar to the fucosylated Mm Fc(IgG1) in our studies), with the exception of the FcγRIIIb complex of 6EAQ, which was afucosylated (PDB IDs: 3SGJ, 5XJE,1E4K, 1T83, and 6EAQ). As shown in **Table 3**, the average main chain Root-Mean-Square Deviation (RMSD) between the four copies of the two macaque variant structures is 1.11 Å. When the Mm complexes are compared to the human FcγRIIIa and FcγRIIIb complexes above, they are both more similar to the FcγRIIIb complex (PDB: 6EAQ) (with an average main chain RMSD of 1.14 Å to the two copies of the macaque Val^158^ complex and 1.30 Å to the two copies of the Ile^158^ complex). They are more akin to human FcγRIIIb despite the fact that the sequence of macaque FcγRIII is more similar to human FcγRIIIa. The closest human FcγRIIIa structure to both is the 5XJE complex with an average main chain RMSD of 1.35 Å and 1.5 Å to the macaque Val^158^ and Ile^159^ complexes, respectively. The FcγRIIIa (5XJE) structure is more analogous to the macaque structures than the other human FcγRIIIb structures, potentially due to the fact that they were produced in *Escherichia coli* and lack glycans; 6EAQ is the only available human FcγRIIIb complex with a partially glycosylated receptor. Overall, these data indicate that structures of complexes formed by the extracellular domain of Mm FcγRIII(Ile^158^) or FcγRIII(Val^158^) with the Fc of Mm IgG1 closely resemble structures of the human FcγRIIIa-Fc(IgG1) and FcγRIIIb-Fc(IgG1) complexes with the closest similarity to the latter.

**Table 3:** Structural comparison of the macaque and human FcγRIII-Fc(IgG1) complexes.

FcγR-Fc(C_H_2) complex												
**FcγR-Fc(C_H_2-C_H_3) complex**	Mm III(V^158^), a	Mm III(V^158^), a	1.10	1.10	0. 98	1.18	1.08	2.19	1.50	2.93	1.88	1.33
	Mm III(V^158^), b	1.06	Mm III(V^158^), b	1.10	1.35	1.21	1.28	2.07	1.22	2.71	1.67	1.13
	Mm III(V^158^), avg	1.06	1.06	Mm III(V^158^), avg	1.16	1.20	1.18	2.13	1.36	2.82	1.78	1.23
	Mm III(I^158^), a	0.96	1.25	1.11	Mm III(I^158^), a	1.23	1.23	2.45	1.72	3. 27	>2.14	1.53
	Mm III(I^158^), b	1.11	1.12	1.12	1.17	Mm III(I^158^), b	1.23	2.14	1.47	2.99	1.81	1.34
	Mm III(I^158^), avg	1.04	1.19	1.11	1.17	1.17	Mm III(I^158^), avg	2.30	1.59	3.13	1.97	1.43
	Hs IIIa(V^158^), 3sgj	1.91	1.82	1.86	2.19	1.89	2.04	Hs IIIa(V^158^), 3sgj	1.65	2.49	1.62	1.61
	Hs IIIa(V^158^), 5xje	1.47	1.22	1.35	1.64	1.40	1.52	1.52	Hs IIIa(V^158^), 5xje	2.04	1.07	0.73
	Hs IIIb(V^158^), 1e4k	3.15	2. 91	3.03	3.41	3.09	3.25	2. 65	2.14	Hs IIIb(V^158^), 1e4k	2.24	2.35
	Hs IIIb(V^158^), 1t83	1.73	1.53	1.63	1.95	1.64	1.79	1.47	1.02	2.29	HS IIIb(V^158^), 1t83	1.18
	Hs IIIb(V^158^), 6eaq	1.22	1.07	1.14	1.36	>1.24	1.30	1.51	0.89	2.74	1.19	HS IIIb(V^158^), 6eaq

Average RMSD values for main chain atoms for pairwise comparisons of available FcγRIII-Fc(C_H_2-C_H_3) (left) and FcγRIII-Fc(C_H_2) complexes.

### The Mm FcγRIII(Ile^158^)-Fc(IgG1) and Mm FcγRIII(Val^158^)-Fc(IgG1) complexes are formed with a smaller contribution from the receptor glycan to the interface as compared to their human counterparts

**Figure 3:**
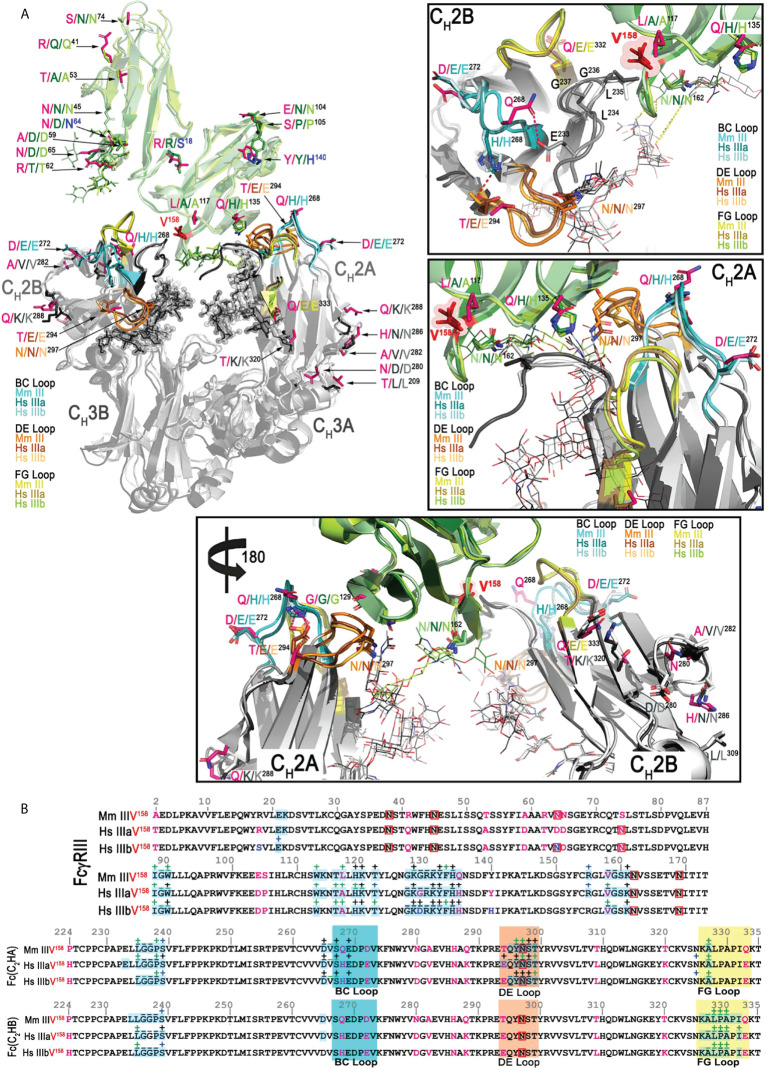
Structural comparison of the macaque FcγRIII(Val^158^)-Fc(IgG1) complex with the two human FcγRIIIa/b(Val^158^)-Fc(IgG1) complexes (PDB: 5XJE and 6EAQ, respectively). **(A)** Superimposition of the structures and the interfaces of the Mm FcγRIII(Val^158^)-Fc(IgG1) and the two Hs FcγRIIIa/b(Val^158^)-Fc(IgG1) complexes shown in a ribbon diagram with colors indicated as in **Figure 1**. The three panels on the right and bottom are the blow up views of the interfaces of the complexes. The two Fc monomers are labeled as A and B chains. Residues of Mm FcγRIII-Fc complex that differ from the Hs complexes are colored in magenta. Residues of the two Hs FcγRIIIa/b-Fc(IgG1) complexes are shown in lighter and darker color shades, respectively. Residues in the Hs FcγRIIIb-Fc(IgG1) complex that differ from the Hs FcγRIIIa-Fc(IgG1) complex are highlighted in blue. The BC, DE and FG loops of the Mm FcγRIII-Fc complex are colored in cyan, orange and yellow, respectively. BC, DE, FG loops of the two Hs FcγRIII-Fc(IgG1) complexes (Hs FcγIIIaVal^158^ and Hs FcγIIIbVal^158^) are colored in darker and lighter shades of cyan, orange and yellow (in respect to the colors of the Mm FcγRIII-Fc(IgG1) complex), respectively. The same color scheme is applied to the remaining panels. **(B)** Pairwise sequence alignment of the FcγRIII receptors (top panel) and Fc(IgG1) (bottom panel) in the Mm FcγRIII(Val^158^)-Fc and the two Hs FcγRIIIa/b(Val^158^)-Fc(IgG1) complexes. Glycosylated residues are in red boxes. Buried surface residues are determined by PISA and are shaded in blue. Contact residues defined by a 5 Å cutoff are marked above the sequence with (+) for side chain and (-) for main chain to indicate the type of contact. Contact types are colored as follows: hydrophilic (green), hydrophobic (blue) and both (black).

**Figure 4:**
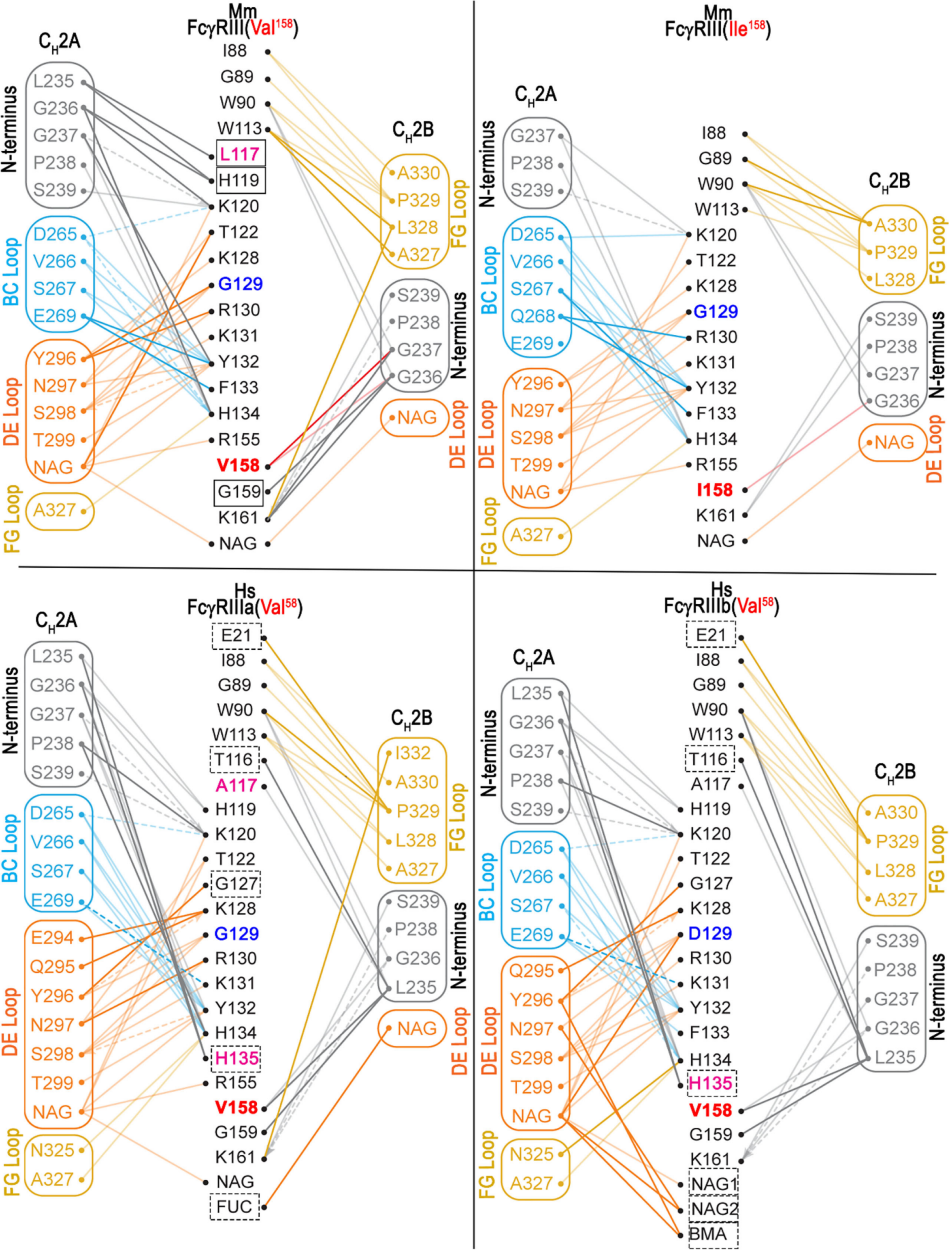
Networks of interactions formed between FcγRIII receptors and the two Fc monomers (C_H_2-C_H_3 domains). The two Fc monomers are labeled as A and B chains. The residues in the N-terminus and the BC, DE, FG loops are colored in gray, cyan, orange and yellow, respectively. The top two panels show the networks of interactions between the two Mm FcγRIII-Fc(IgG1) complexes and the bottom two panels show the networks of interactions between the two Hs FcγRIIIa/b-Fc(IgG1) complexes (PDB: 5XJE and 6EAQ). Networks of interactions defined by a 5-Å distance criterion cutoff are shown as dotted lines for main chain-main chain interactions and dashed lines for main chain to side chain interactions with the arrows indicating the residues that contribute to the side chain interactions. Darker versus lighter color lines for the Hs FcγRIIIa/b-Fc(IgG1) complexes and the Mm FcγRIII(Ile^158^)-Fc(IgG1) complex represent those contacts that are unique as compared to the Mm FcγRIII(Val^158^)-Fc(IgG1) complex. Darker versus lighter color lines for the FcγRIII(Val^158^)-Fc(IgG1) complex are those that are unique relative to the Mm FcγRIII(Ile^158^)-Fc(IgG1) complex. Lighter color lines represent those that are common between the comparisons. Additional residues that contribute to the buried surface area (BSA) of the Mm FcγRIII(Val^158^)-Fc(IgG1) complex relative to the Mm FcγRIII(Ile^158^)-Fc(IgG1) are indicated with boxes. Additional residues that contribute to the BSA difference between the two Hs FcγRIIIa/b-Fc(IgG1) complexes relative to the Mm FcγRIII(Val^158^)-Fc complex are indicated with dashed boxes. Residues that are different between the Mm FcγRIII-Fc(IgG1) complexes and the Hs FcγRIIIa/b-Fc(IgG1) complexes are colored in magenta. Residues that are unique to FcγRIIIb(Val^158^)-Fc(IgG1) complex are colored in blue.

Amino acid sequence differences between Mm FcγRIII and human FcγRIIIa and b occur mostly outside the areas that bind to the Fc dimer; the extracellular portion of the macaque FcγRIII used for crystallization is 93.2% identical to human FcγRIIIa and 92.1% identical to human FcγRIIIb. A similar level of sequence difference is seen for the macaque Fc, which is 92.4% identical in sequence to the human Fc. **Figure 3A** shows the side chains of residues that differ between Mm and human, magenta for human FcγRIIIa and Fc and blue for FcγRIIIb. Since all reported structures of human FcγRIIIa and FcγRIIIb have Val^158^, comparisons were performed for the Mm FcγRIII(Val^158^) only. **Figure 3B** shows contact residues for the receptor and Fc at the interface for the Mm FcγRIII(Val^158^)-Fc(IgG1) complex as compared to those from the most similar structures for the human FcγRIIIa(Val^158^)-Fc(IgG) and FcγRIIIb(Val^158^)-Fc(IgG) complexes, i.e. 5XJE and 6EAQ respectively. As mentioned previously, one key difference between the macaque FcγRIII and human FcγRIIIa and b receptors is glycosylation. An Asn to Ser change at position 74 removes the glycosylation site present in both human FcγRIIIa and FcγRIIIb and an Asp to Asn change at position 64 adds a glycosylation site was not present in human FcγRIIIa but potentially present in the NA2 and SH alleles of human FcγRIIIb; the FcγRIIIb in 6EAQ has this site mutated to Gln to prevent glycosylation so an equivalent glycan was not seen in the structure. The main difference in sequence at the Fc binding interface in the macaque FcγRIII is Leu^117^ which is alanine in both human copies of the gene. **Figure 4** shows the interaction network formed at the receptor-Fc interface of the Mm and both human complexes. Leu^117^ makes van der Waals contact with Leu^235^ of chain A of the Fc in the Mm FcγRIII(Val^158^)-Fc(IgG1) complex. In contrast, Ala^117^ in both the human FcγRIIIa and FcγRIIIb complexes makes a similar van der Waals contact to Leu^235^ of chain B of the Fc. The smaller side chain in the human receptors may permit a closer association to the N-terminus of chain B in the human complexes. Both human structures have significantly higher buried surface area (BSA) to the N-terminus of the Fc of chain B than the macaque structure; the BSA to the N-terminus of the Fc of chain A is largely comparable between the three structures (**Table S2**). The only other sequence difference in the binding region is at position 135 which is His in both human FcγRIIIs and Gln in the Mm FcγRIII. His^135^ in human FcγRIIIa and FcγRIIIb makes both van der Waals contact with Leu^235^ and a hydrogen bond to Gly^236^ in the Fc of chain A, while Gln^135^ in the macaque complex does not make any contacts to the Fc due to differences in conformation of the Fc N-termini (**Figure 4**). Overall, the Mm FcγRIII(Val^158^)-Fc(IgG1) complex is formed with a BSA of 1,832 Å^2^ which is 10 and 17% lower than BSA of the human FcγRIIIa(Val^158^)-Fc(IgG1) (BSA of 2,041 Å^2^) and FcγRIIIb(Val^158^)-Fc(IgG1) (BSA of 2,207 Å^2^) complexes, respectively.

Although there are four sequence differences relative to human in the macaque Fc BC, DE, and FG loops, none are in residues directly involved in binding to the receptor. Instead, the main difference appears to be conformational in nature. The human IgG1 BC and DE loops are stabilized by a hydrogen bond between BC loop His^268^ and DE loop Glu^294^ which may keep the two loops in a conformation that facilitates chain A’s binding to the receptor. Gln^268^ and Thr^294^ in the macaque Fc can also form a hydrogen bond, but the bond is weaker and shorter due to charge and distance constraints (**Figure 3A**, blow-up view panels). It is therefore not surprising that this residue pair has multiple conformations in the four copies in the asymmetric unit of the two Mm complex structures. This includes one in which Gln^268^ is involved in a H-bond to Glu^233^ of the C_H_2B N-terminus which may help hold the N-terminus in a conformation that places Leu^234^ and Leu^235^ in a good orientation for interacting with the residue at position 158. Stabilization of the DE loop also helps stabilize the Fc glycan linked to Asn^297^, which also contributes to complex formation. The Fc of chain A packs against the glycan on Asn^162^ in the receptor in both the human and macaque structures. In the human FcγRIIIb complex, the lack of a fucosyl group on the Fc leads to a more extensive contact area. Overall, the Mm complexes are formed with a smaller contribution to the BSA from glycan-glycan or glycan-protein contacts between the Fc and the receptor: 204 and 164 Å^2^ in the Mm FcγRIII(Val^158^)-Fc(IgG1) and FcγRIII(Ile^158^)-Fc(IgG) complexes, respectively, compared to 228 and 487 Å^2^ in the human FcγRIIIa (Val^158^)-Fc(IgG1) and FcγRIIIb (Val^158^)-Fc(IgG) complexes, respectively.

### The Mm FcγRIII(Val^158^)-Fc(IgG1) complex has a higher buried surface area and a more extensive receptor glycan-Fc glycan interface than the Mm FcγRIII(Ile^158^)-Fc(IgG1) complex

**Figure 5:**
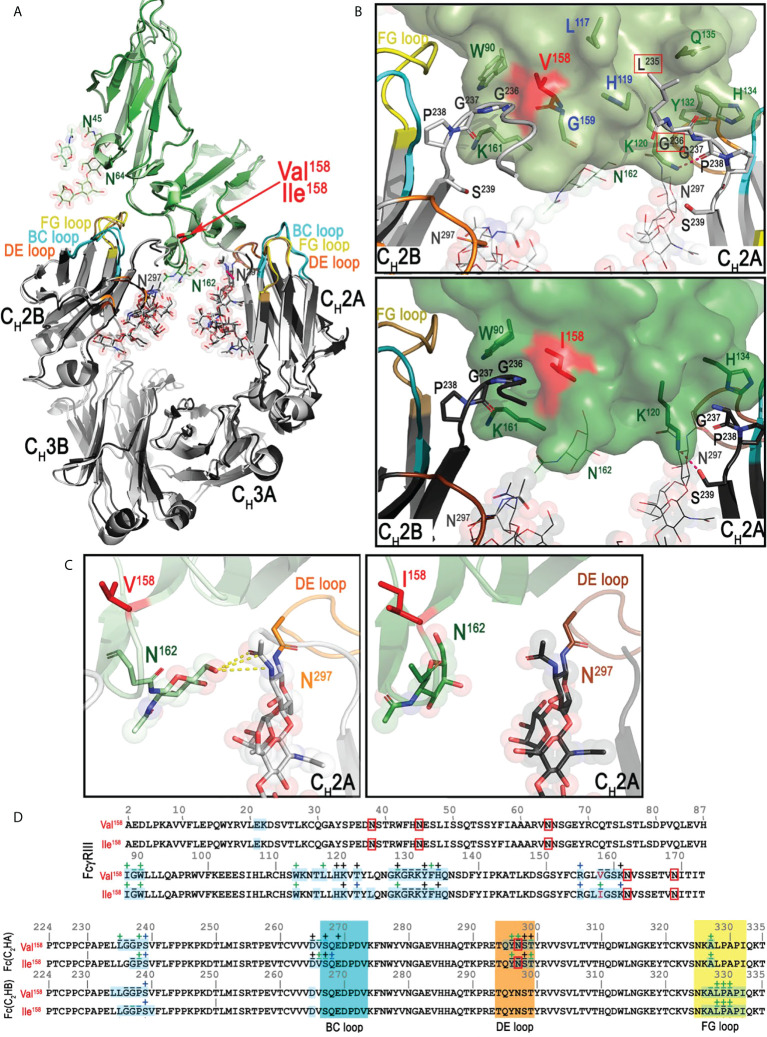
Structural comparison of the Mm FcγRIII(Val^158^)-Fc(IgG1) and Mm FcγRIII(Ile^158^)-Fc(IgG1) complexes. **(A)** Superimposition of the two Mm FcγRIII-Fc(IgG1) structures shown in a ribbon diagram with colors indicated as in panel A of **Figure 1**. The two heavy chain monomers are labeled as A and B chains. The interfaces **(B)** and the blow up views of the hydrogen bond networks **(C)** of the interactions between Mm IgG1-Fc with Mm FcγRIII(Val^158^) (top panel **B**, left panel **C**) and Mm FcγRIII(Ile^158^) (bottom panel **B**, right panel **C**) with colors as in panel **(A)** The Mm FcγRIII receptors are shown as a molecular surface. The contact residues of IgG1-Fc and FcγRIII receptors are shown as sticks. Additional residues in the Mm FcγRIII(Val^158^)-Fc(IgG1) complex for FcγRIII(Val^158^) and Fc(IgG1) that contribute to the binding are colored in blue or are indicated with red boxes, respectively. **(D)** Pairwise sequence alignment of the two Mm FcγRIII receptors (top panel) and the two Mm IgG1-Fc (bottom panel). The glycosylation residues are in red boxes. The BC, DE, and FG loops are shaded in cyan, orange and yellow, respectively. Buried surface residues are determined by PISA and shaded with blue. Contact residues defined by a 5 Å cutoff are marked above the sequence with (+) for side chain and (-) for main chain to indicate the type of contact. Contact types are colored as follows: hydrophilic (green), hydrophobic (blue) and both (black).

The Mm FcγRIII forms an asymmetric complex with macaque IgG1 Fc with the Fc binding near the bend in the comma-shaped receptor; the majority of the receptor’s contact residues to the Fc reside on the C-terminal second immunoglobulin-like (d2) domain closer to the cell membrane (**Figure 2**). In both complexes, Fc monomers bind using only their C_H_2 domains. The average total BSA of the complex formed by FcγRIII(Val^158^) is higher by 334 Å^2^ than the FcγRIII(Ile^158^) complex (an average total BSA of 1,832 Å^2^ and 1,498 Å^2^ for the Mm FcγRIII(Val^158^)-Fc(IgG1) and Mm FcγRIII(Ile^158^)-Fc(IgG1) complexes, respectively, **Table S2**). The BSA differences are the result of the decreased number of contact residues between FcγRIII(Ile^158^) and the Fc, which include direct protein-protein interactions, glycan-protein interactions, and glycan-glycan interactions (**Figures 4**, **5** and **Table S2**). The N-terminal residues of the Fc adjacent to the hinge region [residues 220-230] contribute approximately 29 and 24% of the total binding surface as measured by the buried surface area (BSA) for the Val^158^ and Ile^158^ complexes, respectively. One monomer, defined as A for all subsequent comparisons, adds approximately 46 and 45% of the BSA to the interface (Val^158^ and Ile^158^ complexes, respectively) mainly through interactions at or near the C_H_2 BC loop [residues 265-270, the BC loop spans residues 267-273] and the C_H_2 DE loop [residues 294-299] (approximately 34 and 33% of the total BSA for both complexes respectively). **Figures 5** and **S2** show the details of the binding interface for the FcγRIII(Val^158^) and FcγRIII(Ile^158^) complexes. In both cases, monomer A interacting residues form one patch on the receptor [residues 117, 119, 120, 122, 124, and 128-134 (contacts to residues 117 and 119 are absent in the Ile^158^ complex), **Figures 3A, B** and **Figure S3**]. The other monomer, defined as B for this analysis, contributes the remaining 25 and 30% of the BSA respectively to the interface almost exclusively through the C_H_2 FG loop [residues 325-332]. FcγRIII monomer B contact residues are more spread out along the receptor [residues 88-90, 113, 158-159, and 161 (contact to residue 159 is absent in the Ile^158^ complex, **Figures 3A, B** and **Figure S3**)]. With the exception of residues at the Fc N-terminus, the interaction with receptor centers on these three Fc C_H_2 loops. Monomer A uses the BC and DE loops and monomer B the FG loop to grip the receptor from either side.

The combined glycan contribution to the BSA from both Fc monomers is 11 and 10.9% for the Val^158^ and Ile^158^ complexes respectively (**Table S2**). Direct interactions between the receptor and the Fc glycan on Asn^297^ in monomer A are mainly limited to receptor residues Lys^120^, Tyr^132^, and Arg^155^ in both complexes with an additional small contribution from Thr^122^ in the Val^158^ complex. In addition, the first GlcNAc linked to Asn^162^ in the receptor also interacts with the Fc glycan on monomer A in both complexes. The glycan-glycan interaction is primarily to the Fc of monomer A in the first complex of the asymmetric unit for both variants with closer contacts observed for the FcγRIII(Val^158^) variant (**Figure 5D**) and to the glycan on the Fc of monomer B for the second complex in the asymmetric unit of the Val^158^ structure; the glycan-glycan interaction is absent in the second complex in the asymmetric unit of the Ile^158^ structure where the glycan on Asn^162^ is disordered. In summary, the FcγRIII-Fc interface of the Val^158^ variant buries more total surface area than the Ile^158^ variant and it is strengthened by glycan-glycan interactions. The direct receptor glycan-Fc glycan cross talk is much weaker in the interface of FcγRIII(Ile^158^)-Fc complex and it is absent from one of the two copies of the complex in the asymmetric unit of the crystal.

### The N-glycan composition of HEK293-expressed Mm FcγRIII(Val^158^) and Mm FcγRIII(Ile^158^) variants are highly similar

Structural studies revealed differences between the interfaces of the FcγRIII-Fc complex formed by Ile^158^ and Val^158^. The latter has a higher interface BSA with added contributions from receptor residues Val^158^, Leu^117^, His^119^, and Gly^159^ [i.e. to Fc residues Leu^235^, Gly^236^ and Gly^237^ (**Figure 4**)]. The contribution to the interface from glycan-glycan or glycan-protein interactions between the Fc and the receptor is also greater in the Val^158^ complex, 204 Å^2^ as compared to 164 Å^2^ for the Ile^158^ complex. These differences can in part be explained by the difference in the resolution of the two crystallographic structures, 3.15 Å and 3.8 Å for the Mm FcγRIII(Val^158^)-Fc(IgG1) and Mm FcγRIII(Ile^158^)-Fc(IgG1) complexes respectively, but also by differences in glycosylation status of the receptor. The glycans on FcγRIII(Ile^158^) were removed by EndoH_f_, leaving only a GlcNAc on unmutated glycosylation sites (Asn^45^, Asn^64^, and Asn^162^), while FcγRIII(Val^158^) had a Man5 N-glycan at corresponding positions in its sequence. The differences we observed prompted us to examine the contribution of the glycan to the binding affinity and interface more closely.

Only one receptor glycan is directly involved at the interface with the Fc, specifically the glycan attached to Asn^162^ (with a BSA of 95 Å^2^ and 49 Å^2^ in the Mm FcγRIII(Val^158^)-Fc(IgG1) and Mm FcγRIII(Ile^158^)-Fc(IgG1) complexes respectively, **Table S2**). We determined the N-glycan composition at each site for both variants expressed in HEK293 cells. The distribution of N-glycan compositions at every site reveals information regarding the accessibility of each individual N-glycan to the processing machinery in the ER and Golgi. Although many different factors influence processing, the types of individual N-glycans present on recombinant proteins are believed to reflect their mobility. Highly mobile N-glycans are thought to receive the greatest amount of processing which leads to complex-types with high levels of branching and terminal sialylation, while those with limited mobility leads to oligomannose forms (48–50). We determined the composition for the three Mm FcγRIII N-glycans (Asn^45^, Asn^64^, and Asn^162^) using a glycoproteomic approach that preserves glycan-asparagine bonds following enzymatic proteolysis. LC-MS was used to separate and determine the mass of each glycopeptide, and an ion-selective MS dimension to fragment the species to produce a unique pattern which was used for identification.

**Figure 6:**
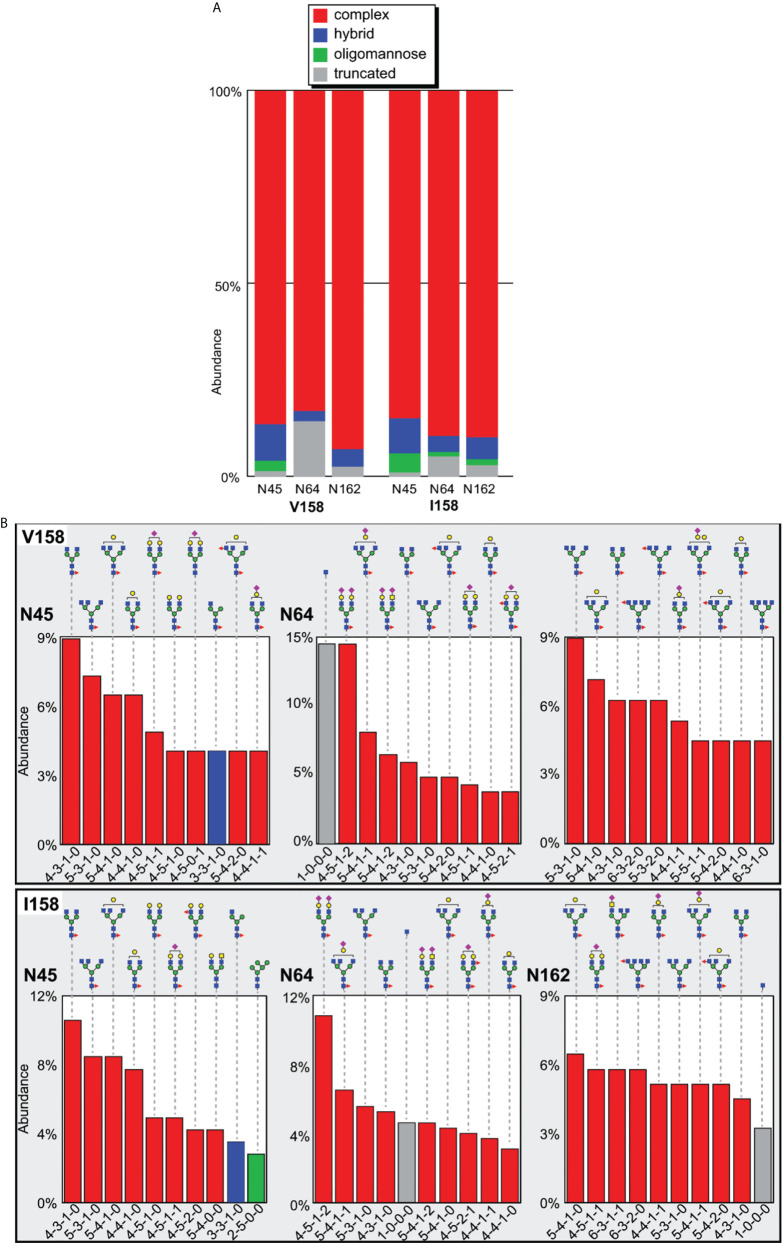
Glycan composition of Mm FcγRIII Val^158^ and Ile^158^. **(A)** Glycoforms identified at each site. **(B)** The top ten most abundant N-glycans at each site. Cartoon diagrams represent one possible configuration and utilize the SNFG convention for monosaccharides (51); isobaric ions were not distinguished. The composition of each N-glycan is provided below the chart with numbers of N-acetylhexosamine, hexose, deoxyhexose, and N-acetylneuraminic acid residues, respectively.

The N-glycosylation of the Val^158^ and Ile^158^ proteins proved highly comparable, with no remarkable differences apparent from the MS data (**Supplemental MS Excel worksheet**). The majority (>83%) of the N-glycan species observed at each site of both Mm FcγRIII allotypes were complex-type (**Figure 6A**). Notably, the Asn^45^ site contained ~10% of hybrid N-glycoforms, which is much lower than a similar recombinant HEK293-expressed human FcγRIIIa that contained >50% of hybrid types (32). The predominant Asn^45^ glycoforms were not extensively branched and relatively few were modified with a terminal sialic acid (**Figure 6B**). Asn^64^ likewise contained many complex-type glycoforms and was the most processed among the three glycosylation sites examined, with a complex-type, biantennary di-sialylated glycans among the top forms present. The Asn^162^ glycoforms were highly comparable to the Asn^45^ forms, although they included a smaller percentage of hybrid forms (5-6%). Surprisingly, we observed truncated Asn^64^ (5-15%) and Asn^162^ (3%) glycopeptides. The predominant truncated form only contained a single HexNAc residue. These truncated species are not normally observed for N-glycans and could be representative of an O-linked residue. Closer examination of the Asn^64^ and Asn^162^ MS2 spectra showed the clear presence of a single HexNAc residue attached to Asn^64^ or Asn^162^, eliminating a Ser or Thr-linked O-glycan as a possible explanation (**Supplemental MS2 spectra**). Thus, these glycans potentially resulted from truncation during protein expression or purification.

### The Mm FcγRIII(Val^158^) variant shows greater glycan mobility than the Mm FcγRIII(Ile^158^) as determined by NMR

Next, we analyzed the mobility of glycans attached to the Mm FcγRIII(Val^158^) and Mm FcγRIII(Ile^158^) variants using solution NMR spectroscopy to identify possible differences in conformational heterogeneity (52–55). Uniform amino acid labeling through adding biosynthetic precursors like ammonium chloride for the NMR analysis of glycoproteins, including Mm FcγRIII, is not feasible, but individual [^15^N]-amino acids are readily incorporated from the complex culture medium. We expressed Mm FcγRIII variants as used in structural studies (with Asn^45^, Asn^64^, and Asn^162^ glycosylation sites) in media supplemented with [^15^N]-glycine and [^15^N]-lysine. Glycine readily converts to serine and lysine does not convert to other amino acids in this system (data not shown).

**Figure 7:**
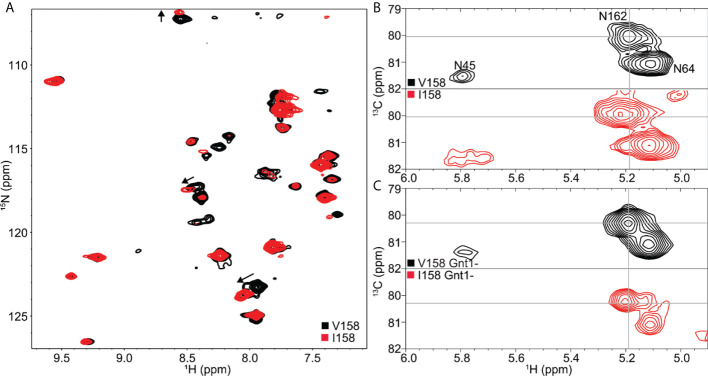
Solution NMR spectroscopy revealed subtle differences between the Mm FcγRIII Val^158^ and Ile^158^ proteins. **(A)** ^1^H-^15^N TROSY-HSQC with [^15^N-Gly, Ser,Lys] labeling. **(B)** HSQC showing (1)GlcNAc ^1^H_1_-^13^C_1_ correlations for Mm FcγRIII with heterogeneous complex-type N-glycans or **(C)** homogeneous Man5 oligomannose-type N-glycans.

The ^1^H-^15^N HSQC-TROSY spectrum of Mm FcγRIII(Val^158^) was well dispersed and showed 31 strong peaks and ~10 weaker peaks for the 44 expected Gly, Ser and Lys residues (**Figure 7A**). This pattern is consistent with a folded protein that contains a mix of α-helices, β-sheets and loop regions. The differences in peak intensities within this spectrum indicate that it is likely that these residues experience different motion regimes. A spectrum of the Mm FcγRIII(Ile^158^) allotype showed a highly comparable pattern of peaks, though some of the weaker peaks did not appear in this spectrum due to a lower sample concentration (240 μM v. 180 μM; **Figure 7A**). Although Val^158^ and Ile^158^ proteins only differ by a single methyl group, there are substantial differences in multiple peak positions that are visible in an overlay of the spectra. There are nine backbone Lys/Gly/Ser NH moieties within 10 Å of Val^158^ and Ile^158^ including Gly^159^-Ser^160^-Lys^161^ that may account for the three largest deviations (noted with arrows in **Figure 7A**).

Unlike amino acids, it is possible to uniformly incorporate [^13^C] labels into the glycoprotein carbohydrates by adding [^13^C]-glucose to the expression medium (56, 57). The most notable spectral feature visible with this strategy is the correlation between the ^1^H_1_-^13^C_1_ nuclei of the GlcNAc residue that is bonded (through C_1_) to the Asn sidechain with one peak expected for each N-glycan in an HSQC spectrum. Observation of these correlations using human FcγRIII identified the presence of contacts that stabilized the Asn^45^ and Asn^162^ glycans, but not the three remaining N-glycans (58). It is notable that it is the Asn^45^ and Asn^162^ glycans that can impact antibody binding affinity (42, 59).

Mm FcγRIII contains three N-glycosylation sites at Asn^45^, Asn^64^ and Asn^162^. A ^13^C-HSQC spectrum reveals three distinct peaks that can be assigned by comparison to a highly comparable spectrum of human FcγRIIIa (58) (**Figure 7B**). The peak corresponding to the Asn^64^ glycan is sharp and intense. The peak position and line shape indicates this N-glycan experiences the least restriction due to contacts with the polypeptide backbone, based on characterizations of human FcγRIIIa and other glycoproteins (54, 58, 60, 61). This behavior is likewise consistent with the MS data showing Asn^64^ as the most highly modified site, including the higher-than-expected abundance of truncated forms which likely requires the glycan to be readily accessible to a glycan hydrolyzing enzyme. The displaced position and broad shape of the Asn^45^ peak likely results from intimate contacts formed between the N-glycan and amino acids (**Figure 2**). Furthermore, this peak position is consistent with the higher level of hybrid and oligomannose N-glycoforms present in the MS data compared to Asn^64^ and Asn^162^. It is notable that the Asn^45^ peak is considerably broader in spectra collected using the Ile^158^ protein (**Figure 7B**).

Intriguingly, the Asn^162^ peak from an Mm FcγRIII(Ile^158^) spectrum is displaced further from the Asn^64^ peak when compared to Mm FcγRIII(Val^158^). This result indicates that the attachment point of the Asn^162^ glycan experiences slightly different chemical environments in each protein. It is not clear if these differences result from the magnetic influence of the extra methyl in Ile^158^ or from differences in protein mobility, though the former explanation is considered less likely due to the ~11 Å distance separating the Ile^158^ methyl and the glycan bond to Asn^162^. These differences were minimized but still present once the glycans were replaced with a minimally-remodeled form (**Figure 7C**). Proteins expressed from the HEK293S (GnT1-) cell line contain predominantly Man5 oligomannose-type N-glycans and nearly homogeneous compositions (42, 62). Based on the differences in spectra collected using ^15^N and ^13^C-labeled samples, we believe it is likely that the Ile^158^ stabilizes the motion of the amino acid and carbohydrate residues proximal to the Asn^162^-glycan more than Val^158^, which could provide an explanation for its greater affinity to IgG.

### The affinity of the allelic Mm FcγRIII(Ile^158^) and (Val^158^) variants for Mm IgGs and Fcs of the Mm IgG subclasses depends upon the receptor glycosylation status

The observation that the residue at position 158, Ile or Val, influenced the chemical shift of the Asn^162^-glycan prompted us to re-examine Mm FcγRIII receptor affinity to IgGs and Fcs initially using the same receptor variants that were used for structural and NMR studies, i.e. with the glycans at Asn^45^, Asn^64^, and Asn^162^ preserved and the glycans at Asn^38^ and Asn^169^ removed by mutagenesis. As with our NMR studies, we used variants expressed in HEK293 cells that produce complex type N-glycans or variants expressed in HEK293 GnT1- cells that produce oligomannose type N-glycans. Removal of the two glycosylation sites, i.e. mutation of Asn^38^ and Asn^169^ to Gln, had an observable effect on the relative affinities of the two variants to macaque IgGs and Fcs even though both of the removed glycans are outside of the binding interface. In comparing the partially glycosylated receptor with complex glycans to our original data with fully glycosylated receptor with complex glycans, we found that with partially glycosylated receptor, the Ile^158^ variant had higher affinity toward IgG than the Val^158^ variant (**Table 1** and **Figure S1**). The higher affinity of the Ile^158^ variant had no exceptions, unlike what was seen for the fully glycosylated receptor. We also observed that the affinity to IgG was lower for partially glycosylated receptors than it was to fully glycosylated receptors (**Table 1**). In contrast, with the exception of the Fc of IgG3, this trend was inverted for receptor binding to Fc, which showed that the Val^158^ variant had the higher affinity. Also, in contrast to IgG, with the exception of the Fc of IgG2, the affinity between fully and partially glycosylated receptor to Fc was greater for the partially glycosylated receptor, emphasizing again the point that receptor affinity to IgG is dependent on regions outside of the Fc.

The change from complex type to oligomannose type glycans on the receptor also affected the receptor’s affinity to IgG. With the exception of IgG3 for both variants and IgG2 for the Ile^158^ variant, the change from complex type to oligomannose type glycans resulted in an increase in affinity to IgG by as much as 1.6-fold. Similar increases in affinity by as much as 3-fold were seen for the binding of both variants to Fc, which led us to ask the question of how glycan composition might influence the affinity of fully glycosylated receptor to IgG. We therefore produced both fully glycosylated receptor variants in HEK293 GnT1- cells and measured their affinity to the macaque IgGs and Fcs of the different IgG subclasses. We found that in all cases, both towards IgG or Fc, the affinity of both variants was the same or greater to the oligomannose type glycan containing receptor than to the complex type glycan containing receptor. This enhancement to the affinity was often greater for the Val^158^ variant and was enough in one case, i.e. for the Fc of IgG3, to change which variant displayed the higher affinity. A similar increase in affinity is seen to human IgG1 when complex type glycans are replaced by oligomannose type glycans on human FcγRIIIa(Val^158^) (42). This increase in affinity is consistent with the NMR data showing greater stabilization of the Asn^162^-glycan in the Ile^158^ variant which suggests that the glycan composition has a greater impact on the Asn^162^-glycan conformation in the Val^158^ variant.

## Discussion

Despite the close evolutionary proximity between humans and Rhesus macaques, *Macacca mulata* (Mm), there are many important differences in immune system genes and function between the two species that can confound the interpretation of results when macaques are used as an animal model. Some have been well documented, such as the gene duplication event that gave rise to FcγRIIIa and FcγRIIIb in humans (12) or the extended hinge region only present in human IgG3 (9, 63), but others such as the effect of IgG glycoform on FcγRIII affinity in macaques have only more recently been examined (64).

Here, we focused on FcγRIII (CD16), the only Fcγ receptor III variant expressed in Mm, and therefore the sole receptor involved in antibody dependent cellular cytotoxicity (ADCC). We utilized the combined methods of x-ray crystallography, NMR, binding affinity measurements and cell activation to describe the molecular differences between the allelic variants of Mm FcγRIII at residue 158 in their ability to form functional complexes with the Fcs of macaque IgG and to mediate ADCC. At first glance, the Val or Ile polymorphism at position 158 is analogous to the human FcγRIIIa polymorphism at the same position, with a higher affinity, more functional Val^158^ and a lower affinity, less functional Phe^158^ variant. In macaque, the variant with the more bulky Ile^158^ (analogous to the Phe^158^ variant in human) shows higher affinity to Mm IgG. However, this increased affinity to IgG only modestly impacted Fc-mediated signaling that leads to ADCC responses (10, 23). Interestingly, our structural analyses of the complexes formed by the Mm FcγRIII Val^158^ and Ile^158^ variants with the Fc of Mm IgG1 confirmed that, as predicted for the human FcγRIIIa Val^158^ and Phe^158^ variants, the less bulky Val side chain at position 158 permits the formation of a ‘tighter’ receptor-Fc complex. Both complexes crystallized in the same C2 space group with two complexes in the asymmetric unit and similar cell dimensions permitting a detailed analyses of the complex interface. The Mm FcγRIII(Val^158^)-Fc(IgG1) complex diffracted slightly better and had a stronger complex interface as measured by the total interface buried surface area. Importantly, we also observed glycan-glycan and glycan-protein interactions at the receptor-Fc interface of the Val^158^ complex that were absent in the Ile^158^ complex interface. Altogether, the data suggested that the affinity of the allelic variants of Mm FcγRIII for Mm IgG could be modulated by the glycosylation status of the receptor, and that the Mm FcγRIII(Val^158^) variant relies more heavily than its Ile^158^ counterpart on this receptor glycan-Fc cross talk. However, a direct comparison of the Mm FcγRIII(Val^158^)-Fc(IgG1) structure to human FcγRIII receptor complexes showed that it more closely resembles human FcγRIIIb(Val^158^)-Fc(IgG1) complexes even though, based on sequence, Mm FcγRIII(Val^158^) is more similar to human FcγRIIIa(Val^158^). Similarly, the Mm FcγRIII(Ile^158^)-Fc(IgG1) complex was closer to the human FcγRIIIb-Fc(IgG1) complexes. These findings suggest that the Mm FcγRIII receptor evolved to incorporate structural features of both human FcγRIIIa and b.

In humans, the glycan composition on the receptor can modulate receptor affinity to IgGs, but this effect is small except in the case of FcγRIIIa (42). FcγRIIIa produced with Man5 type N-glycans has an approximately 10-fold higher K_D_ to IgG1 than FcγRIIIa produced with complex type glycans (42). This is particularly important in light of the finding that human primary NK cells from a subset of donors express FcγRIIIa with a high proportion of oligomannose type glycans (65). Two glycans on human FcγRIIIa have been implicated in this increased affinity, the glycans linked to Asn^45^ and Asn^162^, with much of the increase in affinity being attributed to the glycan on Asn^162^; Asn^162^ is absent in FcγRIIa, b, and c and its mutation in FcγRIIIa abrogates the effect. The macaque FcγRIII has five potential glycosylation sites. Four, Asn^38^, Asn^45^, Asn^162^, and Asn^169^, are analogous to glycosylation sites present in human FcγRIIIa and one, Asn^64^, is analogous to a potential glycosylation site in human FcγRIIIb. We found that similar to human, Mm receptor variants grown in GnT1- cells to produce only Man5 type N-glycans (attached to Asn^45^, Asn^64^ and Asn^162^) had significantly higher affinity to macaque and human IgG as compared to receptor produced in HEK293 cells with complex type glycans. Similar to what is seen for human FcγRIIIa, these changes are mostly attributed to the Asn^162^ glycan.

Although one might suspect that based upon their location and their calculated distance to Fc residues that the glycans attached to Asn^38^ and Asn^169^ should only have a limited contribution to the receptor-Fc complex interface, we instead observed a dramatic effect with their removal both on the receptor’s affinity to IgG and on the relative pattern of interaction for each receptor variant to the Fcs of the Mm IgG subclasses and to human IgG. We observed a slight enhancement of affinity to IgG for both receptor variants, an effect that has also been seen for similar mutations in the human FcγRIIIa (42). Interestingly, changes to the glycan composition (i.e. the switch from complex sugars to Man5 type) was generally greater for the FcγRIII(Val^158^) variant, enough so that it switched the relative affinity of the two variants in one case, making FcγRIII(Val^158^) the higher affinity receptor to the Fc of Mm IgG3, which suggests that glycosylation status and glycan mobility determines the relative affinity to IgG and the functionality of the two Mm FcγRIII variants. Indeed, our NMR studies showed that Ile^158^ restricts mobility of the glycan attached to Asn^162^ more than what is seen for the Val^158^ variant.

Previous work has shown that the macaque FcγRIII has higher affinity for macaque IgG1 compared with IgGs 2-4 (10) and for human IgG1 compared with macaque IgG1 (23). We observed that the change from complex type glycans on the receptor to Man5 type changed this relative preference making the Fcs of macaque IgG4 more similar in affinity to human IgG1. This enhancement was greater for the Val^158^ variant. One structural difference that could possibly explain this change is related to the stability of the glycan on the Fc. In the human Fc Asn^297^ in the DE loop is stabilized by a hydrogen bond between His^268^ of the BC loop and Glu^294^ in the DE loop. In the macaque IgG1, a similar but weaker hydrogen bond is possible between Gln^268^ and Thr^294^. Examination of these residues in the four copies of the FcγRIII-Fc(IgG1) complex in the two different crystal forms shows this bond in only one copy of monomer A of the Fc, suggesting it has a smaller contribution to Fc glycan stability in the macaque complex. Human IgG2 and IgG3 also have a stabilizing hydrogen bond between His^268^ and Glu^294^ and human IgG4 has a potential one between Gln^268^ and Glu^294^. Macaque IgG2 has an analogous hydrogen bond between Gln^268^ and Glu^294^and macaque IgGs 3 and 4 one between Gln^268^ and Arg^294^. So, in contrast to human IgGs, where the Asn^297^ stabilizing hydrogen bond is of identical or similar length between the different IgG subclasses, macaques have a weaker stabilizing bond for IgG1 as compared to IgG2, IgG3, and IgG4. Stabilizing the Fc glycan might be a factor that contributes to the higher affinity for the Man5 produced receptor to the macaque IgG2, IgG3, and IgG4 as compared to IgG1 and to the Val^158^ variant. In summary, as has been the case for afucosylated glycans on the Fc for macaque IgGs (64), changes to the glycan composition on the macaque FcγRIII receptor can lead to changes in affinity to IgG. It remains to be seen if this change in affinity will translate to a change in function, particularly with regard to NK cells and their potentially altered FcγRIII glycan composition as has been the case for humans (65). An important consideration is how glycan composition might change Fc-effector activity with regard to IgG subtype switching, and how in macaques as IgG1s switch to other subclasses, antibodies with enhanced NK cell mediated activity could be generated, which is different to what is seen in humans.

## Data availability statement

The datasets and structures generated during the current study are available in the Protein Data Bank (PDB) repository with PDB IDs 6MJ3, 7KCZ, and 6MJO.

## Author contributions

WT, NG, AB, and MP designed, performed research and analyzed the data; AH, advised by MA, performed and analyzed BLI binding data; PK and AB designed, performed and interpreted the MS and NMR experiments, VV solved structure of apo RM FcγRIII; DN, refined and analyzed structures; RS, GL, and AF helped design experiments and/or provided unique reagents/methodologies; JP performed and analyzed cell binding and ADCC data; WT, DN, and MP wrote the manuscript and all authors provided comments or revisions. All authors contributed to the article and approved the submitted version.

## Funding

Funding for this study was provided by the National Institute of Health grants: P01 AI120756 to MP and MA; R01 AI116274 to MP and R01 AI129769 to MP and AF; and U01 AI148114 to AB. The funders had no role in study design, data collection and analysis, decision to publish, or preparation of the manuscript and the contents of this publication are solely the responsibility of the authors.

## Acknowledgments

Use of the Stanford Synchrotron Radiation Lightsource, SLAC National Accelerator Laboratory, is supported by the U.S. Department of Energy, Office of Science, Office of Basic Energy Sciences under Contract No. DE-AC02-76SF00515. The SSRL Structural Molecular Biology Program is supported by the DOE Office of Biological and Environmental Research, and by the National Institutes of Health, National Institute of General Medical Sciences. We thank Nicole Rodgers for technical assistance with the NK-92 cell line experiments.

## Conflict of interest

The authors declare that the research was conducted in the absence of any commercial or financial relationships that could be construed as a potential conflict of interest.

## Publisher’s note

All claims expressed in this article are solely those of the authors and do not necessarily represent those of their affiliated organizations, or those of the publisher, the editors and the reviewers. Any product that may be evaluated in this article, or claim that may be made by its manufacturer, is not guaranteed or endorsed by the publisher.

## Author disclaimer

The views expressed in this manuscript are those of the authors and do not reflect the official policy or position of the Uniformed Services University, US Army, the Department of Defense, or the US Government.
