# Supplementary material for: Decoding human-macaque interspecies differences in Fc-effector functions: The structural basis for CD16-dependent effector function in Rhesus macaques
Source: Front Immunol. 2022 Sep 5;13:960411. doi: 10.3389/fimmu.2022.960411 (PMC9484259; doi:10.3389/fimmu.2022.960411)
Supplement: Supplementary file 1 [file DataSheet_1.pdf]

Supplementary Material

1. Supplementary Figures and Tables

1.1.Supplementary Tables

Table S1. Binding kinetics of the allelic Mm FcγRIII Ile158 and Val158 variants to the Mm IgGs and Fcs of the Mm IgG subclasses. Equilibrium binding kinetics: equilibrium constant (K_D), association constant (K_{on}) and dissociation constant (k_{off}) were calculated from by SPR measurement. Data are average of 2 independent experiments.

Mm FcγRIII variant		Mm IgG1		Mm IgG2		Mm IgG3		Mm IgG4	
		Fc	IgG	Fc	IgG	Fc	IgG	Fc	IgG
Ile158 fully glycosylated, complex glycans	K_D (mM)	0.25	0.26	0.23	1.8	0.4	1.9	1.8	0.98
	K_{on}(1/Ms)	2.33E+05	3.64E+0	2.81E+0	1.00E+0	9.18E+0	7.60E+0	9.09E+0	1.10E+0
	K_{off}(1/s)	4.17E-03	5.84E-03	5.13E-03	1.00E-03	5.77E-02	7.36E-02	1.83E-01	1.10E-01
Val158 fully glycosylated, complex glycans	K_D (mM)	0.36	0.41	0.09	2.1	0.46	3.2	0.63	0.57
	K_{on}(1/Ms)	2.07E+05	1.19E+0	3.75E+0	6.10E+0	2.62E+0	4.68E+0	2.88E+0	2.45E+0
	K_{off}(1/s)	3.96E-03	5.95E-03	5.15E-03	6.30E-02	3.00E-01	7.40E-02	6.37E-01	3.01E-01
Ile158 fully glycosylated, Man5 glycans	K_D (mM)	0.21	0.22	0.15	1.5	0.29	1.9	1.1	0.49
	K_{on}(1/Ms)	2.66E+05	1.84E+0	8.46E+0	4.98E+0	9.96E+0	8.55E+0	8.37E+0	8.72E+0
	K_{off}(1/s)	4.23E-03	5.68E-03	5.87E-03	3.29E-02	5.06E-02	1.27E-01	6.35E-02	4.37E-02
Val158 fully glycosylated, Man5 glycans	K_D (mM)	0.3	0.28	0.08	1.8	0.24	2.3	0.53	0.3
	K_{on}(1/Ms)	2.10E+05	1.90E+0	2.27E+0	4.94E+0	2.37E+0	9.27E+0	1.73E+0	1.37E+0
	K_{off}(1/s)	4.19E-03	5.23E-03	5.23E-03	1.77E-02	4.30E-02	1.66E-01	3.82E-02	8.75E-02
Ile158 partial* complex glycans	K_D (mM)	0.12	0.34	1.6	2.3	0.23	2.8	2.2	2.3
	K_{on}(1/Ms)	5.00E+05	1.75E+0	3.97E+0	2.03E+0	7.36E+0	6.75E+0	5.26E+0	3.25E+0
	K_{off}(1/s)	5.43E-03	4.13E-03	1.86E-02	2.78E-02	4.28E-03	8.13E-01	6.34E-01	3.18E-02
Val158 partial* complex glycans	K_D (mM)	0.03	0.46	1.2	2.6	0.25	4.2	1	3
	K_{on}(1/Ms)	1.16E+06	1.76E+0	8.42E+0	2.54E+0	9.95E+0	5.19E+0	3.05E+0	7.24E+0
	K_{off}(1/s)	5.37E-03	5.17E-03	1.36E-02	3.85E-02	4.18E-03	7.08E-02	2.92E-01	4.07E-02
Ile158 partial* Man5 glycans	K_D (mM)	0.06	0.28	1.5	2.8	0.16	3	1.9	1.7
	K_{on}(1/Ms)	2.07E+05	2.94E+0	2.56E+0	3.02E+0	9.59E+0	6.62E+0	1.44E+0	8.29E+0
	K_{off}(1/s)	4.75E-03	4.61E-03	1.13E-02	2.27E-02	4.42E-03	1.98E-01	1.55E-01	1.66E-01
Val158 partial* Man5 glycans	K_D (mM)	0.01	0.29	0.61	2.2	0.28	4.8	0.85	2.2
	K_{on}(1/Ms)	3.28E+05	1.64E+0	3.71E+0	3.27E+0	1.03E+0	4.77E+0	4.06E+0	1.12E+0
	K_{off}(1/s)	5.13E-03	4.64E-03	1.32E-02	3.99E-02	5.42E-03	1.41E-01	8.14E-02	7.41E-02

Table S2: Details of the macaque FcγRIII(Val158)/IgG1Fc and FcγRIII(Ile158)/IgG1Fc complexes as compared to the human FcγRIIIa(Val158)/IgG1Fc and FcγRIIIb/IgG1Fc complexes (PDB IDs 5XJE and 6EAQ). Buried surface areas were calculated using the EBI PISA server (http://www.ebi.ac.uk/msd-srv/prot_int/cgi-bin/piserver). BSA contributions from the Fc are split into contributions from chain A and chain B of the Fc dimer. The macaque FcγRIII BSA contributions are an average of the two complexes in the asymmetric unit. Values in parenthesis represent the contributions from glycans.

		Macaque FcγRIII (V158)/ IgG1Fc (PDB 7KCZ)		Macaque FcγRIII (I158)/ IgG1Fc (PDB 6MJ3)		Human FcγRIIIa (V158)/ IgG1Fc (PDB 5XJE)		Human FcγRIIIb/ IgG1Fc (PDB 6EAQ)	
**Buried Surface Area, Å^2**		**A**	**B**	**A**	**B**	**A**	**B**	**A**	**B**
	**Fc total**	**576**	**346**	**425**	**342**	**629**	**394**	**738**	**367**
	Fc protein	484	329	356	323	525	381	497	367
	Fc N-terminus	152	116	77	109	163	180	141	176
	Fc BC loop	77	0	57	0	88	0	96	0
	Fc DE loop	193	0	158	0	208	0	202	0
	Fc FG loop	19	209	23	213	26	201	19	191
	Fc glycan (Asn^{297})	(92)	(17)	(69)	(19)	(104)	(13)	(241)	(0)
	**FcγRIII total**	**590**	**320**	**414**	**317**	**647**	**371**	**771**	**331**
	FcγRIII protein	518	297	365	290	550	357	525	331
	FcγRIII glycan (Asn^{162})	(72)	(23)	(49)	(27)	(97)	(14)	(246)	(0)
	**FcγRIII and Fc total**	**1,166 (164)**	**666 (40)**	**839 (118)**	**659 (46)**	**1,276 (201)**	**765 (27)**	**1,509 (487)**	**698 (0)**
	**average**	**1,832 (204)**		**1,498 (164)**		**2,041 (228)**		**2,207(487)**	

1.2. Supplementary Figures

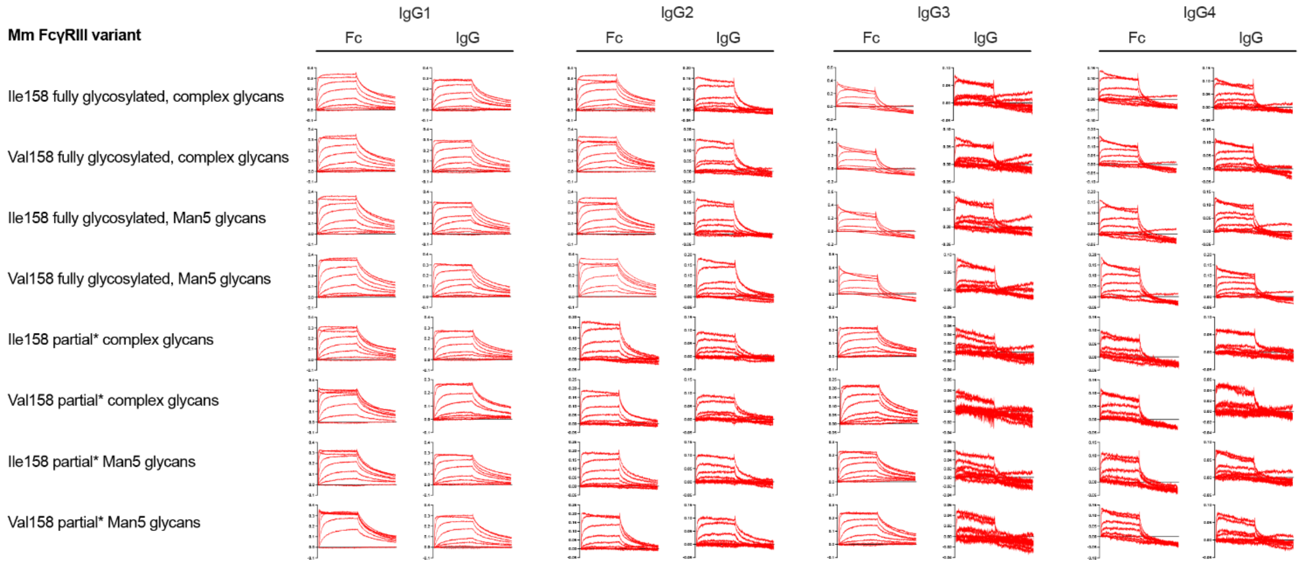

Figure S1. BLI sensorgrams (red) of binding of RM IgGs and Fcs to RM FcγRIII variants. Assessments were performed across 3-fold serial dilutions of FcγR ranging from 10 mM to 0.013 mM.

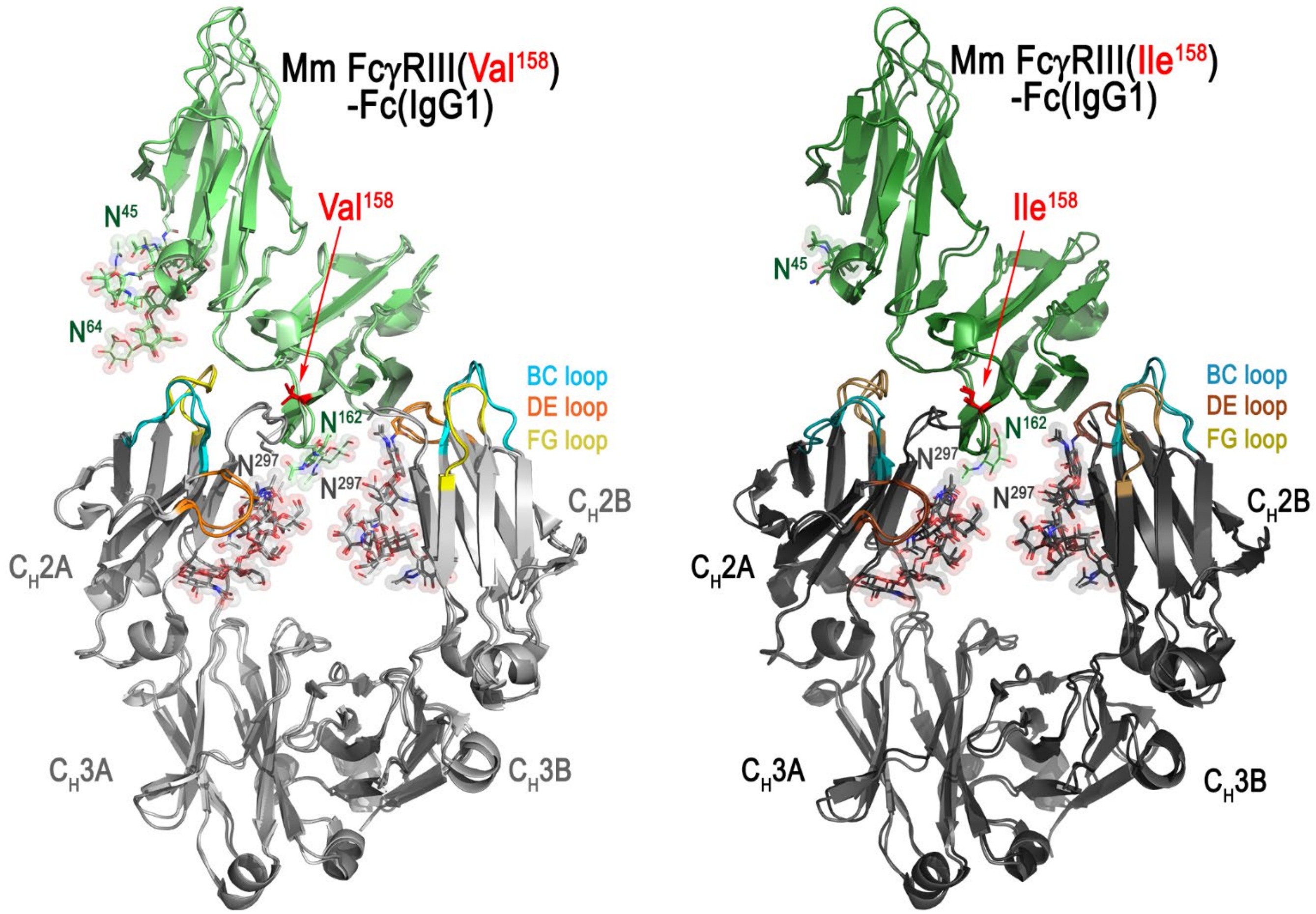

Figure S2. Structural comparison of the crystallographic copies of Mm FcγRIII(Va^{l58})-Fc(IgG1) (left) and Mm FcγRIII(Ile^{l58})-Fc(IgG1) (right) complexes. The complexes are shown as a ribbon diagram with the Mm FcγRIII receptors in light green (FcγRIII(Val^{158})) and dark green (FcγRIII(Ile^{158})), and the two Fcs (C_H2-C_H3 domains) in light gray (Mm FcγRIII(Val^{158})-Fc(IgG1) complex) and dark gray (Mm FcγRIII(Ile^{158})-Fc(IgG1) complex). Sugars attached to Asn^{297}, Asn^{162}, Asn^{45} and Asn^{64} residues are shown as spheres colored by atom type (backbone color for carbon, red for oxygen and blue for nitrogen). The BC, DE, and FG loops of the C_H2 domain in FcγRIII(Val^{158})-Fc(IgG1) and FcγRIII(Ile^{158})-Fc(IgG1) structures are colored in light and dark shades of cyan, yellow, and orange, respectively. The two Fc monomers are labeled as A and B chains.

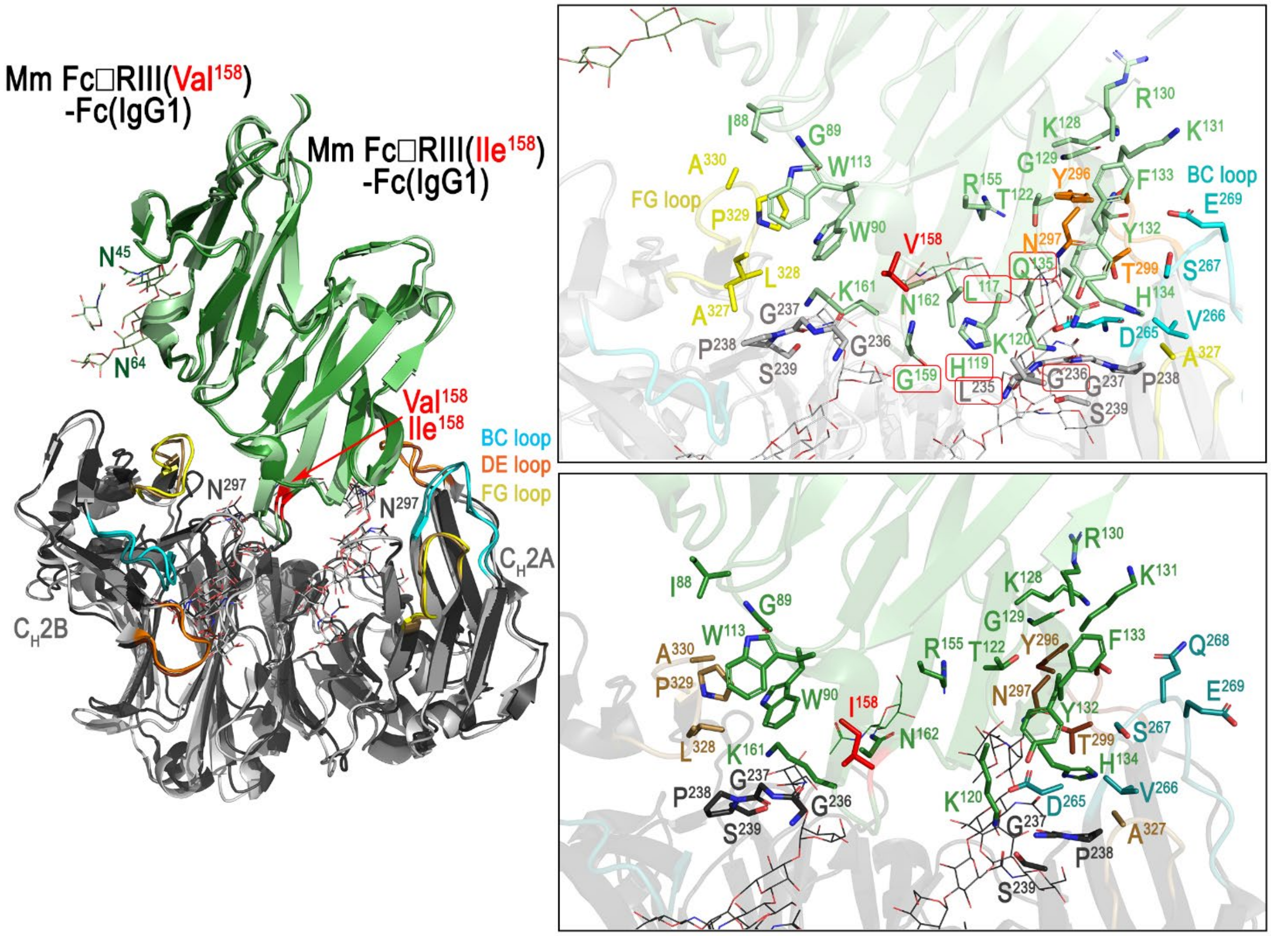

Figure S3. Structural comparison of the Mm FcγRIII(Val^{158})-Fc(IgG1) and Mm FcγRIII(Ile^{158})-Fc(IgG1) complexes. Superimposition of the two Mm FcγRIII-Fc(C$_H$2 domain) complexes shown as a ribbon diagram with a color scheme similar to Figure S1. The two heavy chain monomers are labeled as A and B chains. The right two panels show the interfaces of the Mm FcγRIII(Val^{158})-Fc(IgG1) (top) and Mm FcγRIII(Ile^{158})-Fc(IgG1) (bottom)complexes respectively. Residues contributing to buried surface binding are shown as sticks.
