# Supplementary material for: Decoding human-macaque interspecies differences in Fc-effector functions: The structural basis for CD16-dependent effector function in Rhesus macaques
Source: Front Immunol. 2022 Sep 5;13:960411. doi: 10.3389/fimmu.2022.960411 (PMC9484259; doi:10.3389/fimmu.2022.960411)
Supplement: Supplementary file 3 [file DataSheet_3.pdf]

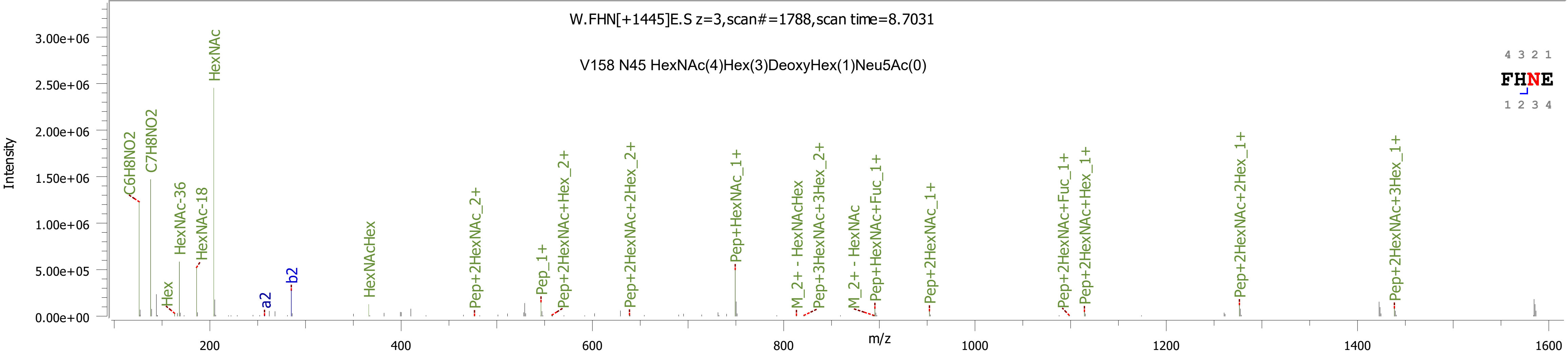

W.FHN[+1445]E.S z=3,scan#=1788,scan time=8.7031
V158 N45 HexNAc(4)Hex(3)DeoxyHex(1)Neu5Ac(0)
4 3 2 1
FHNE
1 2 3 4
Intensity
m/z
C6H8NO2
C7H8NO2
Hex
HexNAc-36
HexNAc-18
HexNAc
a2
b2
HexNAcHex
Pep+2HexNAc_2+
Pep_1+
Pep+2HexNAc+Hex_2+
Pep+2HexNAc+2Hex_2+
Pep+HexNAc_1+
M_2+ - HexNAcHex
Pep+3HexNAc+3Hex_2+
M_2+ - HexNAc
Pep+HexNAc+Fuc_1+
Pep+2HexNAc_1+
Pep+2HexNAc+Fuc_1+
Pep+2HexNAc+Hex_1+
Pep+2HexNAc+2Hex_1+
Pep+2HexNAc+3Hex_1+

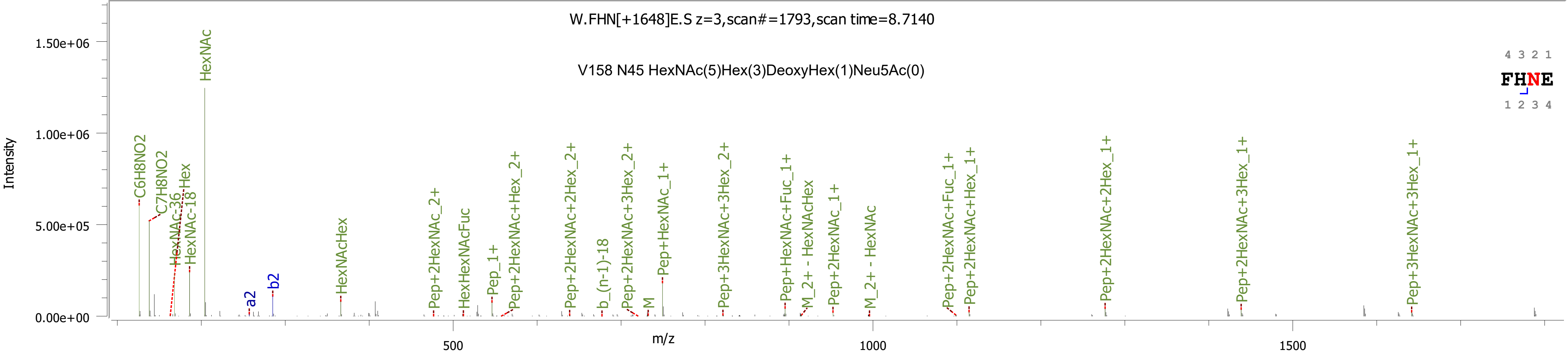
W.FHN[+1648]E.S z=3,scan#=1793,scan time=8.7140
V158 N45 HexNAc(5)Hex(3)DeoxyHex(1)Neu5Ac(0)
4 3 2 1
FHNE
1 2 3 4
Intensity
1.50e+06
1.00e+06
5.00e+05
0.00e+00
m/z
500
1000
1500
C6H8NO2
C7H8NO2
HexNAc-36
HexNAc-18
Hex
HexNAc
a2
b2
HexNAcHex
Pep+2HexNAc_2+
HexHexNAcFuc
Pep_1+
Pep+2HexNAc+Hex_2+
Pep+2HexNAc+2Hex_2+
b_(n-1)-18
Pep+2HexNAc+3Hex_2+
M
Pep+HexNAc_1+
Pep+3HexNAc+3Hex_2+
Pep+HexNAc+Fuc_1+
M_2+ - HexNAcHex
Pep+2HexNAc_1+
M_2+ - HexNAc
Pep+2HexNAc+Fuc_1+
Pep+2HexNAc+Hex_1+
Pep+2HexNAc+2Hex_1+
Pep+2HexNAc+3Hex_1+
Pep+3HexNAc+3Hex_1+

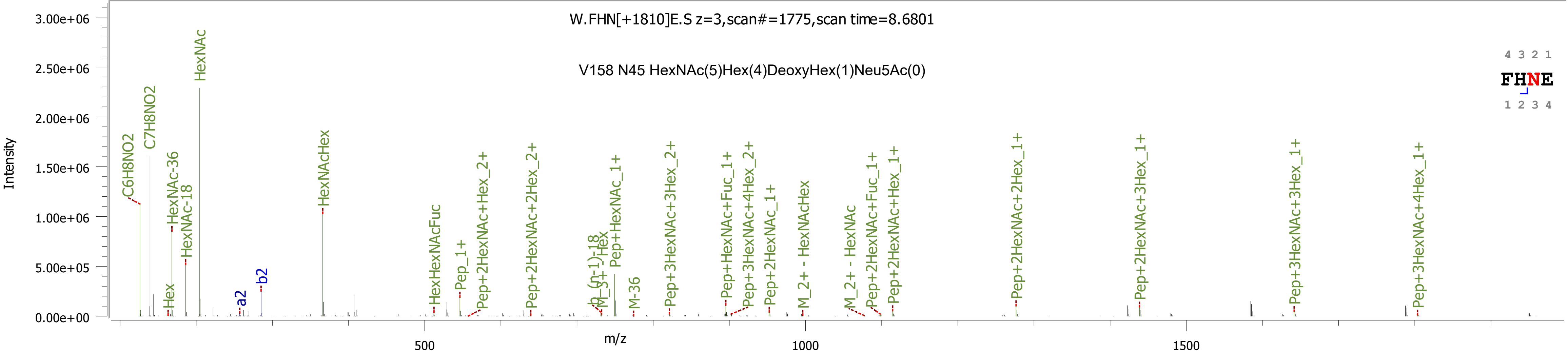
W.FHN[+1810]E.S z=3,scan#=1775,scan time=8.6801
V158 N45 HexNAc(5)Hex(4)DeoxyHex(1)Neu5Ac(0)
4 3 2 1
FHNE
1 2 3 4
Intensity
m/z
3.00e+06
2.50e+06
2.00e+06
1.50e+06
1.00e+06
5.00e+05
0.00e+00
500
1000
1500
C6H8NO2
C7H8NO2
HexNAc
Hex
HexNAc-36
HexNAc-18
a2
b2
HexNAcHex
HexHexNAcFuc
Pep_1+
Pep+2HexNAc+Hex_2+
Pep+2HexNAc+2Hex_2+
M_3+
Hex
Pep+HexNAc_1+
M-36
Pep+3HexNAc+3Hex_2+
Pep+HexNAc+Fuc_1+
Pep+3HexNAc+4Hex_2+
Pep+2HexNAc_1+
M_2+ - HexNAcHex
M_2+ - HexNAc
Pep+2HexNAc+Fuc_1+
Pep+2HexNAc+Hex_1+
Pep+2HexNAc+2Hex_1+
Pep+2HexNAc+3Hex_1+
Pep+3HexNAc+3Hex_1+
Pep+3HexNAc+4Hex_1+

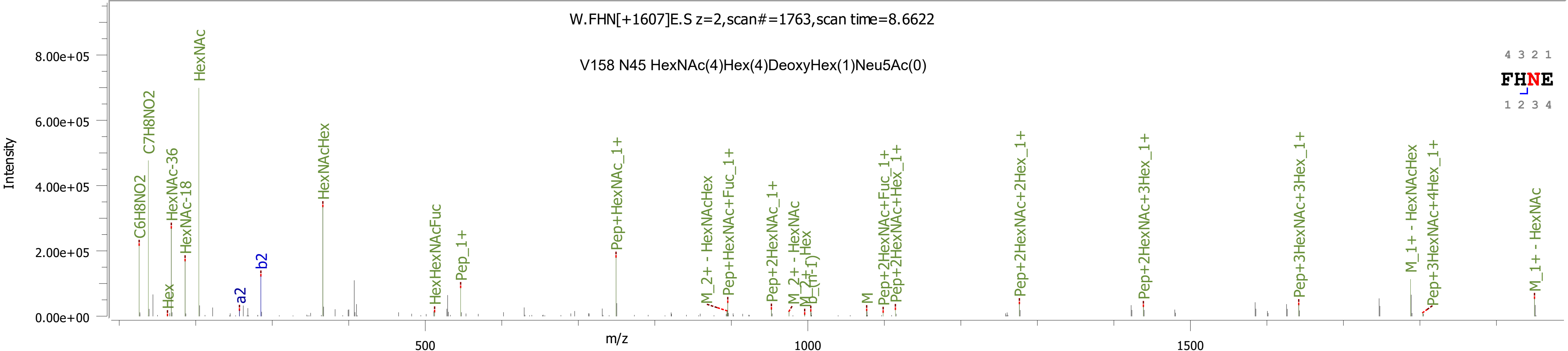

W.FHN[+1607]E.S z=2,scan#=1763,scan time=8.6622
V158 N45 HexNAc(4)Hex(4)DeoxyHex(1)Neu5Ac(0)
4 3 2 1
FHNE
1 2 3 4
Intensity
8.00e+05
6.00e+05
4.00e+05
2.00e+05
0.00e+00
m/z
500
1000
1500
C6H8NO2
C7H8NO2
Hex
HexNAc-36
HexNAc-18
HexNAc
a2
b2
HexNAcHex
HexHexNAcFuc
Pep_1+
Pep+HexNAc_1+
M_2+ - HexNAcHex
Pep+HexNAc+Fuc_1+
Pep+2HexNAc_1+
M_2+ - HexNAc
M_2+ - Hex
b_(n-1)
M
Pep+2HexNAc+Fuc_1+
Pep+2HexNAc+Hex_1+
Pep+2HexNAc+2Hex_1+
Pep+2HexNAc+3Hex_1+
Pep+3HexNAc+3Hex_1+
M_1+ - HexNAcHex
Pep+3HexNAc+4Hex_1+
M_1+ - HexNAc

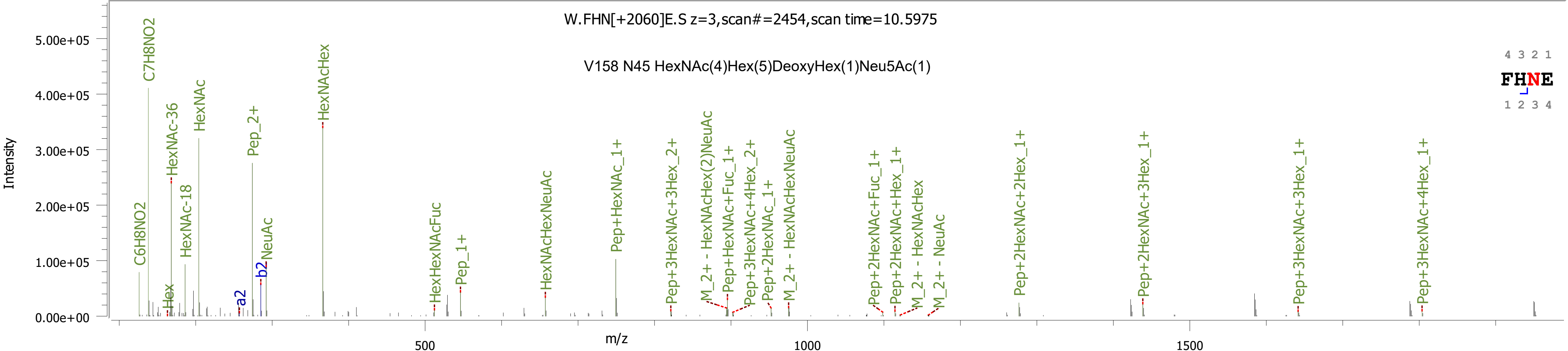
W.FHN[+2060]E.S z=3,scan#=2454,scan time=10.5975
V158 N45 HexNAc(4)Hex(5)DeoxyHex(1)Neu5Ac(1)
FHNE
4 3 2 1
1 2 3 4
Intensity
m/z
0.00e+00
1.00e+05
2.00e+05
3.00e+05
4.00e+05
5.00e+05
500
1000
1500
C6H8NO2
C7H8NO2
Hex
HexNAc-36
HexNAc-18
HexNAc
a2
Pep_2+
b2
NeuAc
HexNAcHex
HexHexNAcFuc
Pep_1+
HexNAcHexNeuAc
Pep+HexNAc_1+
Pep+3HexNAc+3Hex_2+
M_2+ - HexNAcHex(2)NeuAc
Pep+HexNAc+Fuc_1+
Pep+3HexNAc+4Hex_2+
Pep+2HexNAc_1+
M_2+ - HexNAcHexNeuAc
Pep+2HexNAc+Fuc_1+
Pep+2HexNAc+Hex_1+
M_2+ - HexNAcHex
M_2+ - NeuAc
Pep+2HexNAc+2Hex_1+
Pep+2HexNAc+3Hex_1+
Pep+3HexNAc+3Hex_1+
Pep+3HexNAc+4Hex_1+

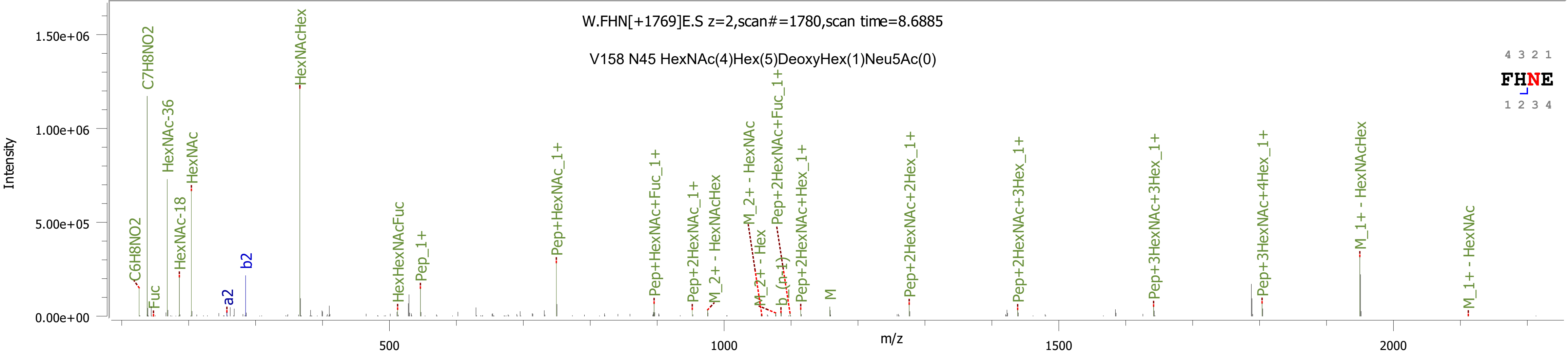
W.FHN[+1769]E.S z=2,scan#=1780,scan time=8.6885
V158 N45 HexNAc(4)Hex(5)DeoxyHex(1)Neu5Ac(0)
Intensity
m/z
1.50e+06
1.00e+06
5.00e+05
0.00e+00
500
1000
1500
2000
C6H8NO2
C7H8NO2
Fuc
HexNAc-36
HexNAc-18
HexNAc
a2
b2
HexNAcHex
HexHexNAcFuc
Pep_1+
Pep+HexNAc_1+
Pep+HexNAc+Fuc_1+
Pep+2HexNAc_1+
M_2+ - HexNAcHex
M_2+ - HexNAc
M_2+ - Hex
b_(n-1)
Pep+2HexNAc+Fuc_1+
Pep+2HexNAc+Hex_1+
M
Pep+2HexNAc+2Hex_1+
Pep+2HexNAc+3Hex_1+
Pep+3HexNAc+3Hex_1+
Pep+3HexNAc+4Hex_1+
M_1+ - HexNAcHex
M_1+ - HexNAc
4 3 2 1
FHNE
1 2 3 4

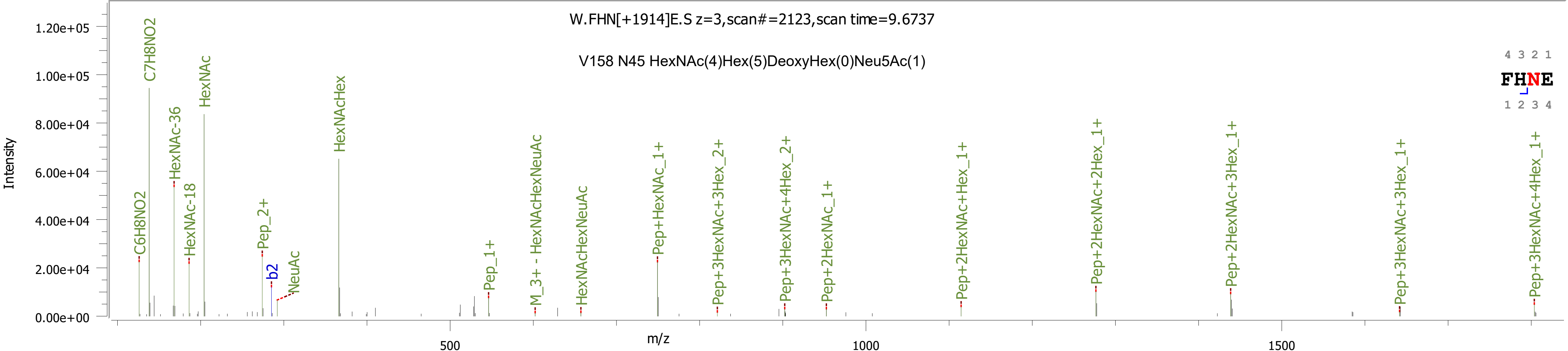
W.FHN[+1914]E.S z=3,scan#=2123,scan time=9.6737
V158 N45 HexNAc(4)Hex(5)DeoxyHex(0)Neu5Ac(1)
4 3 2 1
FHNE
1 2 3 4
Intensity
1.20e+05
1.00e+05
8.00e+04
6.00e+04
4.00e+04
2.00e+04
0.00e+00
m/z
500
1000
1500
C6H8NO2
C7H8NO2
HexNAc-36
HexNAc-18
HexNAc
Pep_2+
b2
NeuAc
HexNAcHex
Pep_1+
M_3+ - HexNAcHexNeuAc
HexNAcHexNeuAc
Pep+HexNAc_1+
Pep+3HexNAc+3Hex_2+
Pep+3HexNAc+4Hex_2+
Pep+2HexNAc_1+
Pep+2HexNAc+Hex_1+
Pep+2HexNAc+2Hex_1+
Pep+2HexNAc+3Hex_1+
Pep+3HexNAc+3Hex_1+
Pep+3HexNAc+4Hex_1+

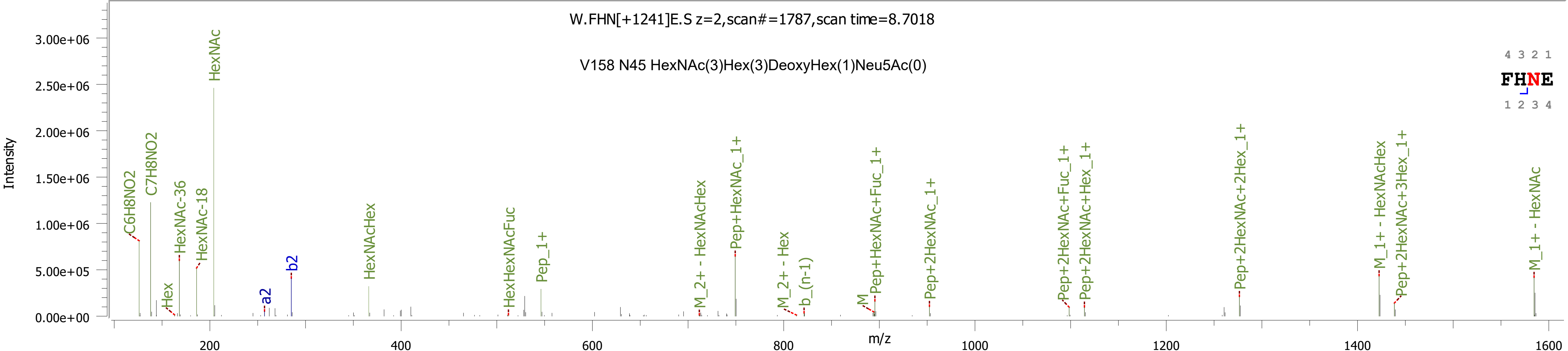
W.FHN[+1241]E.S z=2,scan#=1787,scan time=8.7018
V158 N45 HexNAc(3)Hex(3)DeoxyHex(1)Neu5Ac(0)
4 3 2 1
FHNE
1 2 3 4
Intensity
m/z
C6H8NO2
C7H8NO2
Hex
HexNAc-36
HexNAc-18
HexNAc
a2
b2
HexNAcHex
HexHexNAcFuc
Pep_1+
M_2+ - HexNAcHex
Pep+HexNAc_1+
M_2+ - Hex
b_(n-1)
M
Pep+HexNAc+Fuc_1+
Pep+2HexNAc_1+
Pep+2HexNAc+Fuc_1+
Pep+2HexNAc+Hex_1+
Pep+2HexNAc+2Hex_1+
M_1+ - HexNAcHex
Pep+2HexNAc+3Hex_1+
M_1+ - HexNAc
0.00e+00
5.00e+05
1.00e+06
1.50e+06
2.00e+06
2.50e+06
3.00e+06
200
400
600
800
1000
1200
1400
1600

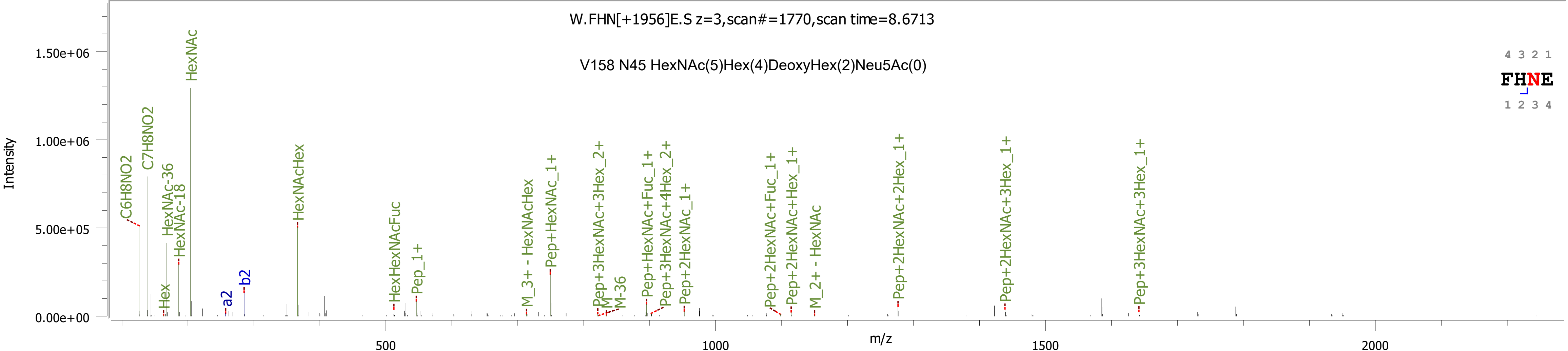
W.FHN[+1956]E.S z=3,scan#=1770,scan time=8.6713
V158 N45 HexNAc(5)Hex(4)DeoxyHex(2)Neu5Ac(0)
FHNE
Intensity
m/z
1.50e+06
1.00e+06
5.00e+05
0.00e+00
500
1000
1500
2000
C6H8NO2
C7H8NO2
Hex
HexNAc-36
HexNAc-18
HexNAc
a2
b2
HexNAcHex
HexHexNAcFuc
Pep_1+
M_3+ - HexNAcHex
Pep+HexNAc_1+
Pep+3HexNAc+3Hex_2+
M-36
Pep+HexNAc+Fuc_1+
Pep+3HexNAc+4Hex_2+
Pep+2HexNAc_1+
Pep+2HexNAc+Fuc_1+
Pep+2HexNAc+Hex_1+
M_2+ - HexNAc
Pep+2HexNAc+2Hex_1+
Pep+2HexNAc+3Hex_1+
Pep+3HexNAc+3Hex_1+

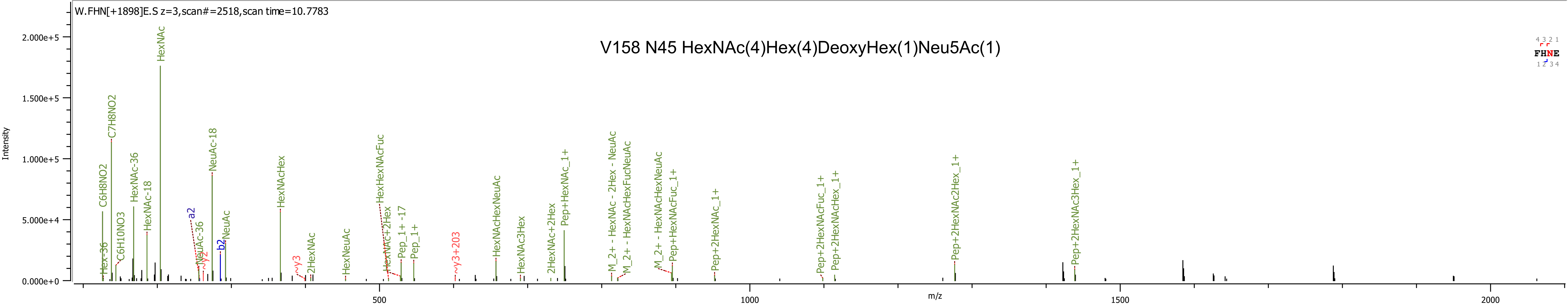
W.FHN[+1898]E.S z=3,scan#=2518,scan time=10.7783
V158 N45 HexNAc(4)Hex(4)DeoxyHex(1)Neu5Ac(1)
FHNE
Intensity
m/z
HexNAc
C7H8NO2
C6H8NO2
HexNAc-36
HexNAc-18
NeuAc-18
a2
NeuAc
b2
NeuAc-36
C6H10NO3
Hex-36
~y2
HexNAcHex
~y3
2HexNAc
HexNeuAc
HexHexNAcFuc
HexNAc+2Hex
Pep_1+ -17
Pep_1+
~y3+203
HexNAcHexNeuAc
HexNAc3Hex
2HexNAc+2Hex
Pep+HexNAc_1+
M_2+ - HexNAc - 2Hex - NeuAc
M_2+ - HexNAcHexFucNeuAc
M_2+ - HexNAcHexNeuAc
Pep+HexNAcFuc_1+
Pep+2HexNAc_1+
Pep+2HexNAcFuc_1+
Pep+2HexNAcHex_1+
Pep+2HexNAc2Hex_1+
Pep+2HexNAc3Hex_1+

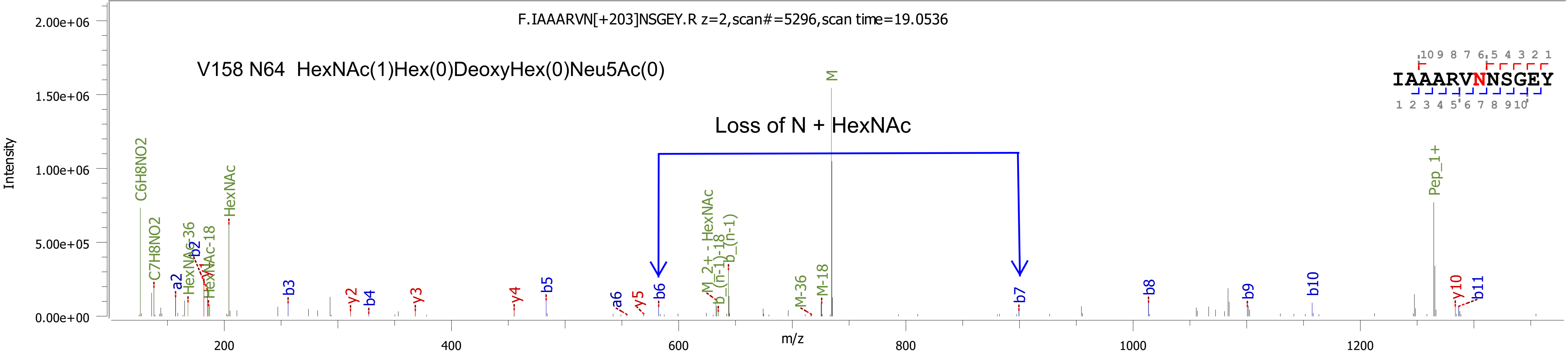

F.IAAARVN[+203]NSGEY.R z=2,scan#=5296,scan time=19.0536
V158 N64 HexNAc(1)Hex(0)DeoxyHex(0)Neu5Ac(0)
Loss of N + HexNAc
IAAARVNNSGEY
Intensity
m/z
M
Pep_1+
C6H8NO2
C7H8NO2
HexNAc
HexNAc-36
HexNAc-18
M_2+ - HexNAc
b_(n-1)-18
b_(n-1)
M-36
M-18
a2
b2
b3
b4
b5
a6
b6
b7
b8
b9
b10
b11
y2
y3
y4
y5
y10

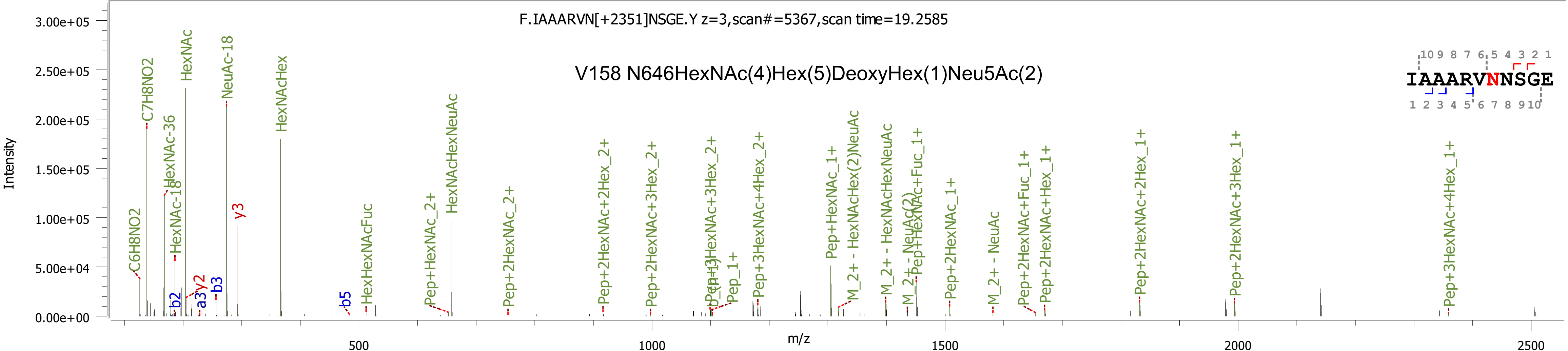
F.IAAARVN[+2351]NSGE.Y z=3,scan#=5367,scan time=19.2585
V158 N646HexNAc(4)Hex(5)DeoxyHex(1)Neu5Ac(2)
IAAARVNNSGE
Intensity
m/z
C6H8NO2
C7H8NO2
HexNAc-36
HexNAc-18
HexNAc
NeuAc-18
HexNAcHex
HexHexNAcFuc
Pep+HexNAc_2+
HexNAcHexNeuAc
Pep+2HexNAc_2+
Pep+2HexNAc+2Hex_2+
Pep+2HexNAc+3Hex_2+
Pep+3HexNAc+3Hex_2+
Pep_1+
Pep+3HexNAc+4Hex_2+
Pep+HexNAc_1+
M_2+ - HexNAcHex(2)NeuAc
M_2+ - HexNAcHexNeuAc
M_2+ - NeuAc(2)
Pep+HexNAc+Fuc_1+
Pep+2HexNAc_1+
M_2+ - NeuAc
Pep+2HexNAc+Fuc_1+
Pep+2HexNAc+Hex_1+
Pep+2HexNAc+2Hex_1+
Pep+2HexNAc+3Hex_1+
Pep+3HexNAc+4Hex_1+
b2
a3
y2
b3
y3
b5

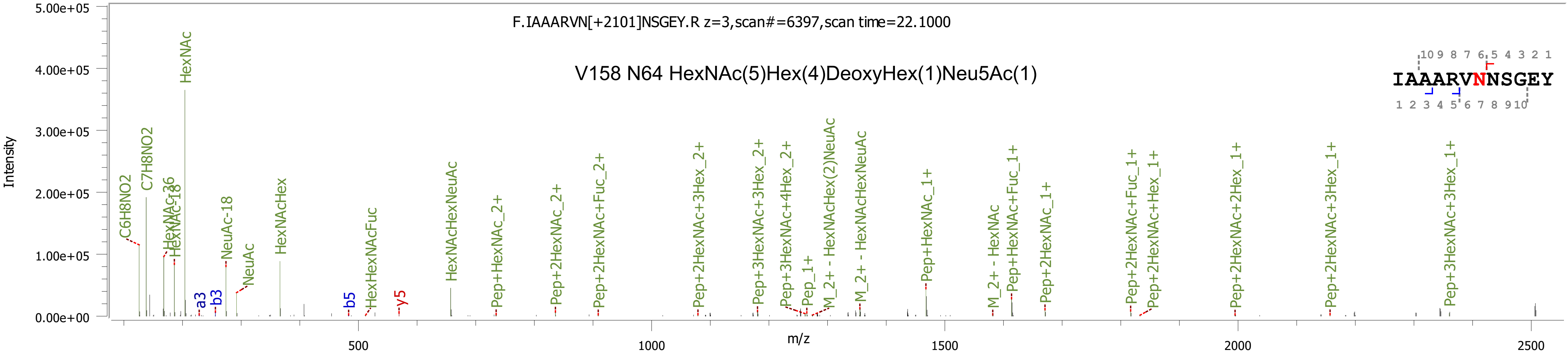
F.IAAARVN[+2101]NSGEY.R z=3,scan#=6397,scan time=22.1000
V158 N64 HexNAc(5)Hex(4)DeoxyHex(1)Neu5Ac(1)
IAAARVNNSGEY
Intensity
m/z
5.00e+05
4.00e+05
3.00e+05
2.00e+05
1.00e+05
0.00e+00
500
1000
1500
2000
2500
C6H8NO2
C7H8NO2
HexNAc-36
HexNAc-18
HexNAc
a3
b3
NeuAc-18
NeuAc
HexNAcHex
b5
HexHexNAcFuc
y5
HexNAcHexNeuAc
Pep+HexNAc_2+
Pep+2HexNAc_2+
Pep+2HexNAc+Fuc_2+
Pep+2HexNAc+3Hex_2+
Pep+3HexNAc+3Hex_2+
Pep+3HexNAc+4Hex_2+
Pep_1+
M_2+ - HexNAcHex(2)NeuAc
M_2+ - HexNAcHexNeuAc
Pep+HexNAc_1+
M_2+ - HexNAc
Pep+HexNAc+Fuc_1+
Pep+2HexNAc_1+
Pep+2HexNAc+Fuc_1+
Pep+2HexNAc+Hex_1+
Pep+2HexNAc+2Hex_1+
Pep+2HexNAc+3Hex_1+
Pep+3HexNAc+3Hex_1+

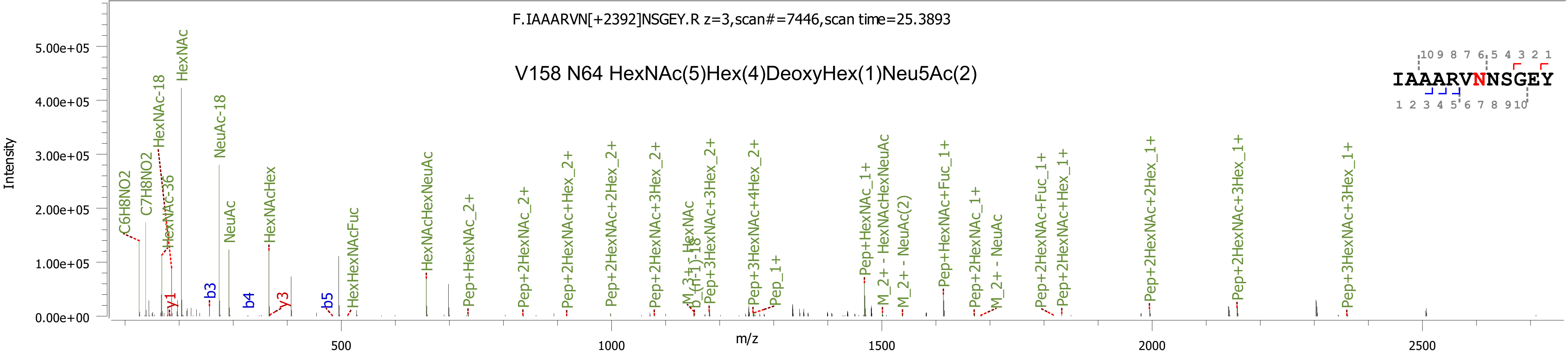
F.IAAARVN[+2392]NSGEY.R z=3,scan#=7446,scan time=25.3893
V158 N64 HexNAc(5)Hex(4)DeoxyHex(1)Neu5Ac(2)
IAAARVNNSGEY
Intensity
m/z
C6H8NO2
C7H8NO2
HexNAc-18
HexNAc-36
HexNAc
NeuAc-18
NeuAc
HexNAcHex
HexHexNAcFuc
HexNAcHexNeuAc
Pep+HexNAc_2+
Pep+2HexNAc_2+
Pep+2HexNAc+Hex_2+
Pep+2HexNAc+2Hex_2+
Pep+2HexNAc+3Hex_2+
Pep+3HexNAc+3Hex_2+
Pep+3HexNAc+4Hex_2+
Pep_1+
Pep+HexNAc_1+
M_2+ - HexNAcHexNeuAc
M_2+ - NeuAc(2)
Pep+HexNAc+Fuc_1+
Pep+2HexNAc_1+
M_2+ - NeuAc
Pep+2HexNAc+Fuc_1+
Pep+2HexNAc+Hex_1+
Pep+2HexNAc+2Hex_1+
Pep+2HexNAc+3Hex_1+
Pep+3HexNAc+3Hex_1+
y1
b3
b4
y3
b5

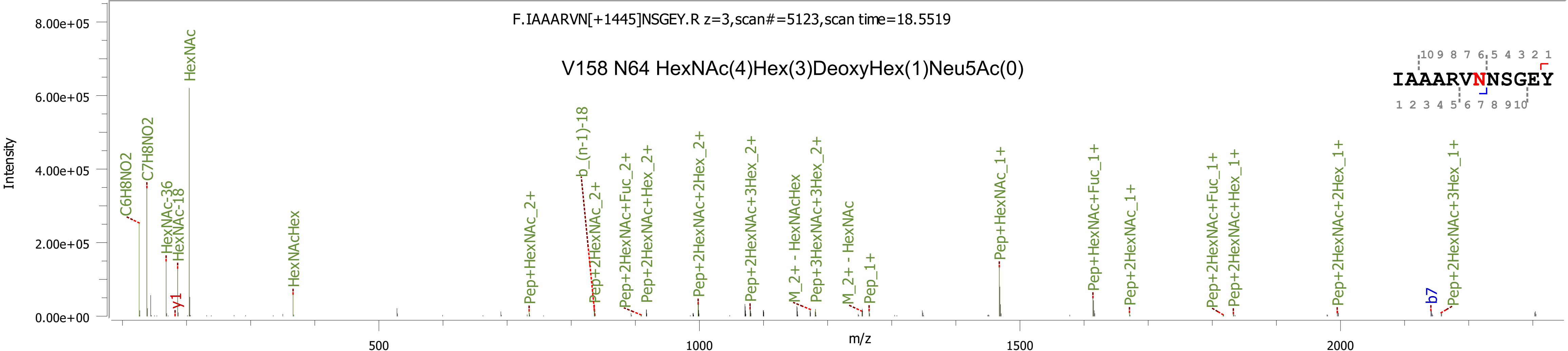

F.IAAARVN[+1445]NSGEY.R z=3,scan#=5123,scan time=18.5519
V158 N64 HexNAc(4)Hex(3)DeoxyHex(1)Neu5Ac(0)
IAAARVNNSGEY
Intensity
m/z
C6H8NO2
C7H8NO2
HexNAc-36
HexNAc-18
HexNAc
y1
HexNAcHex
Pep+HexNAc_2+
b_(n-1)-18
Pep+2HexNAc_2+
Pep+2HexNAc+Fuc_2+
Pep+2HexNAc+Hex_2+
Pep+2HexNAc+2Hex_2+
Pep+2HexNAc+3Hex_2+
M_2+ - HexNAcHex
Pep+3HexNAc+3Hex_2+
M_2+ - HexNAc
Pep_1+
Pep+HexNAc_1+
Pep+HexNAc+Fuc_1+
Pep+2HexNAc_1+
Pep+2HexNAc+Fuc_1+
Pep+2HexNAc+Hex_1+
Pep+2HexNAc+2Hex_1+
b7
Pep+2HexNAc+3Hex_1+

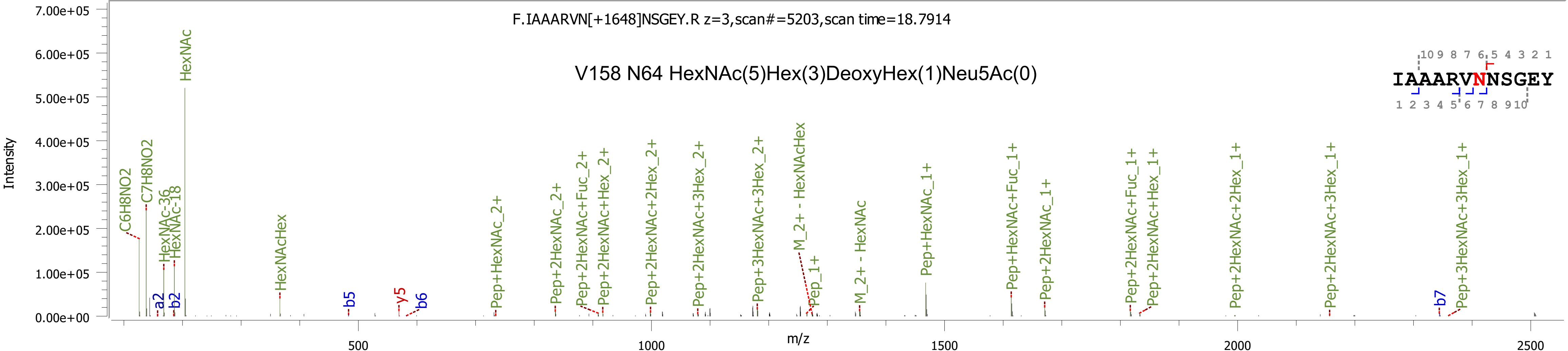

F.IAAARVN[+1648]NSGEY.R z=3,scan#=5203,scan time=18.7914
V158 N64 HexNAc(5)Hex(3)DeoxyHex(1)Neu5Ac(0)
IAAARVNNSGEY
Intensity
m/z
C6H8NO2
C7H8NO2
HexNAc-36
HexNAc-18
HexNAc
a2
b2
HexNAcHex
b5
y5
b6
Pep+HexNAc_2+
Pep+2HexNAc_2+
Pep+2HexNAc+Fuc_2+
Pep+2HexNAc+Hex_2+
Pep+2HexNAc+2Hex_2+
Pep+2HexNAc+3Hex_2+
Pep+3HexNAc+3Hex_2+
M_2+ - HexNAcHex
Pep_1+
M_2+ - HexNAc
Pep+HexNAc_1+
Pep+HexNAc+Fuc_1+
Pep+2HexNAc_1+
Pep+2HexNAc+Fuc_1+
Pep+2HexNAc+Hex_1+
Pep+2HexNAc+2Hex_1+
Pep+2HexNAc+3Hex_1+
b7
Pep+3HexNAc+3Hex_1+

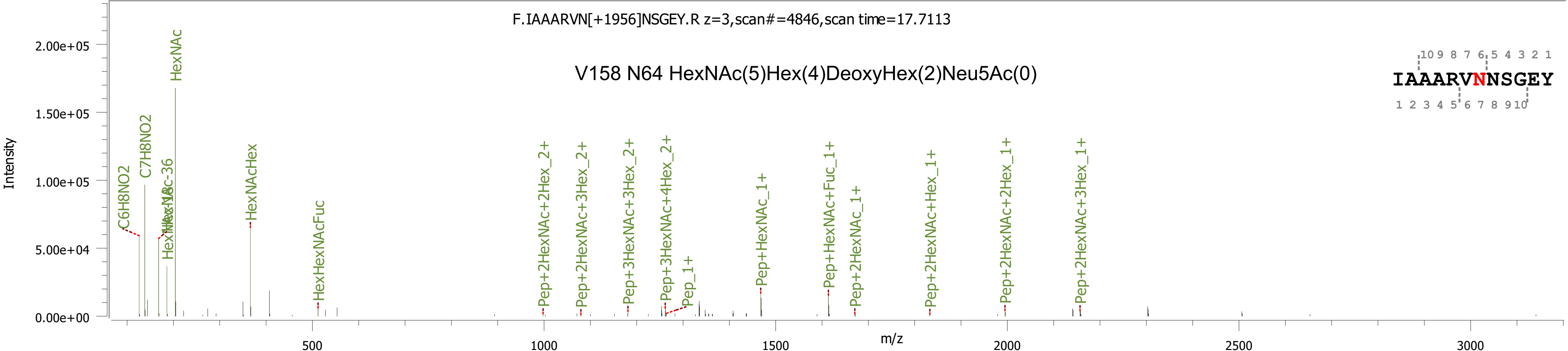
F.IAAARVN[+1956]NSGEY.R z=3,scan#=4846,scan time=17.7113
V158 N64 HexNAc(5)Hex(4)DeoxyHex(2)Neu5Ac(0)
IAAARVNNSGEY
Intensity
m/z
HexNAc
C7H8NO2
C6H8NO2
HexNAcHex
HexHexNAcFuc
Pep+2HexNAc+2Hex_2+
Pep+2HexNAc+3Hex_2+
Pep+3HexNAc+3Hex_2+
Pep+3HexNAc+4Hex_2+
Pep_1+
Pep+HexNAc_1+
Pep+HexNAc+Fuc_1+
Pep+2HexNAc_1+
Pep+2HexNAc+Hex_1+
Pep+2HexNAc+2Hex_1+
Pep+2HexNAc+3Hex_1+

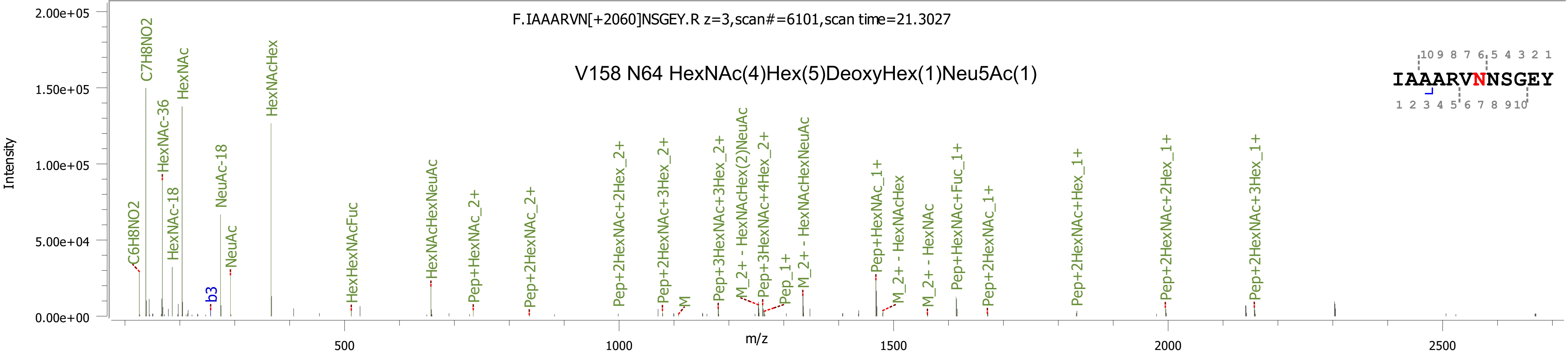

F.IAAARVN[+2060]NSGEY.R z=3,scan#=6101,scan time=21.3027
V158 N64 HexNAc(4)Hex(5)DeoxyHex(1)Neu5Ac(1)
IAAARVNNSGEY
Intensity
m/z
C6H8NO2
C7H8NO2
HexNAc-36
HexNAc-18
HexNAc
b3
NeuAc-18
NeuAc
HexNAcHex
HexHexNAcFuc
HexNAcHexNeuAc
Pep+HexNAc_2+
Pep+2HexNAc_2+
Pep+2HexNAc+2Hex_2+
Pep+2HexNAc+3Hex_2+
M
Pep+3HexNAc+3Hex_2+
M_2+ - HexNAcHex(2)NeuAc
Pep+3HexNAc+4Hex_2+
Pep_1+
M_2+ - HexNAcHexNeuAc
Pep+HexNAc_1+
M_2+ - HexNAcHex
M_2+ - HexNAc
Pep+HexNAc+Fuc_1+
Pep+2HexNAc_1+
Pep+2HexNAc+Hex_1+
Pep+2HexNAc+2Hex_1+
Pep+2HexNAc+3Hex_1+

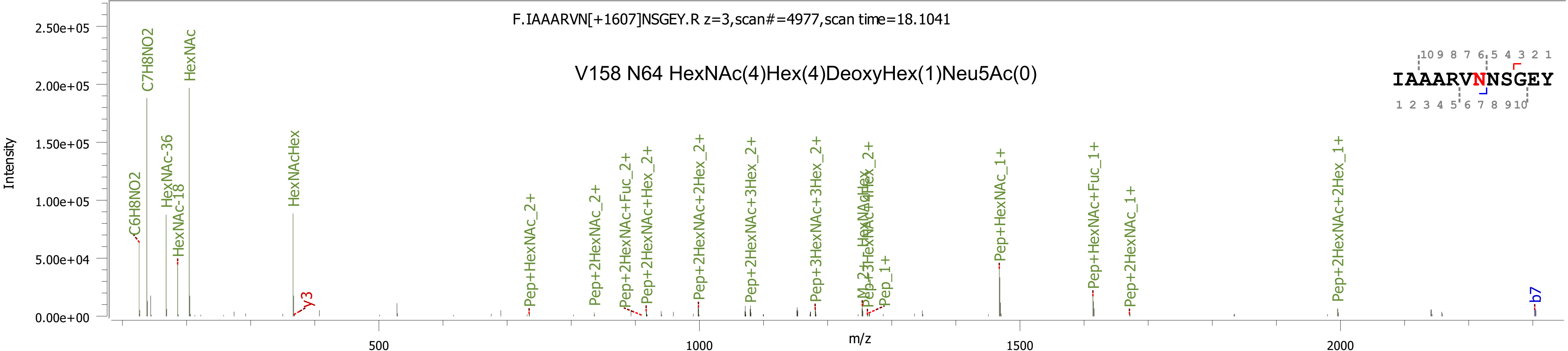

F.IAAARVN[+1607]NSGEY.R z=3,scan#=4977,scan time=18.1041
V158 N64 HexNAc(4)Hex(4)DeoxyHex(1)Neu5Ac(0)
IAAARVNNSGEY
Intensity
m/z
2.50e+05
2.00e+05
1.50e+05
1.00e+05
5.00e+04
0.00e+00
500
1000
1500
2000
C6H8NO2
C7H8NO2
HexNAc-36
HexNAc-18
HexNAc
HexNAcHex
y3
Pep+HexNAc_2+
Pep+2HexNAc_2+
Pep+2HexNAc+Fuc_2+
Pep+2HexNAc+Hex_2+
Pep+2HexNAc+2Hex_2+
Pep+2HexNAc+3Hex_2+
Pep+3HexNAc+3Hex_2+
Pep_1+
Pep+HexNAc_1+
Pep+HexNAc+Fuc_1+
Pep+2HexNAc_1+
Pep+2HexNAc+2Hex_1+
b7

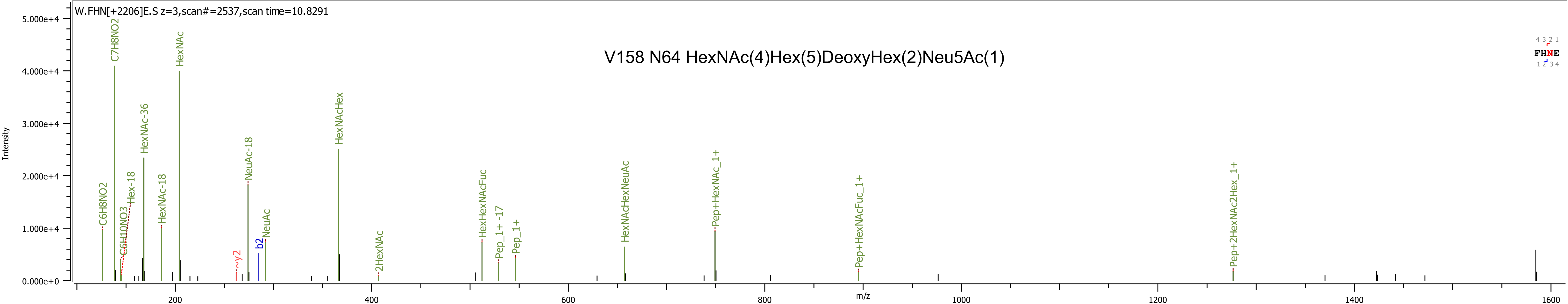
W.FHN[+2206]E.S z=3,scan#=2537,scan time=10.8291
V158 N64 HexNAc(4)Hex(5)DeoxyHex(2)Neu5Ac(1)
FHNE
Intensity
m/z
C6H8NO2
C7H8NO2
C6H10NO3
Hex-18
HexNAc-36
HexNAc-18
HexNAc
~y2
NeuAc-18
b2
NeuAc
HexNAcHex
2HexNAc
HexHexNAcFuc
Pep_1+ -17
Pep_1+
HexNAcHexNeuAc
Pep+HexNAc_1+
Pep+HexNAcFuc_1+
Pep+2HexNAc2Hex_1+

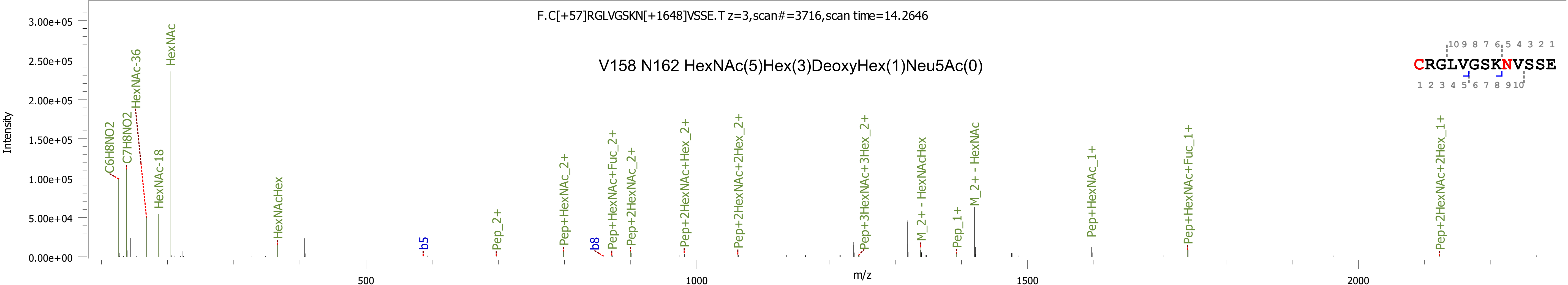
F.C[+57]RGLVGSKN[+1648]VSSE.T z=3,scan#=3716,scan time=14.2646
V158 N162 HexNAc(5)Hex(3)DeoxyHex(1)Neu5Ac(0)
CRGLVGSKNVSSE
Intensity
m/z
C6H8NO2
C7H8NO2
HexNAc-36
HexNAc-18
HexNAc
HexNAcHex
b5
Pep_2+
Pep+HexNAc_2+
b8
Pep+HexNAc+Fuc_2+
Pep+2HexNAc_2+
Pep+2HexNAc+Hex_2+
Pep+2HexNAc+2Hex_2+
Pep+3HexNAc+3Hex_2+
M_2+ - HexNAcHex
Pep_1+
M_2+ - HexNAc
Pep+HexNAc_1+
Pep+HexNAc+Fuc_1+
Pep+2HexNAc+2Hex_1+

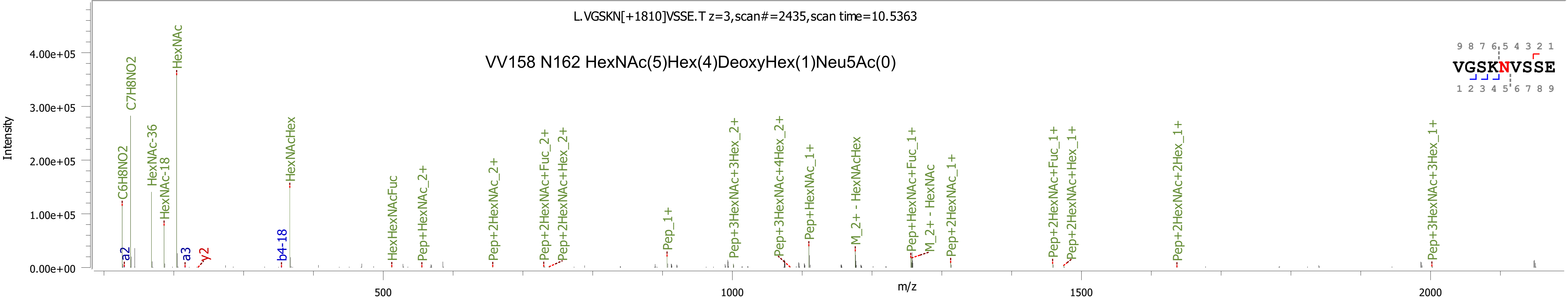

L.VGSKN[+1810]VSSE.T z=3,scan#=2435,scan time=10.5363
VV158 N162 HexNAc(5)Hex(4)DeoxyHex(1)Neu5Ac(0)
VGSKNVSSE
Intensity
m/z
4.00e+05
3.00e+05
2.00e+05
1.00e+05
0.00e+00
500
1000
1500
2000
C6H8NO2
C7H8NO2
HexNAc-36
HexNAc-18
HexNAc
a2
a3
y2
b4-18
HexNAcHex
HexHexNAcFuc
Pep+HexNAc_2+
Pep+2HexNAc_2+
Pep+2HexNAc+Fuc_2+
Pep+2HexNAc+Hex_2+
Pep_1+
Pep+3HexNAc+3Hex_2+
Pep+3HexNAc+4Hex_2+
Pep+HexNAc_1+
M_2+ - HexNAcHex
Pep+HexNAc+Fuc_1+
M_2+ - HexNAc
Pep+2HexNAc_1+
Pep+2HexNAc+Fuc_1+
Pep+2HexNAc+Hex_1+
Pep+2HexNAc+2Hex_1+
Pep+3HexNAc+3Hex_1+

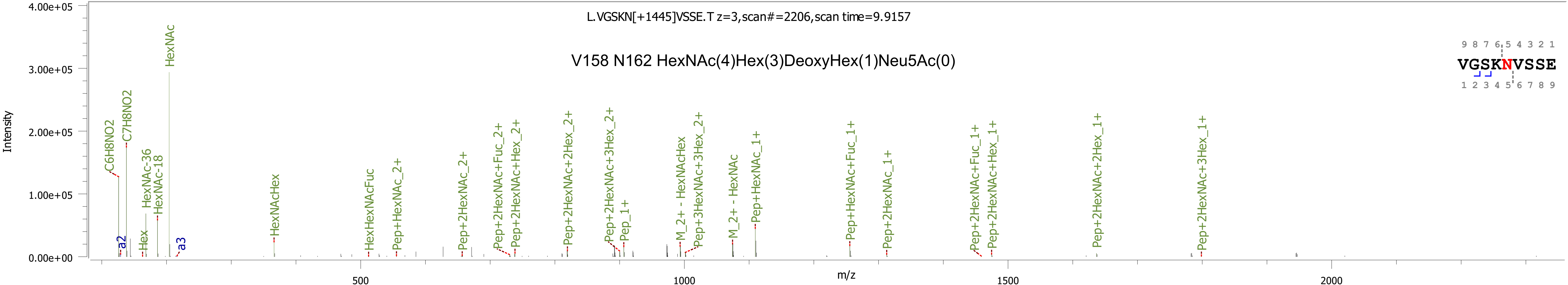

L.VGSKN[+1445]VSSE.T z=3,scan#=2206,scan time=9.9157
V158 N162 HexNAc(4)Hex(3)DeoxyHex(1)Neu5Ac(0)
VGSKNVSSE
Intensity
m/z
C6H8NO2
C7H8NO2
a2
Hex
HexNAc-36
HexNAc-18
HexNAc
a3
HexNAcHex
HexHexNAcFuc
Pep+HexNAc_2+
Pep+2HexNAc_2+
Pep+2HexNAc+Fuc_2+
Pep+2HexNAc+Hex_2+
Pep+2HexNAc+2Hex_2+
Pep+2HexNAc+3Hex_2+
Pep_1+
M_2+ - HexNAcHex
Pep+3HexNAc+3Hex_2+
M_2+ - HexNAc
Pep+HexNAc_1+
Pep+HexNAc+Fuc_1+
Pep+2HexNAc_1+
Pep+2HexNAc+Fuc_1+
Pep+2HexNAc+Hex_1+
Pep+2HexNAc+2Hex_1+
Pep+2HexNAc+3Hex_1+

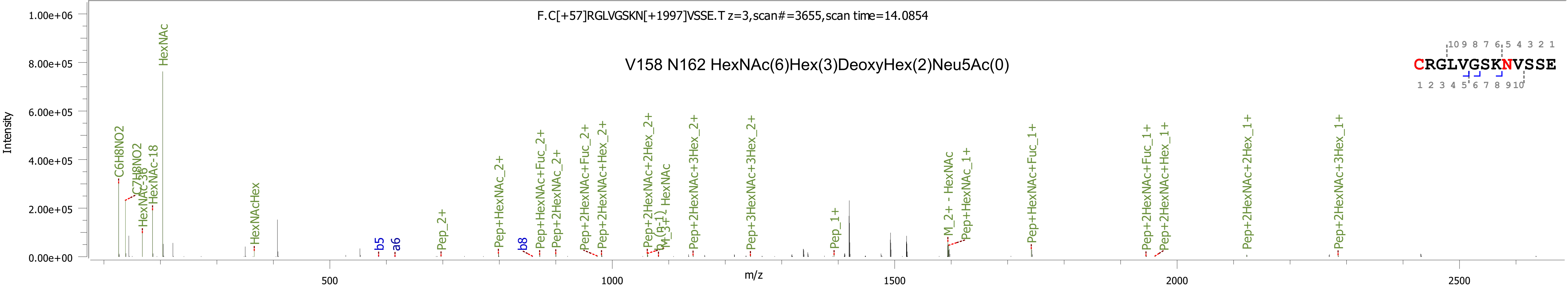

F.C[+57]RGLVGSKN[+1997]VSSE.T z=3,scan#=3655,scan time=14.0854
V158 N162 HexNAc(6)Hex(3)DeoxyHex(2)Neu5Ac(0)
CRGLVGSKNVSSE
Intensity
m/z
C6H8NO2
C7H8NO2
HexNAc-36
HexNAc-18
HexNAc
HexNAcHex
b5
a6
Pep_2+
Pep+HexNAc_2+
b8
Pep+HexNAc+Fuc_2+
Pep+2HexNAc_2+
Pep+2HexNAc+Fuc_2+
Pep+2HexNAc+Hex_2+
Pep+2HexNAc+2Hex_2+
Pep+2HexNAc+3Hex_2+
Pep+3HexNAc+3Hex_2+
Pep_1+
M_2+ - HexNAc
Pep+HexNAc_1+
Pep+HexNAc+Fuc_1+
Pep+2HexNAc+Fuc_1+
Pep+2HexNAc+Hex_1+
Pep+2HexNAc+2Hex_1+
Pep+2HexNAc+3Hex_1+

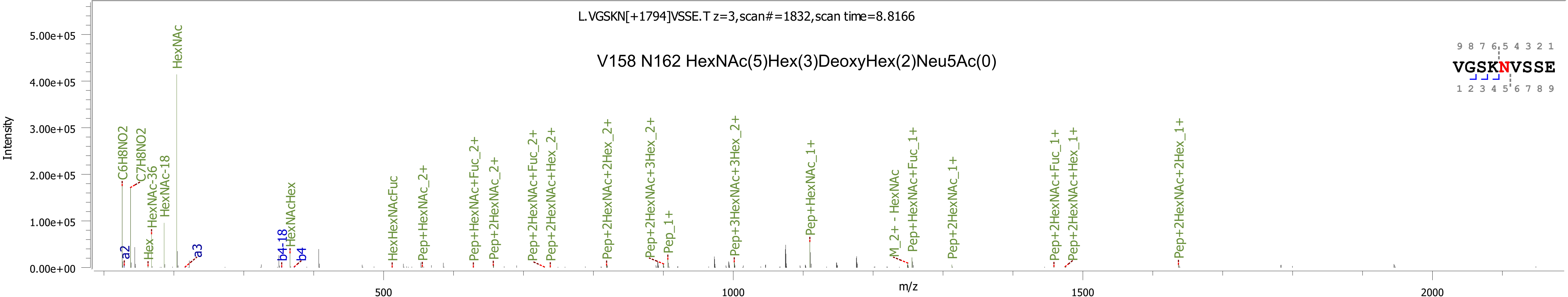

L.VGSKN[+1794]VSSE.T z=3,scan#=1832,scan time=8.8166
V158 N162 HexNAc(5)Hex(3)DeoxyHex(2)Neu5Ac(0)
VGSKNVSSE
Intensity
m/z
C6H8NO2
C7H8NO2
HexNAc-36
HexNAc-18
HexNAc
Hex
a2
a3
b4-18
HexNAcHex
b4
HexHexNAcFuc
Pep+HexNAc_2+
Pep+HexNAc+Fuc_2+
Pep+2HexNAc_2+
Pep+2HexNAc+Fuc_2+
Pep+2HexNAc+Hex_2+
Pep+2HexNAc+2Hex_2+
Pep+2HexNAc+3Hex_2+
Pep_1+
Pep+3HexNAc+3Hex_2+
Pep+HexNAc_1+
M_2+ - HexNAc
Pep+HexNAc+Fuc_1+
Pep+2HexNAc_1+
Pep+2HexNAc+Fuc_1+
Pep+2HexNAc+Hex_1+
Pep+2HexNAc+2Hex_1+

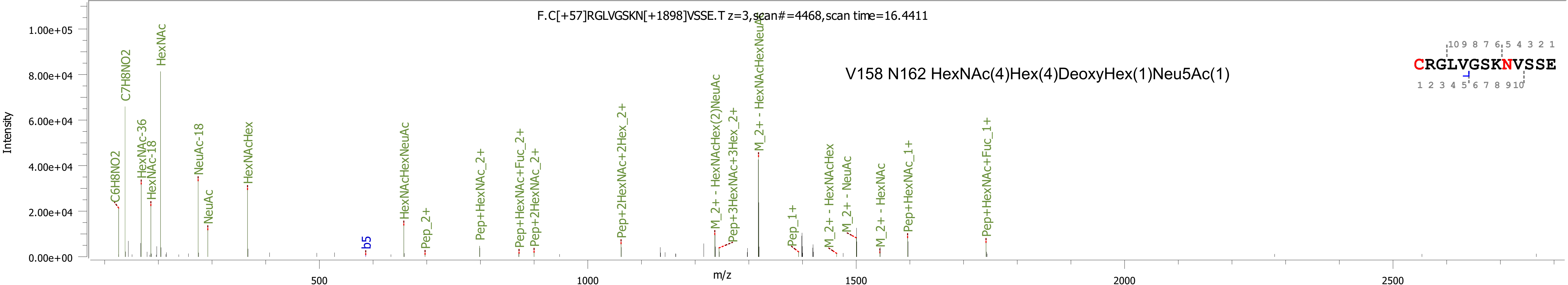

F.C[+57]RGLVGSKN[+1898]VSSE.T z=3,scan#=4468,scan time=16.4411
V158 N162 HexNAc(4)Hex(4)DeoxyHex(1)Neu5Ac(1)
CRGLVGSKNVSSE
Intensity
m/z
C6H8NO2
C7H8NO2
HexNAc-36
HexNAc-18
HexNAc
NeuAc-18
NeuAc
HexNAcHex
b5
HexNAcHexNeuAc
Pep_2+
Pep+HexNAc_2+
Pep+HexNAc+Fuc_2+
Pep+2HexNAc_2+
Pep+2HexNAc+2Hex_2+
M_2+ - HexNAcHex(2)NeuAc
Pep+3HexNAc+3Hex_2+
M_2+ - HexNAcHexNeuAc
Pep_1+
M_2+ - HexNAcHex
M_2+ - NeuAc
M_2+ - HexNAc
Pep+HexNAc_1+
Pep+HexNAc+Fuc_1+

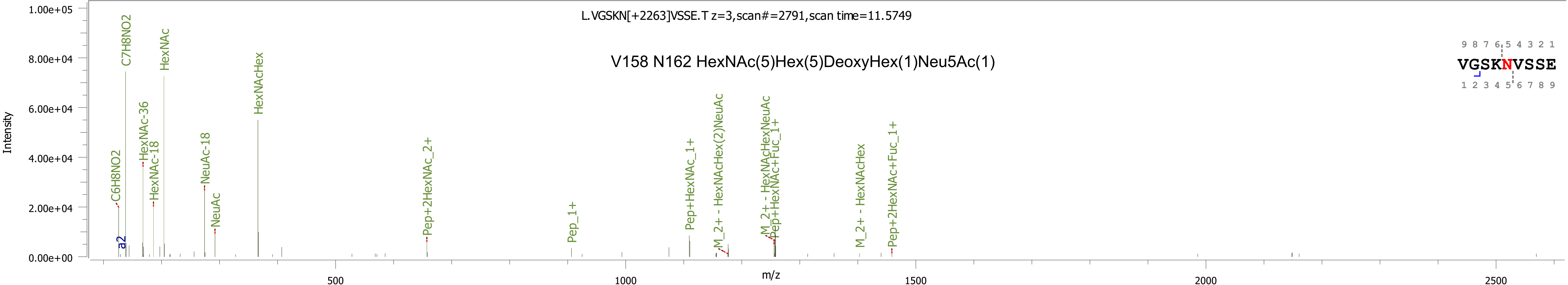
L.VGSKN[+2263]VSSE.T z=3,scan#=2791,scan time=11.5749
V158 N162 HexNAc(5)Hex(5)DeoxyHex(1)Neu5Ac(1)
VGSKNVSSE
Intensity
m/z
C7H8NO2
HexNAc
HexNAcHex
C6H8NO2
HexNAc-36
HexNAc-18
NeuAc-18
NeuAc
a2
Pep+2HexNAc_2+
Pep_1+
Pep+HexNAc_1+
M_2+ - HexNAcHex(2)NeuAc
M_2+ - HexNAcHexNeuAc
Pep+HexNAc+Fuc_1+
M_2+ - HexNAcHex
Pep+2HexNAc+Fuc_1+

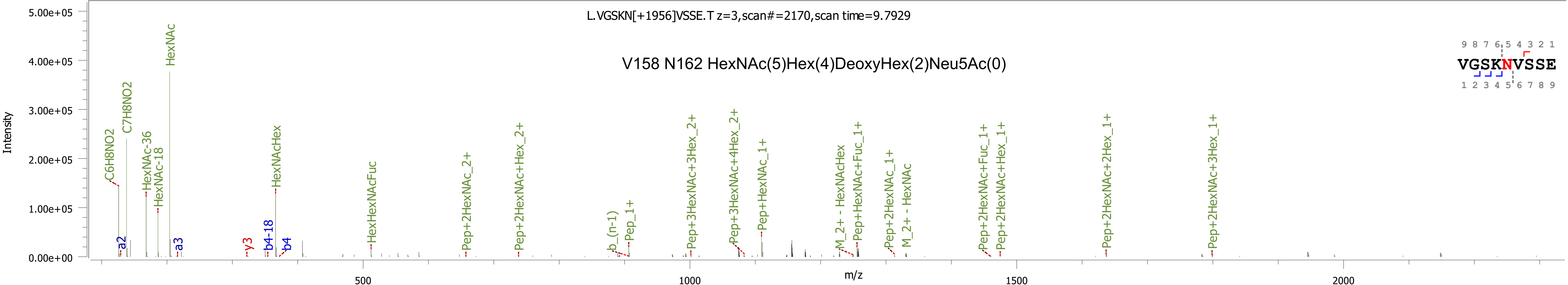

L.VGSKN[+1956]VSSE.T z=3,scan#=2170,scan time=9.7929
V158 N162 HexNAc(5)Hex(4)DeoxyHex(2)Neu5Ac(0)
VGSKNVSSE
Intensity
m/z
C6H8NO2
C7H8NO2
HexNAc-36
HexNAc-18
HexNAc
a2
a3
y3
b4-18
b4
HexNAcHex
HexHexNAcFuc
Pep+2HexNAc_2+
Pep+2HexNAc+Hex_2+
b_(n-1)
Pep_1+
Pep+3HexNAc+3Hex_2+
Pep+3HexNAc+4Hex_2+
Pep+HexNAc_1+
M_2+ - HexNAcHex
Pep+HexNAc+Fuc_1+
Pep+2HexNAc_1+
M_2+ - HexNAc
Pep+2HexNAc+Fuc_1+
Pep+2HexNAc+Hex_1+
Pep+2HexNAc+2Hex_1+
Pep+2HexNAc+3Hex_1+

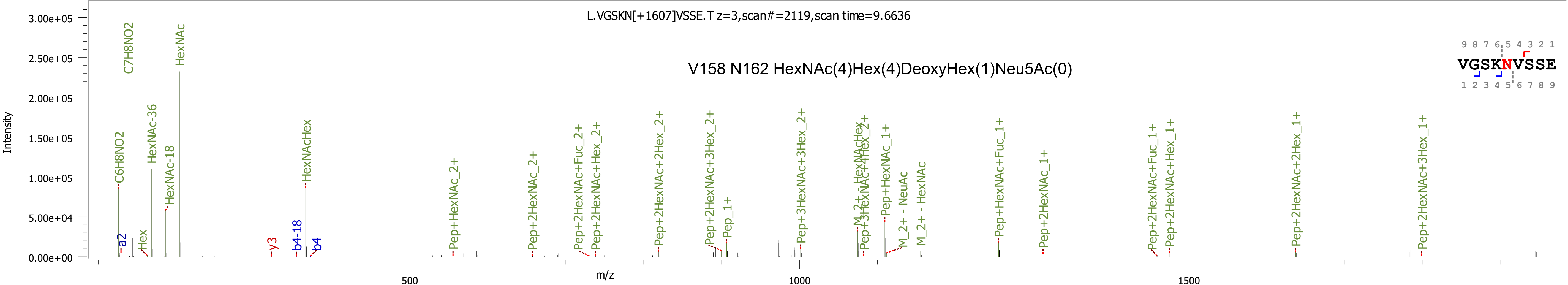

L.VGSKN[+1607]VSSE.T z=3,scan#=2119,scan time=9.6636
V158 N162 HexNAc(4)Hex(4)DeoxyHex(1)Neu5Ac(0)
9 8 7 6 5 4 3 2 1
VGSKNVSSE
1 2 3 4 5 6 7 8 9
Intensity
3.00e+05
2.50e+05
2.00e+05
1.50e+05
1.00e+05
5.00e+04
0.00e+00
m/z
500
1000
1500
C6H8NO2
C7H8NO2
a2
Hex
HexNAc-36
HexNAc-18
HexNAc
y3
b4-18
b4
HexNAcHex
Pep+HexNAc_2+
Pep+2HexNAc_2+
Pep+2HexNAc+Fuc_2+
Pep+2HexNAc+Hex_2+
Pep+2HexNAc+2Hex_2+
Pep+2HexNAc+3Hex_2+
Pep_1+
Pep+3HexNAc+3Hex_2+
M_2+ - HexNAcHex
Pep+3HexNAc+4Hex_2+
Pep+HexNAc_1+
M_2+ - NeuAc
M_2+ - HexNAc
Pep+HexNAc+Fuc_1+
Pep+2HexNAc_1+
Pep+2HexNAc+Fuc_1+
Pep+2HexNAc+Hex_1+
Pep+2HexNAc+2Hex_1+
Pep+2HexNAc+3Hex_1+

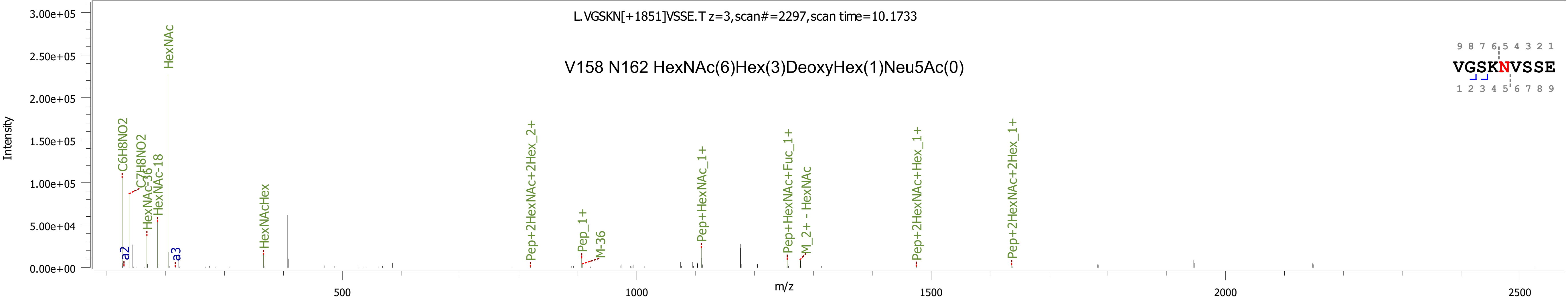

L.VGSKN[+1851]VSSE.T z=3,scan#=2297,scan time=10.1733
V158 N162 HexNAc(6)Hex(3)DeoxyHex(1)Neu5Ac(0)
VGSKNVSSE
9 8 7 6 5 4 3 2 1
1 2 3 4 5 6 7 8 9
Intensity
m/z
3.00e+05
2.50e+05
2.00e+05
1.50e+05
1.00e+05
5.00e+04
0.00e+00
500
1000
1500
2000
2500
a2
C6H8NO2
C7H8NO2
HexNAc-36
HexNAc-18
HexNAc
a3
HexNAcHex
Pep+2HexNAc+2Hex_2+
Pep_1+
M-36
Pep+HexNAc_1+
Pep+HexNAc+Fuc_1+
M_2+ - HexNAc
Pep+2HexNAc+Hex_1+
Pep+2HexNAc+2Hex_1+

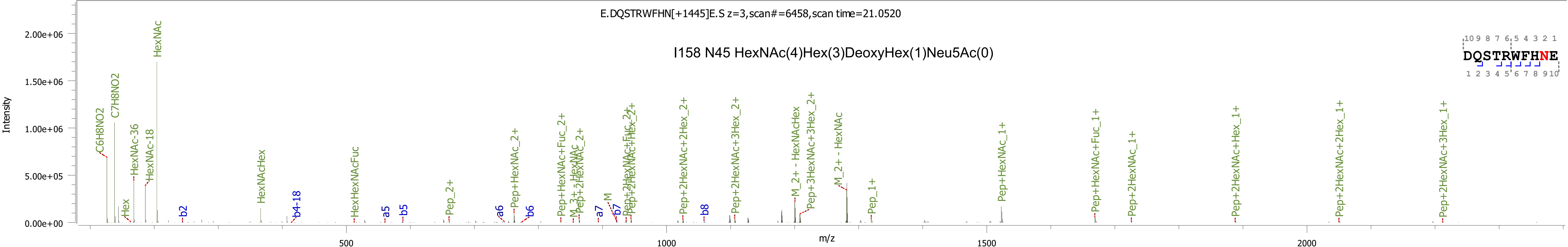
E.DQSTRWFHN[+1445]E.S z=3,scan#=6458,scan time=21.0520
I158 N45 HexNAc(4)Hex(3)DeoxyHex(1)Neu5Ac(0)
DQSTRWFHNE
Intensity
m/z
2.00e+06
1.50e+06
1.00e+06
5.00e+05
0.00e+00
500
1000
1500
2000
C6H8NO2
C7H8NO2
Hex
HexNAc-36
HexNAc-18
HexNAc
b2
HexNAcHex
b4-18
HexHexNAcFuc
a5
b5
Pep_2+
a6
Pep+HexNAc_2+
b6
Pep+HexNAc+Fuc_2+
a7
M
b7
b8
Pep+2HexNAc+2Hex_2+
Pep+2HexNAc+3Hex_2+
M_2+ - HexNAcHex
Pep+3HexNAc+3Hex_2+
M_2+ - HexNAc
Pep_1+
Pep+HexNAc_1+
Pep+HexNAc+Fuc_1+
Pep+2HexNAc_1+
Pep+2HexNAc+Hex_1+
Pep+2HexNAc+2Hex_1+
Pep+2HexNAc+3Hex_1+

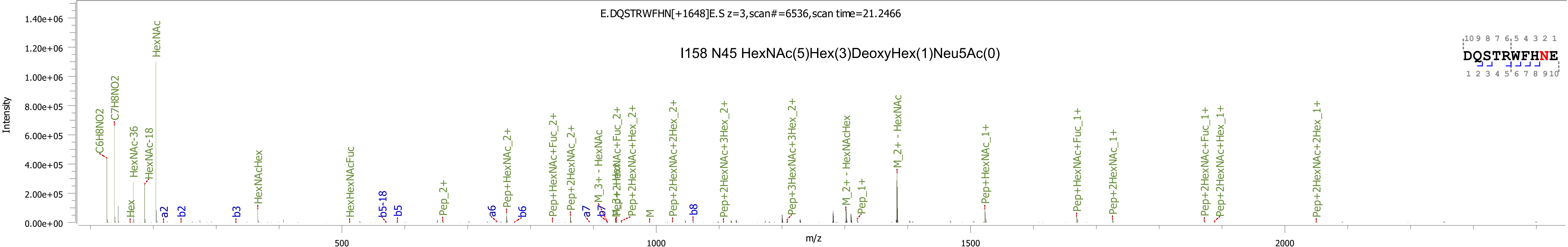

E.DQSTRWFHN[+1648]E.S z=3,scan#=6536,scan time=21.2466
I158 N45 HexNAc(5)Hex(3)DeoxyHex(1)Neu5Ac(0)
DQSTRWFHNE
Intensity
m/z
C6H8NO2
C7H8NO2
Hex
HexNAc-36
HexNAc-18
HexNAc
a2
b2
b3
HexNAcHex
HexHexNAcFuc
b5-18
b5
Pep_2+
a6
Pep+HexNAc_2+
b6
Pep+HexNAc+Fuc_2+
Pep+2HexNAc_2+
a7
M_3+ - HexNAc
b7
Pep+2HexNAc+Fuc_2+
Pep+2HexNAc+Hex_2+
M
Pep+2HexNAc+2Hex_2+
b8
Pep+2HexNAc+3Hex_2+
Pep+3HexNAc+3Hex_2+
M_2+ - HexNAcHex
Pep_1+
M_2+ - HexNAc
Pep+HexNAc_1+
Pep+HexNAc+Fuc_1+
Pep+2HexNAc_1+
Pep+2HexNAc+Fuc_1+
Pep+2HexNAc+Hex_1+
Pep+2HexNAc+2Hex_1+

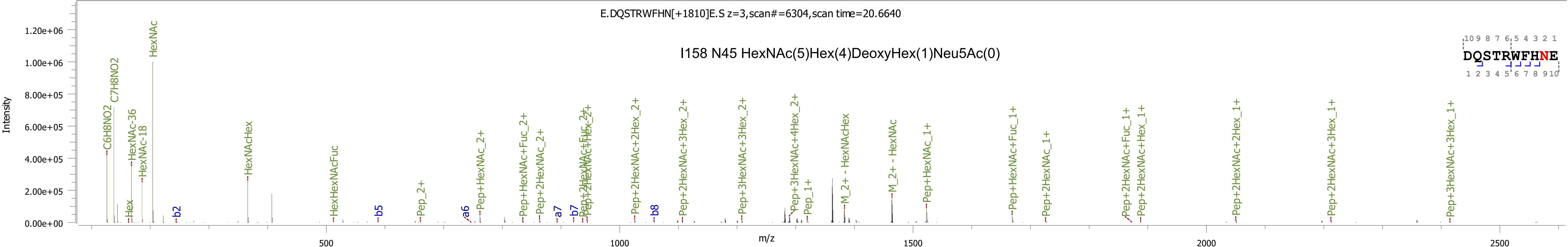
E.DQSTRWFHN[+1810]E.S z=3,scan#=6304,scan time=20.6640
I158 N45 HexNAc(5)Hex(4)DeoxyHex(1)Neu5Ac(0)
DQSTRWFHNE
Intensity
m/z
C6H8NO2
C7H8NO2
Hex
HexNAc-36
HexNAc-18
HexNAc
b2
HexNAcHex
HexHexNAcFuc
b5
Pep_2+
a6
Pep+HexNAc_2+
Pep+HexNAc+Fuc_2+
Pep+2HexNAc_2+
a7
b7
Pep+2HexNAc+Fuc_2+
Pep+2HexNAc+Hex_2+
Pep+2HexNAc+2Hex_2+
b8
Pep+2HexNAc+3Hex_2+
Pep+3HexNAc+3Hex_2+
Pep+3HexNAc+4Hex_2+
Pep_1+
M_2+ - HexNAcHex
M_2+ - HexNAc
Pep+HexNAc_1+
Pep+HexNAc+Fuc_1+
Pep+2HexNAc_1+
Pep+2HexNAc+Fuc_1+
Pep+2HexNAc+Hex_1+
Pep+2HexNAc+2Hex_1+
Pep+2HexNAc+3Hex_1+
Pep+3HexNAc+3Hex_1+

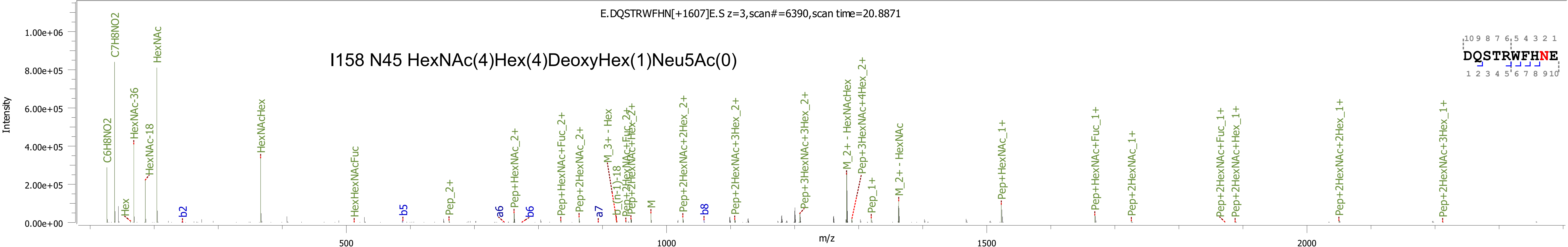

E.DQSTRWFHN[+1607]E.S z=3,scan#=6390,scan time=20.8871
I158 N45 HexNAc(4)Hex(4)DeoxyHex(1)Neu5Ac(0)
DQSTRWFHNE
Intensity
m/z
C6H8NO2
C7H8NO2
Hex
HexNAc-36
HexNAc-18
HexNAc
b2
HexNAcHex
HexNAcFuc
b5
Pep_2+
a6
Pep+HexNAc_2+
b6
Pep+HexNAc+Fuc_2+
Pep+2HexNAc_2+
a7
M_3+ - Hex
M
Pep+2HexNAc+2Hex_2+
b8
Pep+2HexNAc+3Hex_2+
Pep+3HexNAc+3Hex_2+
M_2+ - HexNAcHex
Pep+3HexNAc+4Hex_2+
Pep_1+
M_2+ - HexNAc
Pep+HexNAc_1+
Pep+HexNAc+Fuc_1+
Pep+2HexNAc_1+
Pep+2HexNAc+Fuc_1+
Pep+2HexNAc+Hex_1+
Pep+2HexNAc+2Hex_1+
Pep+2HexNAc+3Hex_1+

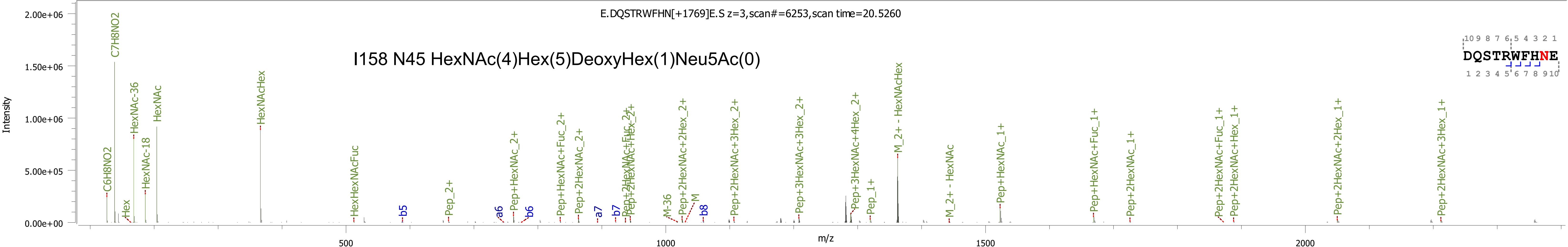

E.DQSTRWFHN[+1769]E.S z=3,scan#=6253,scan time=20.5260
I158 N45 HexNAc(4)Hex(5)DeoxyHex(1)Neu5Ac(0)
DQSTRWFHNE
Intensity
m/z
2.00e+06
1.50e+06
1.00e+06
5.00e+05
0.00e+00
500
1000
1500
2000
C7H8NO2
C6H8NO2
Hex
HexNAc-36
HexNAc-18
HexNAc
HexNAcHex
HexHexNAcFuc
b5
Pep_2+
a6
Pep+HexNAc_2+
b6
Pep+HexNAc+Fuc_2+
Pep+2HexNAc_2+
a7
b7
M-36
Pep+2HexNAc+2Hex_2+
M
b8
Pep+2HexNAc+3Hex_2+
Pep+3HexNAc+3Hex_2+
Pep+3HexNAc+4Hex_2+
Pep_1+
M_2+ - HexNAcHex
M_2+ - HexNAc
Pep+HexNAc_1+
Pep+HexNAc+Fuc_1+
Pep+2HexNAc_1+
Pep+2HexNAc+Fuc_1+
Pep+2HexNAc+Hex_1+
Pep+2HexNAc+2Hex_1+
Pep+2HexNAc+3Hex_1+

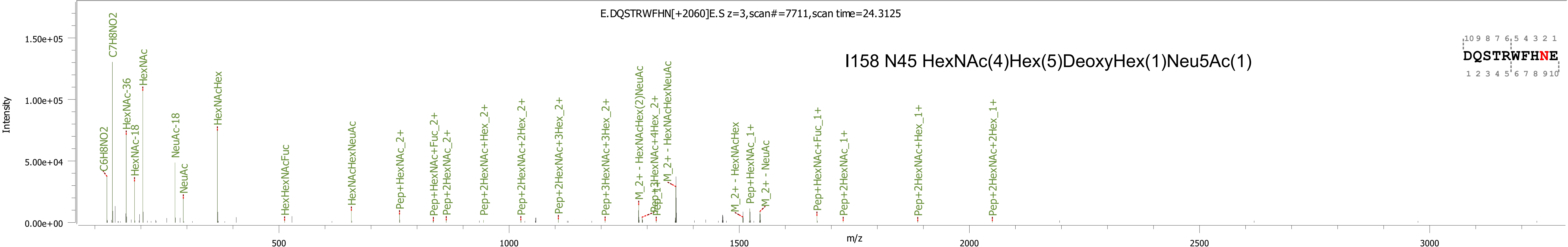
E.DQSTRWFHN[+2060]E.S z=3,scan#=7711,scan time=24.3125
I158 N45 HexNAc(4)Hex(5)DeoxyHex(1)Neu5Ac(1)
DQSTRWFHNE
Intensity
m/z
C6H8NO2
C7H8NO2
HexNAc-36
HexNAc-18
HexNAc
NeuAc-18
NeuAc
HexNAcHex
HexHexNAcFuc
HexNAcHexNeuAc
Pep+HexNAc_2+
Pep+HexNAc+Fuc_2+
Pep+2HexNAc_2+
Pep+2HexNAc+Hex_2+
Pep+2HexNAc+2Hex_2+
Pep+2HexNAc+3Hex_2+
Pep+3HexNAc+3Hex_2+
M_2+ - HexNAcHex(2)NeuAc
Pep+3HexNAc+4Hex_2+
M_2+ - HexNAcHexNeuAc
M_2+ - HexNAcHex
Pep+HexNAc_1+
M_2+ - NeuAc
Pep+HexNAc+Fuc_1+
Pep+2HexNAc_1+
Pep+2HexNAc+Hex_1+
Pep+2HexNAc+2Hex_1+

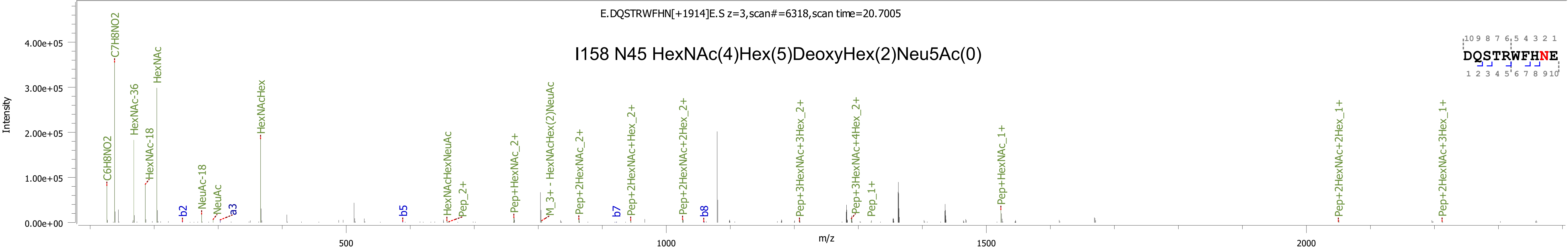

E.DQSTRWFHN[+1914]E.S z=3,scan#=6318,scan time=20.7005
I158 N45 HexNAc(4)Hex(5)DeoxyHex(2)Neu5Ac(0)
DQSTRWFHNE
Intensity
m/z
C7H8NO2
C6H8NO2
HexNAc-36
HexNAc-18
HexNAc
HexNAcHex
NeuAc-18
NeuAc
b2
a3
b5
HexNAcHexNeuAc
Pep_2+
Pep+HexNAc_2+
M_3+ - HexNAcHex(2)NeuAc
Pep+2HexNAc_2+
b7
Pep+2HexNAc+Hex_2+
Pep+2HexNAc+2Hex_2+
b8
Pep+3HexNAc+3Hex_2+
Pep+3HexNAc+4Hex_2+
Pep_1+
Pep+HexNAc_1+
Pep+2HexNAc+2Hex_1+
Pep+2HexNAc+3Hex_1+

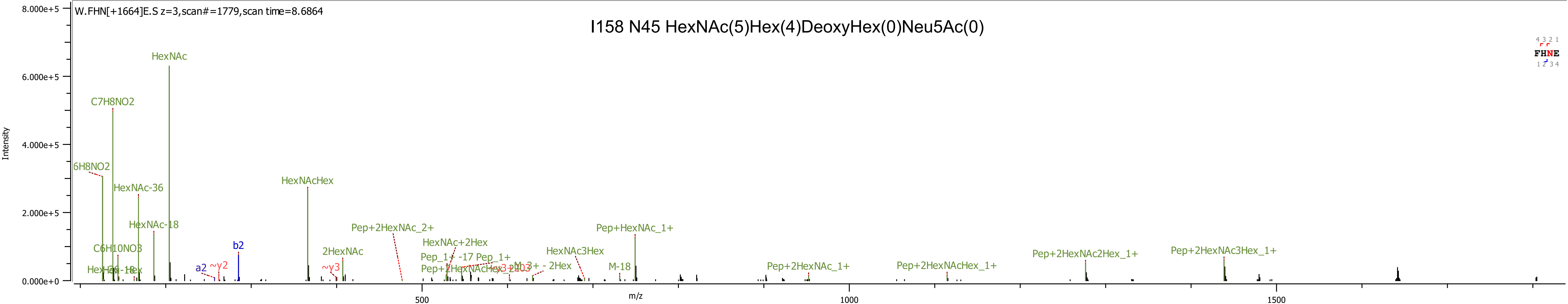

W.FHN[+1664]E.S z=3,scan#=1779,scan time=8.6864
I158 N45 HexNAc(5)Hex(4)DeoxyHex(0)Neu5Ac(0)
FHNE
Intensity
8.000e+5
6.000e+5
4.000e+5
2.000e+5
0.000e+0
m/z
500
1000
1500
HexNAc
C7H8NO2
6H8NO2
HexNAc-36
HexNAc-18
C6H10NO3
HexNAcHex
b2
a2
~y2
~y3
2HexNAc
Pep+2HexNAc_2+
HexNAc+2Hex
HexNAc3Hex
Pep+HexNAc_1+
M-18
Pep+2HexNAc_1+
Pep+2HexNAcHex_1+
Pep+2HexNAc2Hex_1+
Pep+2HexNAc3Hex_1+

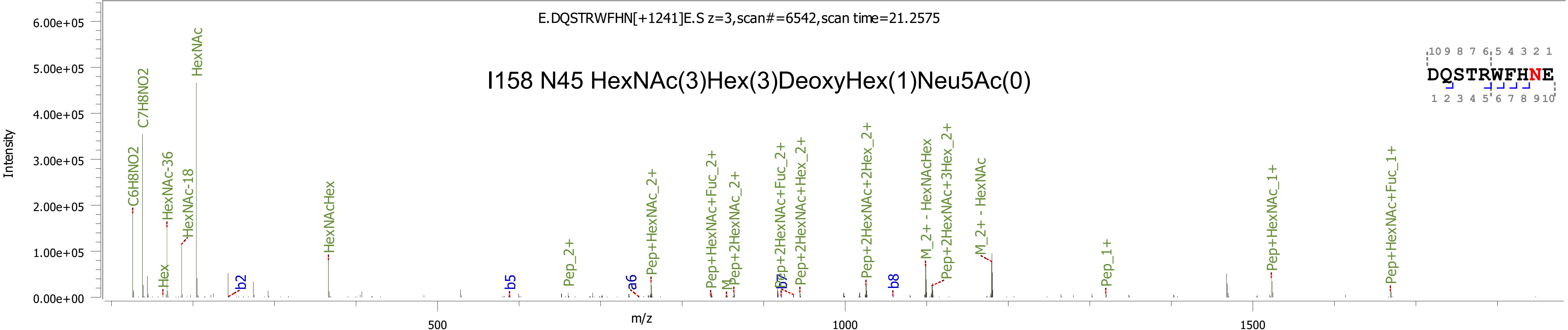
E.DQSTRWFHN[+1241]E.S z=3,scan#=6542,scan time=21.2575
I158 N45 HexNAc(3)Hex(3)DeoxyHex(1)Neu5Ac(0)
DQSTRWFHNE
Intensity
m/z
C6H8NO2
C7H8NO2
Hex
HexNAc-36
HexNAc-18
HexNAc
b2
HexNAcHex
b5
Pep_2+
a6
Pep+HexNAc_2+
Pep+HexNAc+Fuc_2+
M
Pep+2HexNAc_2+
b7
Pep+2HexNAc+Fuc_2+
Pep+2HexNAc+Hex_2+
Pep+2HexNAc+2Hex_2+
b8
M_2+ - HexNAcHex
Pep+2HexNAc+3Hex_2+
M_2+ - HexNAc
Pep_1+
Pep+HexNAc_1+
Pep+HexNAc+Fuc_1+

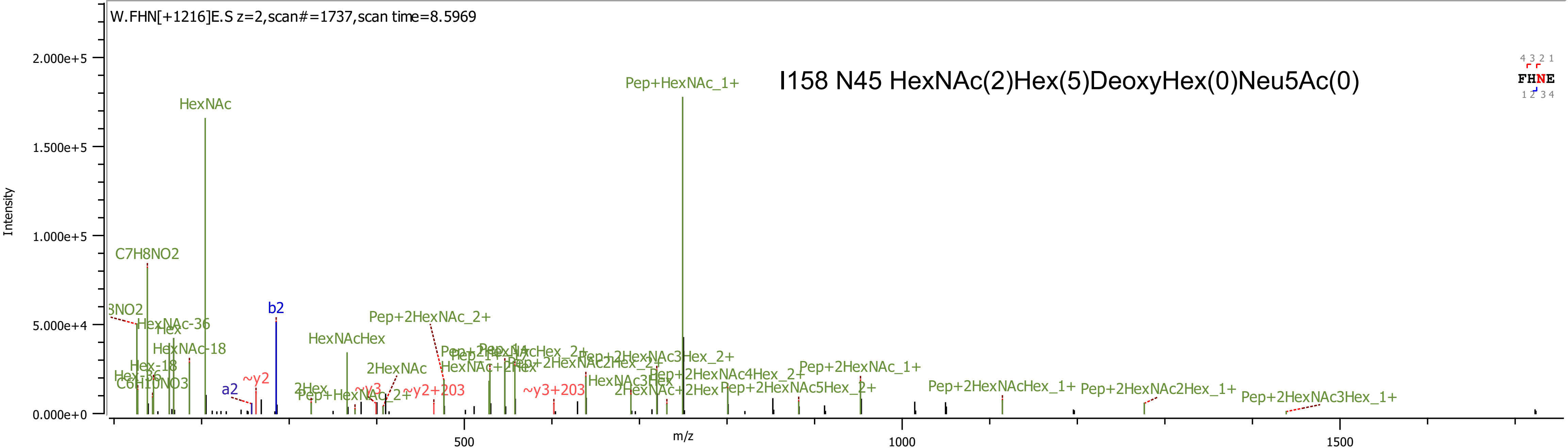
W.FHN[+1216]E.S z=2,scan#=1737,scan time=8.5969
I158 N45 HexNAc(2)Hex(5)DeoxyHex(0)Neu5Ac(0)
FHNE
Intensity
m/z
2.000e+5
1.500e+5
1.000e+5
5.000e+4
0.000e+0
500
1000
1500
HexNAc
Pep+HexNAc_1+
C7H8NO2
b2
a2
~y2
Pep+2HexNAc_2+
HexNAcHex
HexNAc-36
HexNAc-18
Hex-18
C6H10NO3
2HexNAc
2Hex
~y3
~y2+203
~y3+203
Pep+2HexNAc3Hex_2+
Pep+2HexNAc4Hex_2+
Pep+2HexNAc5Hex_2+
Pep+2HexNAc_1+
Pep+2HexNAcHex_1+
Pep+2HexNAc2Hex_1+
Pep+2HexNAc3Hex_1+
2HexNAc+2Hex

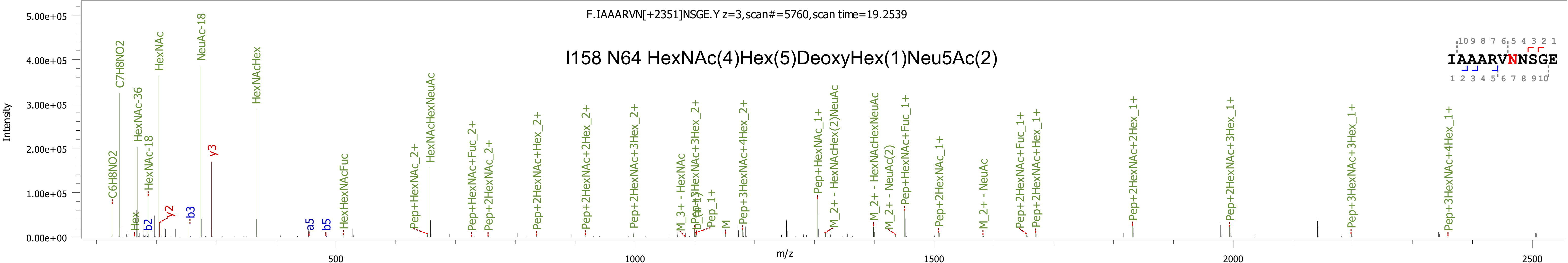
F.IAAARVN[+2351]NSGE.Y z=3,scan#=5760,scan time=19.2539
I158 N64 HexNAc(4)Hex(5)DeoxyHex(1)Neu5Ac(2)
IAAARVNNSGE
10 9 8 7 6 5 4 3 2 1
1 2 3 4 5 6 7 8 9 10
Intensity
5.00e+05
4.00e+05
3.00e+05
2.00e+05
1.00e+05
0.00e+00
m/z
500
1000
1500
2000
2500
C6H8NO2
C7H8NO2
Hex
b2
HexNAc-36
HexNAc-18
HexNAc
y2
b3
NeuAc-18
y3
HexNAcHex
a5
b5
HexHexNAcFuc
Pep+HexNAc_2+
HexNAcHexNeuAc
Pep+HexNAc+Fuc_2+
Pep+2HexNAc_2+
Pep+2HexNAc+Hex_2+
Pep+2HexNAc+2Hex_2+
Pep+2HexNAc+3Hex_2+
M_3+ - HexNAc
Pep+3HexNAc+3Hex_2+
Pep_1+
M
Pep+3HexNAc+4Hex_2+
Pep+HexNAc_1+
M_2+ - HexNAcHex(2)NeuAc
M_2+ - HexNAcHexNeuAc
M_2+ - NeuAc(2)
Pep+HexNAc+Fuc_1+
Pep+2HexNAc_1+
M_2+ - NeuAc
Pep+2HexNAc+Fuc_1+
Pep+2HexNAc+Hex_1+
Pep+2HexNAc+2Hex_1+
Pep+2HexNAc+3Hex_1+
Pep+3HexNAc+3Hex_1+
Pep+3HexNAc+4Hex_1+

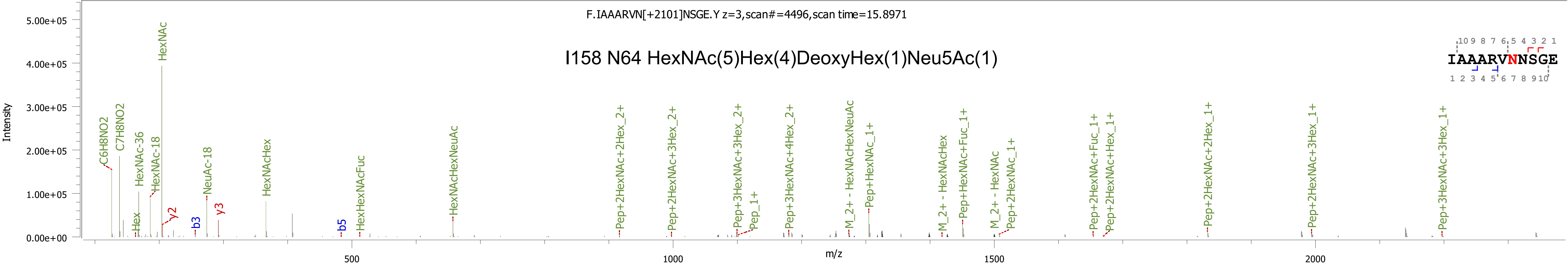

F.IAAARVN[+2101]NSGE.Y z=3,scan#=4496,scan time=15.8971
I158 N64 HexNAc(5)Hex(4)DeoxyHex(1)Neu5Ac(1)
IAAARVNNSGE
Intensity
m/z
5.00e+05
4.00e+05
3.00e+05
2.00e+05
1.00e+05
0.00e+00
500
1000
1500
2000
C6H8NO2
C7H8NO2
Hex
HexNAc-36
HexNAc-18
HexNAc
y2
b3
NeuAc-18
y3
HexNAcHex
b5
HexHexNAcFuc
HexNAcHexNeuAc
Pep+2HexNAc+2Hex_2+
Pep+2HexNAc+3Hex_2+
Pep+3HexNAc+3Hex_2+
Pep_1+
Pep+3HexNAc+4Hex_2+
M_2+ - HexNAcHexNeuAc
Pep+HexNAc_1+
M_2+ - HexNAcHex
Pep+HexNAc+Fuc_1+
M_2+ - HexNAc
Pep+2HexNAc_1+
Pep+2HexNAc+Fuc_1+
Pep+2HexNAc+Hex_1+
Pep+2HexNAc+2Hex_1+
Pep+2HexNAc+3Hex_1+
Pep+3HexNAc+3Hex_1+

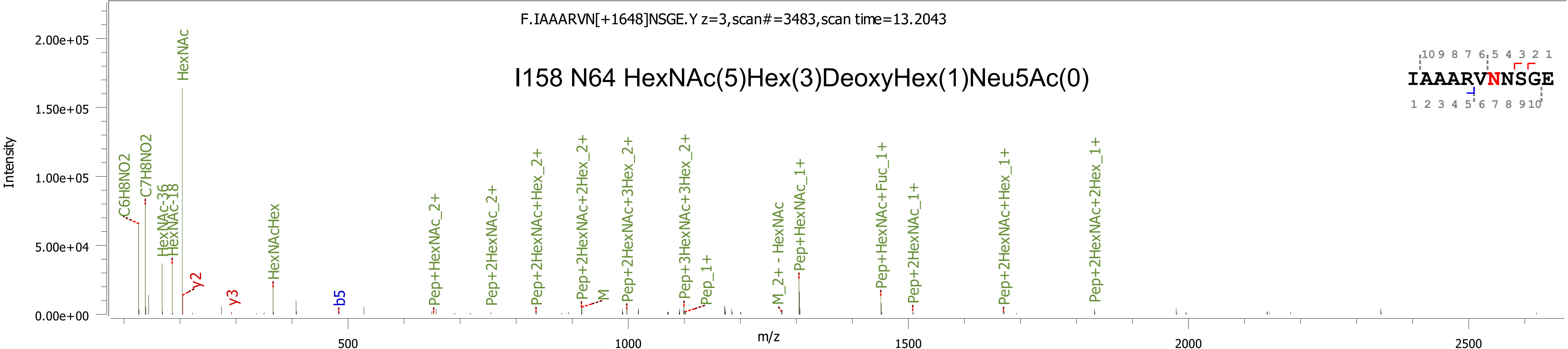
F.IAAARVN[+1648]NSGE.Y z=3,scan#=3483,scan time=13.2043
I158 N64 HexNAc(5)Hex(3)DeoxyHex(1)Neu5Ac(0)
IAAARVNNSGE
Intensity
m/z
C6H8NO2
C7H8NO2
HexNAc-36
HexNAc-18
HexNAc
y2
y3
HexNAcHex
b5
Pep+HexNAc_2+
Pep+2HexNAc_2+
Pep+2HexNAc+Hex_2+
Pep+2HexNAc+2Hex_2+
M
Pep+2HexNAc+3Hex_2+
Pep+3HexNAc+3Hex_2+
Pep_1+
M_2+ - HexNAc
Pep+HexNAc_1+
Pep+HexNAc+Fuc_1+
Pep+2HexNAc_1+
Pep+2HexNAc+Hex_1+
Pep+2HexNAc+2Hex_1+

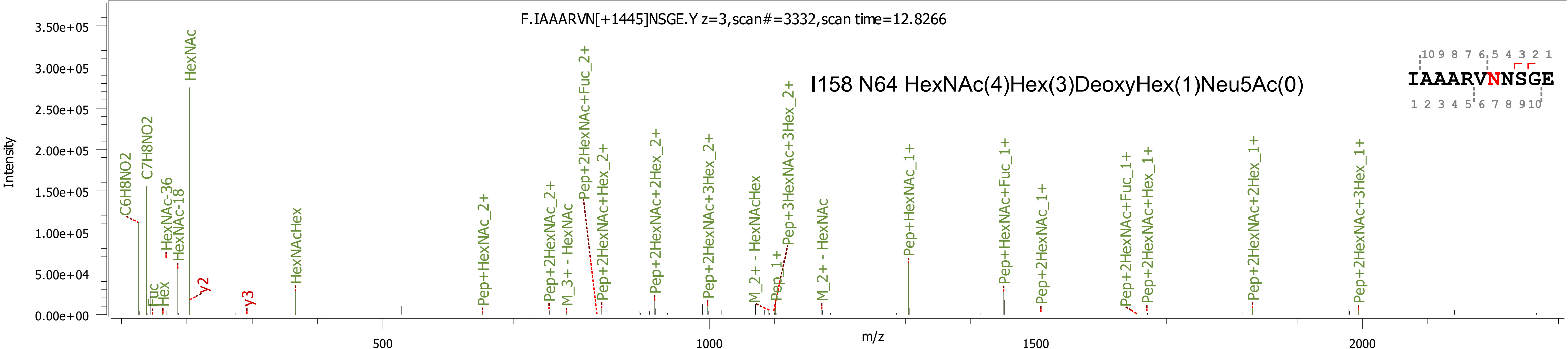

F.IAAARVN[+1445]NSGE.Y z=3,scan#=3332,scan time=12.8266
I158 N64 HexNAc(4)Hex(3)DeoxyHex(1)Neu5Ac(0)
IAAARVNNSGE
Intensity
m/z
C6H8NO2
C7H8NO2
Fuc
Hex
HexNAc-36
HexNAc-18
HexNAc
y2
y3
HexNAcHex
Pep+HexNAc_2+
Pep+2HexNAc_2+
M_3+ - HexNAc
Pep+2HexNAc+Fuc_2+
Pep+2HexNAc+Hex_2+
Pep+2HexNAc+2Hex_2+
Pep+2HexNAc+3Hex_2+
M_2+ - HexNAcHex
Pep_1+
Pep+3HexNAc+3Hex_2+
M_2+ - HexNAc
Pep+HexNAc_1+
Pep+HexNAc+Fuc_1+
Pep+2HexNAc_1+
Pep+2HexNAc+Fuc_1+
Pep+2HexNAc+Hex_1+
Pep+2HexNAc+2Hex_1+
Pep+2HexNAc+3Hex_1+

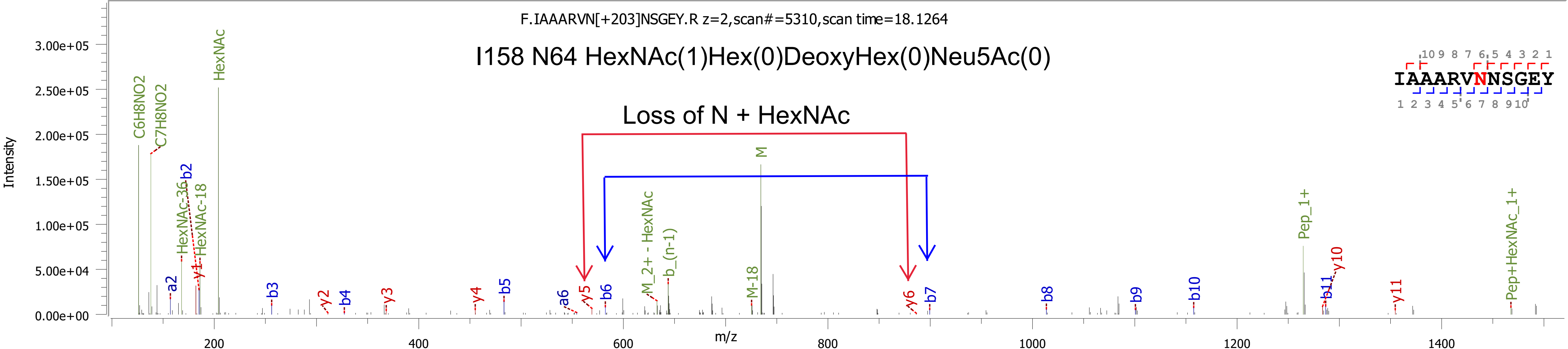

F.IAAARVN[+203]NSGEY.R z=2,scan#=5310,scan time=18.1264
I158 N64 HexNAc(1)Hex(0)DeoxyHex(0)Neu5Ac(0)
Loss of N + HexNAc
IAAARVNNSGEY
Intensity
m/z
C6H8NO2
C7H8NO2
HexNAc
HexNAc-36
HexNAc-18
b2
a2
y1
b3
y2
b4
y3
y4
b5
a6
y5
b6
M_2+ - HexNAc
b_(n-1)
M
M-18
y6
b7
b8
b9
b10
Pep_1+
b11
y10
y11
Pep+HexNAc_1+

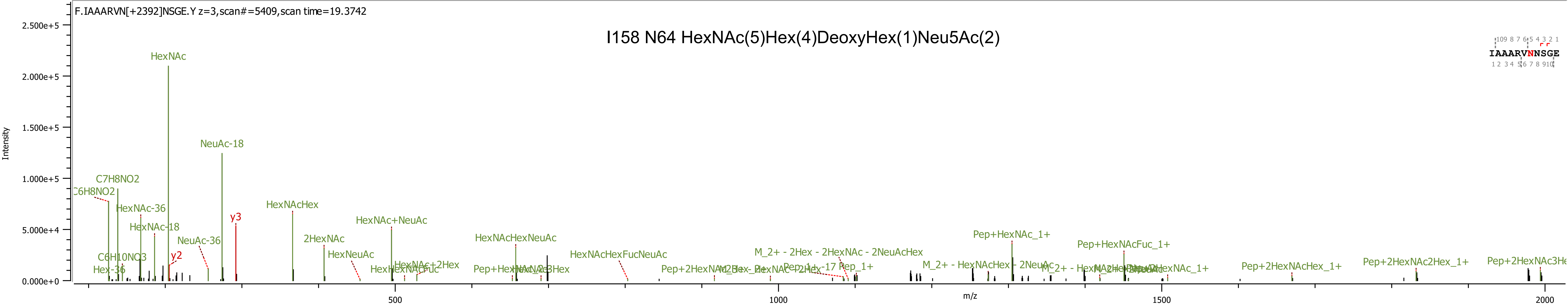
F.IAAARVN[+2392]NSGE.Y z=3,scan#=5409,scan time=19.3742
I158 N64 HexNAc(5)Hex(4)DeoxyHex(1)Neu5Ac(2)
IAAARVNNSGE
Intensity
m/z
HexNAc
NeuAc-18
C7H8NO2
C6H8NO2
HexNAc-36
HexNAc-18
HexNAcHex
y3
NeuAc-36
y2
2HexNAc
HexNAc+NeuAc
HexNeuAc
C6H10NO3
Hex-36
HexNAcHexNeuAc
HexNAcHexFucNeuAc
Pep+HexNAc_1+
Pep+HexNAcFuc_1+
M_2+ - 2Hex - 2HexNAc - 2NeuAcHex
Pep+2HexNAcHex_1+
Pep+2HexNAc2Hex_1+
Pep_1+
Pep+2HexNAc_1+

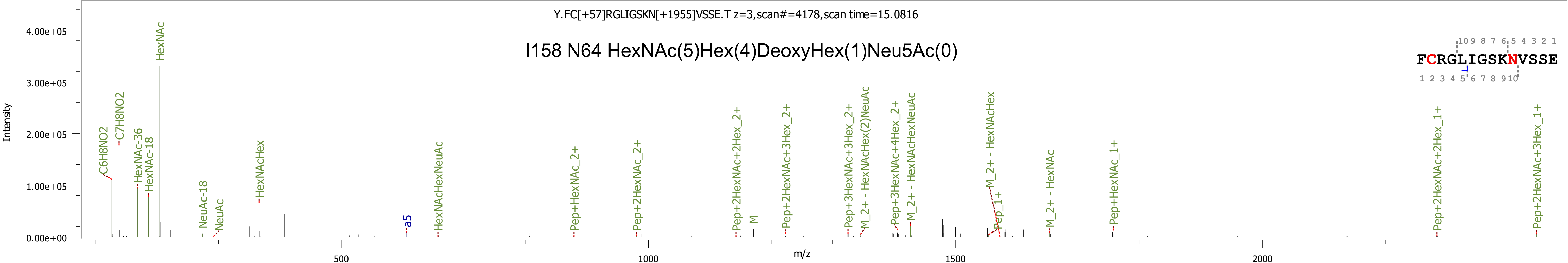
Y.FC[+57]RGLIGSKN[+1955]VSSE.T z=3,scan#=4178,scan time=15.0816
I158 N64 HexNAc(5)Hex(4)DeoxyHex(1)Neu5Ac(0)
FCRGLIGSKNVSSE
Intensity
m/z
4.00e+05
3.00e+05
2.00e+05
1.00e+05
0.00e+00
500
1000
1500
2000
C6H8NO2
C7H8NO2
HexNAc-36
HexNAc-18
HexNAc
NeuAc-18
NeuAc
HexNAcHex
a5
HexNAcHexNeuAc
Pep+HexNAc_2+
Pep+2HexNAc_2+
Pep+2HexNAc+2Hex_2+
M
Pep+2HexNAc+3Hex_2+
Pep+3HexNAc+3Hex_2+
M_2+ - HexNAcHex(2)NeuAc
Pep+3HexNAc+4Hex_2+
M_2+ - HexNAcHexNeuAc
M_2+ - HexNAcHex
Pep_1+
M_2+ - HexNAc
Pep+HexNAc_1+
Pep+2HexNAc+2Hex_1+
Pep+2HexNAc+3Hex_1+

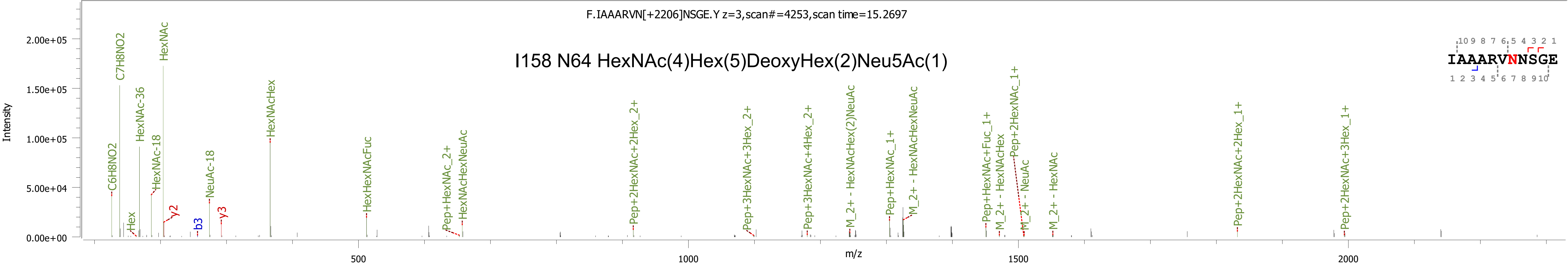
F.IAAARVN[+2206]NSGE.Y z=3,scan#=4253,scan time=15.2697
I158 N64 HexNAc(4)Hex(5)DeoxyHex(2)Neu5Ac(1)
IAAARVNNSGE
Intensity
m/z
C6H8NO2
C7H8NO2
Hex
HexNAc-36
HexNAc-18
HexNAc
y2
b3
NeuAc-18
y3
HexNAcHex
HexHexNAcFuc
Pep+HexNAc_2+
HexNAcHexNeuAc
Pep+2HexNAc+2Hex_2+
Pep+3HexNAc+3Hex_2+
Pep+3HexNAc+4Hex_2+
M_2+ - HexNAcHex(2)NeuAc
Pep+HexNAc_1+
M_2+ - HexNAcHexNeuAc
Pep+HexNAc+Fuc_1+
M_2+ - HexNAcHex
Pep+2HexNAc_1+
M_2+ - NeuAc
M_2+ - HexNAc
Pep+2HexNAc+2Hex_1+
Pep+2HexNAc+3Hex_1+

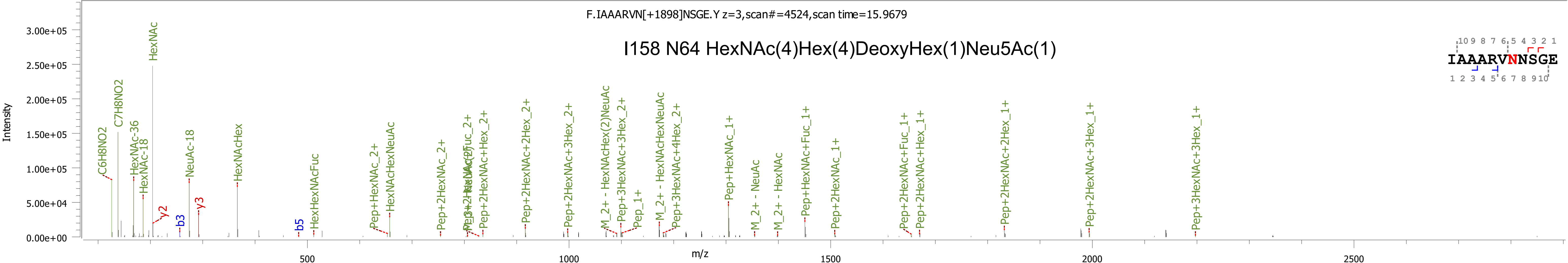
F.IAAARVN[+1898]NSGE.Y z=3,scan#=4524,scan time=15.9679
I158 N64 HexNAc(4)Hex(4)DeoxyHex(1)Neu5Ac(1)
IAAARVNNSGE
Intensity
m/z
3.00e+05
2.50e+05
2.00e+05
1.50e+05
1.00e+05
5.00e+04
0.00e+00
500
1000
1500
2000
2500
C6H8NO2
C7H8NO2
HexNAc-36
HexNAc-18
HexNAc
y2
b3
NeuAc-18
y3
HexNAcHex
b5
HexHexNAcFuc
Pep+HexNAc_2+
HexNAcHexNeuAc
Pep+2HexNAc_2+
Pep+2HexNAc+Hex_2+
Pep+2HexNAc+2Hex_2+
Pep+2HexNAc+3Hex_2+
M_2+ - HexNAcHex(2)NeuAc
Pep+3HexNAc+3Hex_2+
Pep_1+
M_2+ - HexNAcHexNeuAc
Pep+3HexNAc+4Hex_2+
Pep+HexNAc_1+
M_2+ - NeuAc
M_2+ - HexNAc
Pep+HexNAc+Fuc_1+
Pep+2HexNAc_1+
Pep+2HexNAc+Fuc_1+
Pep+2HexNAc+Hex_1+
Pep+2HexNAc+2Hex_1+
Pep+2HexNAc+3Hex_1+
Pep+3HexNAc+3Hex_1+

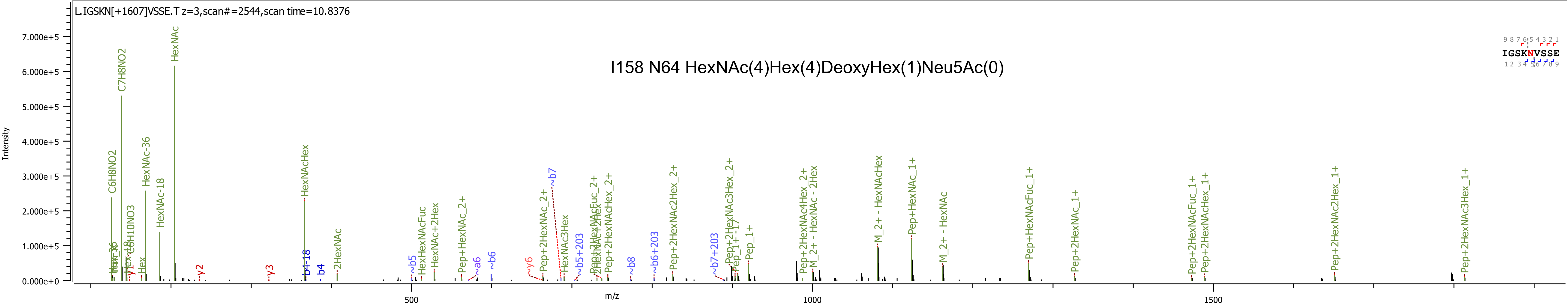

L.IGSKN[+1607]VSSE.T z=3,scan#=2544,scan time=10.8376
I158 N64 HexNAc(4)Hex(4)DeoxyHex(1)Neu5Ac(0)
IGSKNVSSE
Intensity
m/z
C7H8NO2
HexNAc
C6H8NO2
HexNAc-36
HexNAc-18
HexNAcHex
2HexNAc
HexHexNAcFuc
HexNAc+2Hex
Pep+HexNAc_2+
Pep+2HexNAc_2+
HexNAc3Hex
Pep+2HexNAcHex_2+
Pep+2HexNAc2Hex_2+
Pep+2HexNAc3Hex_2+
Pep_1+
Pep+2HexNAc4Hex_2+
M_2+ - HexNAc - 2Hex
M_2+ - HexNAcHex
Pep+HexNAc_1+
M_2+ - HexNAc
Pep+HexNAcFuc_1+
Pep+2HexNAc_1+
Pep+2HexNAcFuc_1+
Pep+2HexNAcHex_1+
Pep+2HexNAc2Hex_1+
Pep+2HexNAc3Hex_1+
y1
y2
y3
y6
b4-18
b4
b5
a6
b6
b7
b5+203
b8
b6+203
b7+203
500
1000
1500

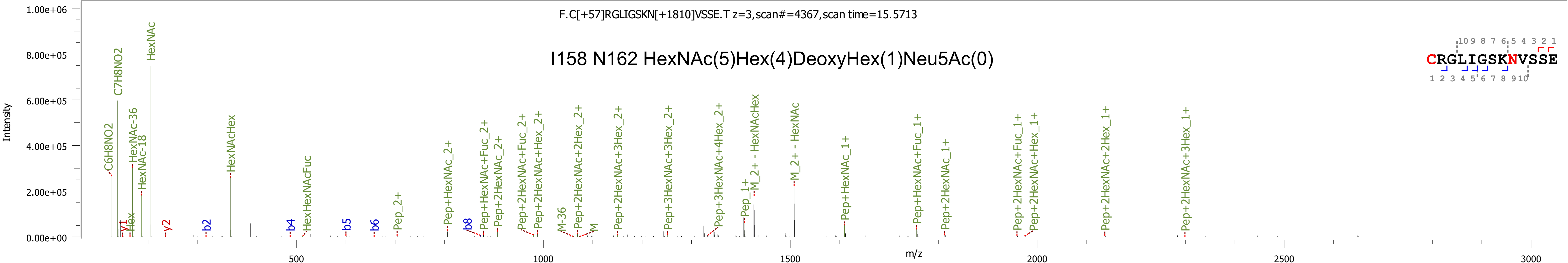

F.C[+57]RGLIGSKN[+1810]VSSE.T z=3,scan#=4367,scan time=15.5713
I158 N162 HexNAc(5)Hex(4)DeoxyHex(1)Neu5Ac(0)
CRGLIGSKNVSSE
Intensity
m/z
C6H8NO2
C7H8NO2
HexNAc-36
HexNAc-18
HexNAc
Hex
y1
y2
b2
HexNAcHex
b4
HexHexNAcFuc
b5
b6
Pep_2+
Pep+HexNAc_2+
b8
Pep+HexNAc+Fuc_2+
Pep+2HexNAc_2+
Pep+2HexNAc+Fuc_2+
Pep+2HexNAc+Hex_2+
M-36
Pep+2HexNAc+2Hex_2+
M
Pep+2HexNAc+3Hex_2+
Pep+3HexNAc+3Hex_2+
Pep+3HexNAc+4Hex_2+
Pep_1+
M_2+ - HexNAcHex
M_2+ - HexNAc
Pep+HexNAc_1+
Pep+HexNAc+Fuc_1+
Pep+2HexNAc_1+
Pep+2HexNAc+Fuc_1+
Pep+2HexNAc+Hex_1+
Pep+2HexNAc+2Hex_1+
Pep+2HexNAc+3Hex_1+

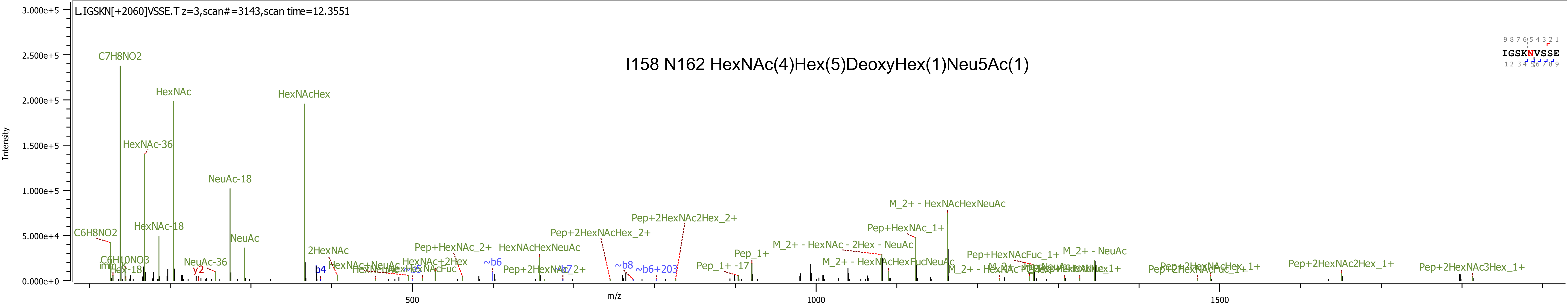
L.IGSKN[+2060]VSSE.T z=3,scan#=3143,scan time=12.3551
I158 N162 HexNAc(4)Hex(5)DeoxyHex(1)Neu5Ac(1)
IGSKNVSSE
Intensity
m/z
3.000e+5
2.500e+5
2.000e+5
1.500e+5
1.000e+5
5.000e+4
0.000e+0
500
1000
1500
C7H8NO2
HexNAc
HexNAcHex
HexNAc-36
NeuAc-18
HexNAc-18
C6H8NO2
NeuAc
NeuAc-36
C6H10NO3
y2
b4
2HexNAc
Pep+HexNAc_2+
HexNAcHexNeuAc
HexNAc+2Hex
~b6
Pep+2HexNAcHex_2+
Pep+2HexNAc2Hex_2+
~b8
~b6+203
Pep_1+
Pep_1+ -17
M_2+ - HexNAc - 2Hex - NeuAc
M_2+ - HexNAcHexFucNeuAc
Pep+HexNAc_1+
M_2+ - HexNAcHexNeuAc
Pep+HexNAcFuc_1+
M_2+ - NeuAc
Pep+2HexNAc2Hex_1+
Pep+2HexNAc3Hex_1+

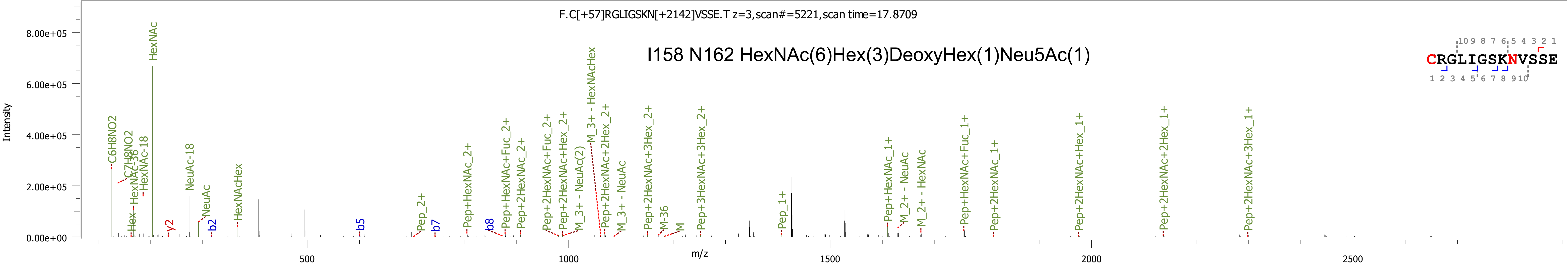
F.C[+57]RGLIGSKN[+2142]VSSE.T z=3,scan#=5221,scan time=17.8709
I158 N162 HexNAc(6)Hex(3)DeoxyHex(1)Neu5Ac(1)
CRGLIGSKNVSSE
Intensity
m/z
0.00e+00
2.00e+05
4.00e+05
6.00e+05
8.00e+05
500
1000
1500
2000
2500
C6H8NO2
C7H8NO2
Hex
HexNAc-36
HexNAc-18
HexNAc
y2
NeuAc-18
NeuAc
b2
HexNAcHex
b5
Pep_2+
b7
Pep+HexNAc_2+
b8
Pep+HexNAc+Fuc_2+
Pep+2HexNAc_2+
Pep+2HexNAc+Fuc_2+
Pep+2HexNAc+Hex_2+
M_3+ - NeuAc(2)
M_3+ - HexNAcHex
Pep+2HexNAc+2Hex_2+
M_3+ - NeuAc
Pep+2HexNAc+3Hex_2+
M-36
M
Pep+3HexNAc+3Hex_2+
Pep_1+
Pep+HexNAc_1+
M_2+ - NeuAc
M_2+ - HexNAc
Pep+HexNAc+Fuc_1+
Pep+2HexNAc_1+
Pep+2HexNAc+Hex_1+
Pep+2HexNAc+2Hex_1+
Pep+2HexNAc+3Hex_1+

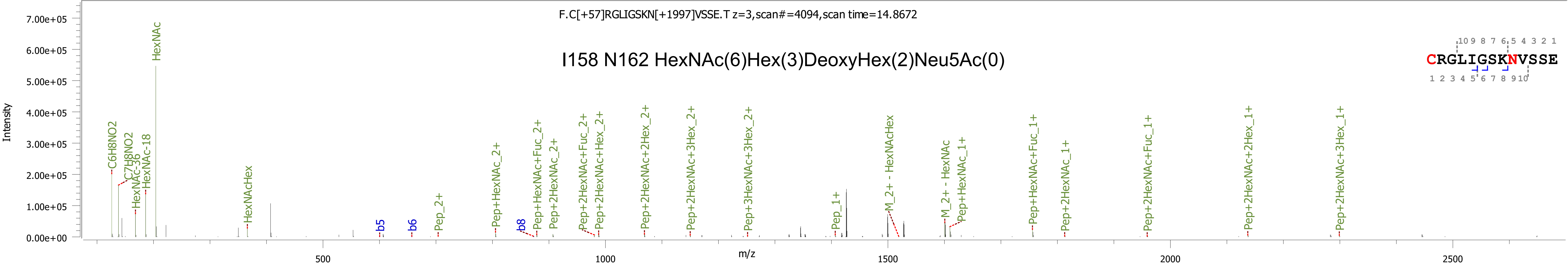
F.C[+57]RGLIGSKN[+1997]VSSE.T z=3,scan#=4094,scan time=14.8672
I158 N162 HexNAc(6)Hex(3)DeoxyHex(2)Neu5Ac(0)
CRGLIGSKNVSSE
Intensity
m/z
C6H8NO2
C7H8NO2
HexNAc-36
HexNAc-18
HexNAc
HexNAcHex
b5
b6
Pep_2+
Pep+HexNAc_2+
b8
Pep+HexNAc+Fuc_2+
Pep+2HexNAc_2+
Pep+2HexNAc+Fuc_2+
Pep+2HexNAc+Hex_2+
Pep+2HexNAc+2Hex_2+
Pep+2HexNAc+3Hex_2+
Pep+3HexNAc+3Hex_2+
Pep_1+
M_2+ - HexNAcHex
M_2+ - HexNAc
Pep+HexNAc_1+
Pep+HexNAc+Fuc_1+
Pep+2HexNAc_1+
Pep+2HexNAc+Fuc_1+
Pep+2HexNAc+2Hex_1+
Pep+2HexNAc+3Hex_1+

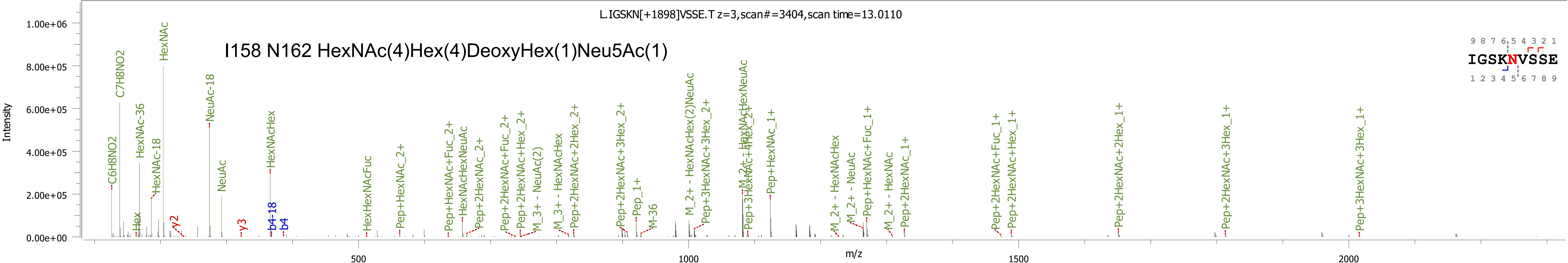

L.IGSKN[+1898]VSSE.T z=3,scan#=3404,scan time=13.0110
I158 N162 HexNAc(4)Hex(4)DeoxyHex(1)Neu5Ac(1)
Intensity
m/z
IGSKNVSSE
C6H8NO2
C7H8NO2
Hex
HexNAc-36
HexNAc-18
HexNAc
y2
NeuAc-18
NeuAc
y3
HexNAcHex
b4-18
b4
HexHexNAcFuc
Pep+HexNAc_2+
Pep+HexNAc+Fuc_2+
HexNAcHexNeuAc
Pep+2HexNAc_2+
Pep+2HexNAc+Fuc_2+
Pep+2HexNAc+Hex_2+
M_3+ - NeuAc(2)
M_3+ - HexNAcHex
Pep+2HexNAc+2Hex_2+
Pep+2HexNAc+3Hex_2+
Pep_1+
M-36
M_2+ - HexNAcHex(2)NeuAc
Pep+3HexNAc+3Hex_2+
Pep+HexNAc_1+
M_2+ - HexNAcHex
M_2+ - NeuAc
Pep+HexNAc+Fuc_1+
M_2+ - HexNAc
Pep+2HexNAc_1+
Pep+2HexNAc+Fuc_1+
Pep+2HexNAc+Hex_1+
Pep+2HexNAc+2Hex_1+
Pep+2HexNAc+3Hex_1+
Pep+3HexNAc+3Hex_1+

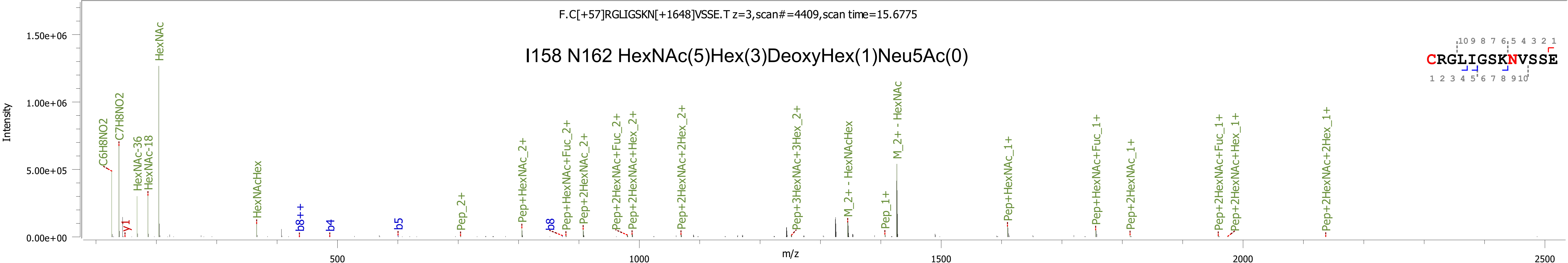

F.C[+57]RGLIGSKN[+1648]VSSE.T z=3,scan#=4409,scan time=15.6775
I158 N162 HexNAc(5)Hex(3)DeoxyHex(1)Neu5Ac(0)
CRGLIGSKNVSSE
Intensity
m/z
C6H8NO2
C7H8NO2
y1
HexNAc-36
HexNAc-18
HexNAc
HexNAcHex
b8++
b4
b5
Pep_2+
Pep+HexNAc_2+
b8
Pep+HexNAc+Fuc_2+
Pep+2HexNAc_2+
Pep+2HexNAc+Fuc_2+
Pep+2HexNAc+Hex_2+
Pep+2HexNAc+2Hex_2+
Pep+3HexNAc+3Hex_2+
M_2+ - HexNAcHex
Pep_1+
M_2+ - HexNAc
Pep+HexNAc_1+
Pep+HexNAc+Fuc_1+
Pep+2HexNAc_1+
Pep+2HexNAc+Fuc_1+
Pep+2HexNAc+Hex_1+
Pep+2HexNAc+2Hex_1+

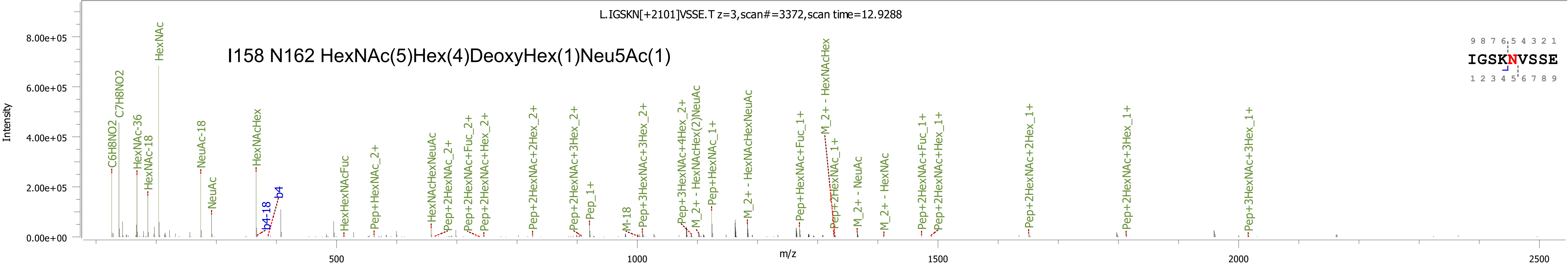
L.IGSKN[+2101]VSSE.T z=3,scan#=3372,scan time=12.9288
I158 N162 HexNAc(5)Hex(4)DeoxyHex(1)Neu5Ac(1)
IGSKNVSSE
Intensity
m/z
8.00e+05
6.00e+05
4.00e+05
2.00e+05
0.00e+00
500
1000
1500
2000
2500
C6H8NO2
C7H8NO2
HexNAc-36
HexNAc-18
HexNAc
NeuAc-18
NeuAc
HexNAcHex
b4-18
b4
HexHexNAcFuc
Pep+HexNAc_2+
HexNAcHexNeuAc
Pep+2HexNAc_2+
Pep+2HexNAc+Fuc_2+
Pep+2HexNAc+Hex_2+
Pep+2HexNAc+2Hex_2+
Pep+2HexNAc+3Hex_2+
Pep_1+
M-18
Pep+3HexNAc+3Hex_2+
Pep+3HexNAc+4Hex_2+
M_2+ - HexNAcHex(2)NeuAc
Pep+HexNAc_1+
M_2+ - HexNAcHexNeuAc
Pep+HexNAc+Fuc_1+
M_2+ - HexNAcHex
Pep+2HexNAc_1+
M_2+ - NeuAc
M_2+ - HexNAc
Pep+2HexNAc+Fuc_1+
Pep+2HexNAc+Hex_1+
Pep+2HexNAc+2Hex_1+
Pep+2HexNAc+3Hex_1+
Pep+3HexNAc+3Hex_1+

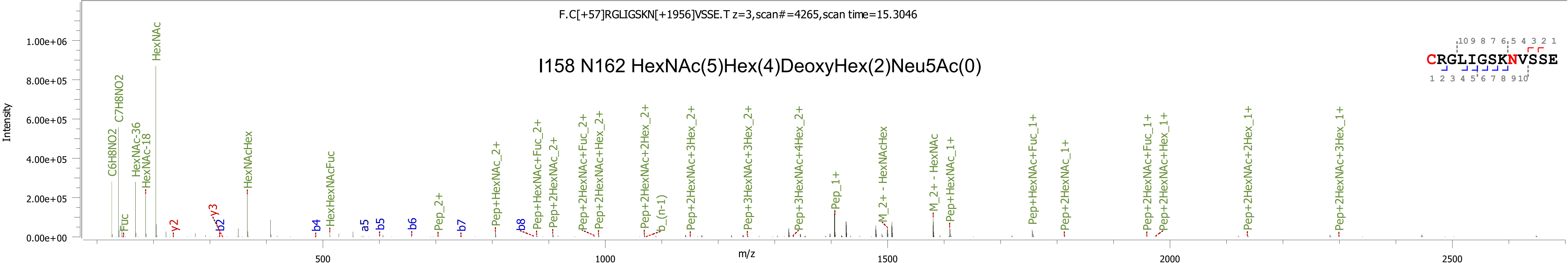

F.C[+57]RGLIGSKN[+1956]VSSE.T z=3,scan#=4265,scan time=15.3046
I158 N162 HexNAc(5)Hex(4)DeoxyHex(2)Neu5Ac(0)
CRGLIGSKNVSSE
Intensity
m/z
C6H8NO2
C7H8NO2
Fuc
HexNAc-36
HexNAc-18
HexNAc
y2
y3
b2
HexNAcHex
b4
HexHexNAcFuc
a5
b5
b6
Pep_2+
b7
Pep+HexNAc_2+
b8
Pep+HexNAc+Fuc_2+
Pep+2HexNAc_2+
Pep+2HexNAc+Fuc_2+
Pep+2HexNAc+Hex_2+
Pep+2HexNAc+2Hex_2+
b_(n-1)
Pep+2HexNAc+3Hex_2+
Pep+3HexNAc+3Hex_2+
Pep+3HexNAc+4Hex_2+
Pep_1+
M_2+ - HexNAcHex
M_2+ - HexNAc
Pep+HexNAc_1+
Pep+HexNAc+Fuc_1+
Pep+2HexNAc_1+
Pep+2HexNAc+Fuc_1+
Pep+2HexNAc+Hex_1+
Pep+2HexNAc+2Hex_1+
Pep+2HexNAc+3Hex_1+

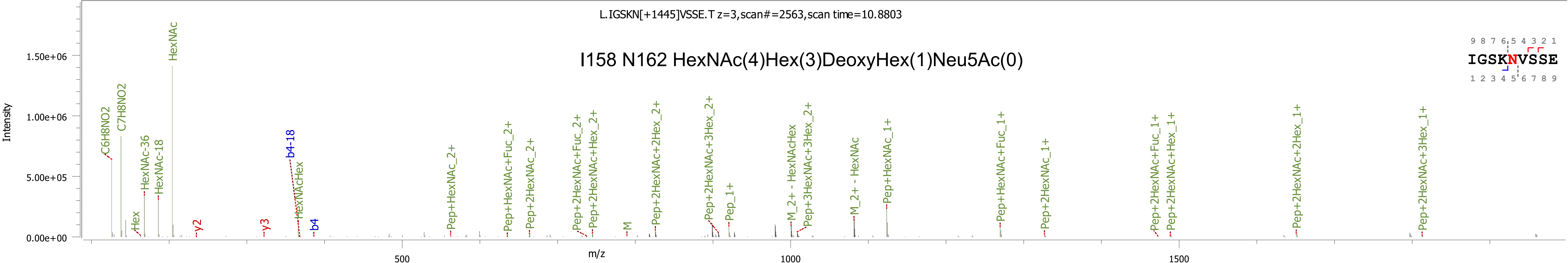
L.IGSKN[+1445]VSSE.T z=3,scan#=2563,scan time=10.8803
I158 N162 HexNAc(4)Hex(3)DeoxyHex(1)Neu5Ac(0)
IGSKNVSSE
Intensity
m/z
C6H8NO2
C7H8NO2
Hex
HexNAc-36
HexNAc-18
HexNAc
y2
y3
b4-18
HexNAcHex
b4
Pep+HexNAc_2+
Pep+HexNAc+Fuc_2+
Pep+2HexNAc_2+
Pep+2HexNAc+Fuc_2+
Pep+2HexNAc+Hex_2+
M
Pep+2HexNAc+2Hex_2+
Pep+2HexNAc+3Hex_2+
Pep_1+
M_2+ - HexNAcHex
Pep+3HexNAc+3Hex_2+
M_2+ - HexNAc
Pep+HexNAc_1+
Pep+HexNAc+Fuc_1+
Pep+2HexNAc_1+
Pep+2HexNAc+Fuc_1+
Pep+2HexNAc+Hex_1+
Pep+2HexNAc+2Hex_1+
Pep+2HexNAc+3Hex_1+

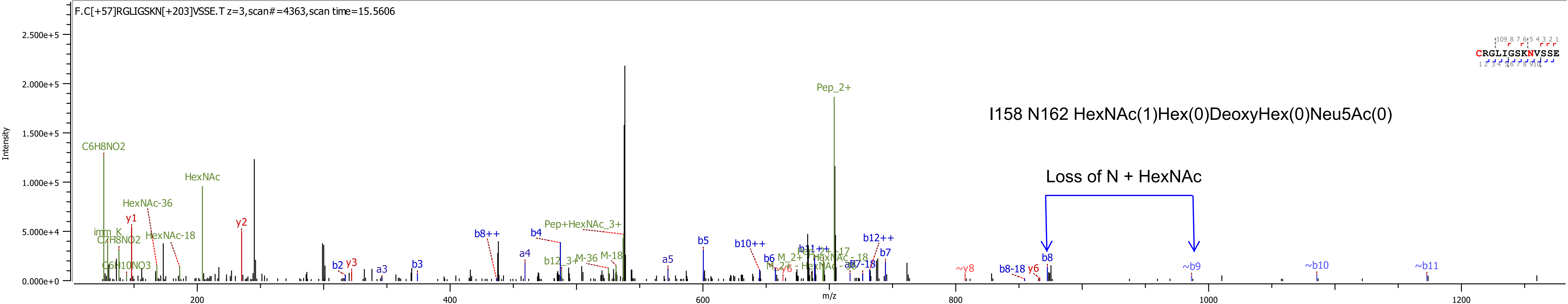
F.C[+57]RGLIGSKN[+203]VSSE.T z=3,scan#=4363,scan time=15.5606
I158 N162 HexNAc(1)Hex(0)DeoxyHex(0)Neu5Ac(0)
Loss of N + HexNAc
Intensity
m/z
CRGLIGSKNVSSE
C6H8NO2
HexNAc
HexNAc-36
HexNAc-18
y1
y2
y3
b2
a3
b3
b8++
b4
a4
Pep+HexNAc_3+
b12_3+
M-36
M-18
a5
b5
b10++
b6
b11++
b12++
b7
Pep_2+
~y8
b8-18
y6
b8
~b9
~b10
~b11
